# Performance of quantum chemistry methods for a benchmark set of spin-state energetics derived from experimental data of 17 transition metal complexes (SSE17)†

**DOI:** 10.1039/d4sc05471g

**Published:** 2024-10-28

**Authors:** Mariusz Radoń, Gabriela Drabik, Maciej Hodorowicz, Janusz Szklarzewicz

**Affiliations:** a Jagiellonian University, Faculty of Chemistry Gronostajowa 2 30-387 Kraków Poland mradon@chemia.uj.edu.pl +48 12 686 24 89; b Jagiellonian University, Doctoral School of Exact and Natural Sciences Łojasiewicza 11 30-348 Kraków Poland

## Abstract

Accurate prediction of spin-state energetics for transition metal (TM) complexes is a compelling problem in applied quantum chemistry, with enormous implications for modeling catalytic reaction mechanisms and computational discovery of materials. Computed spin-state energetics are strongly method-dependent and credible reference data are scarce, making it difficult to conduct conclusive computational studies of open-shell TM systems. Here, we present a novel benchmark set of first-row TM spin-state energetics, which is derived from experimental data of 17 complexes containing Fe^II^, Fe^III^, Co^II^, Co^III^, Mn^II^, and Ni^II^ with chemically diverse ligands. The estimates of adiabatic or vertical spin-state splittings, which are obtained from spin crossover enthalpies or energies of spin-forbidden absorption bands, suitably back-corrected for the vibrational and environmental effects, are employed as reference values for benchmarking density functional theory (DFT) and wave function methods. The results demonstrate a high accuracy of the coupled-cluster CCSD(T) method, which features the mean absolute error (MAE) of 1.5 kcal mol^−1^ and maximum error of −3.5 kcal mol^−1^, and outperforms all the tested multireference methods: CASPT2, MRCI+Q, CASPT2/CC and CASPT2+δMRCI. Switching from Hartree–Fock to Kohn–Sham orbitals is not found to consistently improve the CCSD(T) accuracy. The best performing DFT methods are double-hybrids (PWPB95-D3(BJ), B2PLYP-D3(BJ)) with the MAEs below 3 kcal mol^−1^ and maximum errors within 6 kcal mol^−1^, whereas the DFT methods so far recommended for spin states (*e.g.*, B3LYP*-D3(BJ) and TPSSh-D3(BJ)) are found to perform much worse with the MAEs of 5–7 kcal mol^−1^ and maximum errors beyond 10 kcal mol^−1^. This work is the first such extensive benchmark study of quantum chemistry methods for TM spin-state energetics making use of experimental reference data. The results are relevant for the proper choice of methods to characterize TM systems in computational catalysis and (bio)inorganic chemistry, and may also stimulate new developments in quantum-chemical or machine learning approaches.

## Abstract 2

The accuracy of wave function and DFT methods for spin-state energetics benchmarked against curated experimental data of transition metal complexes.

## Graphical abstract

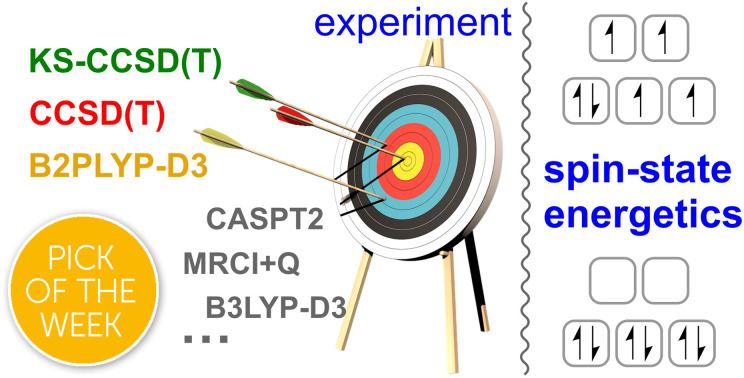

## 1 Introduction

Due to their unique electronic structures and resulting properties, transition metal (TM) complexes, as well as TM active sites in metalloproteins and nanoporous materials, are of central importance in various branches of chemistry, biochemistry and materials science.^1^ In all these areas, computational studies using quantum chemistry methods play an important role, on par with experiments, to elucidate the properties and reactivities of TM systems.^2–7^ But despite unquestionable successes, quantum chemistry methods also face some challenges when it comes to describing the properties of TM complexes with the level of accuracy required in chemical research. One of the biggest challenges that still remains is to accurately compute spin-state energetics (also known as spin-state splittings), *i.e.*, the relative energies of the alternative spin states in TM complexes.^6–11^

For mononuclear TM complexes (on which this study is focused), different spin states originate from different distributions of electrons in the manifold of d-orbitals, whose energy levels are split by interactions with the ligands.^1^ In first-row TM complexes with electronic configuration d^4^–d^8^, the low-spin (LS) and high-spin (HS) states may have comparable energies for a certain range of ligand field strengths, and hence the phenomenon of spin crossover (SCO) may occur if the spin-state splitting is small enough to be overcome by the entropic term of the Gibbs free energy.^12–14^ If the spin-state splitting is larger, the system may be optically excited to the higher-energy spin state, leading to the occurrence of weak, spin-forbidden d–d absorption features.^15–17^ The crossing of spin states may also occur along a reaction path, which has significant implications for the mechanisms of spin-forbidden reactions,^18–20^ including also examples from enzymatic catalysis^20^ and ligand binding to heme.^21,22^ Thus, one can find numerous cases in chemical research where accurate computation of spin-state energetics, particularly for first-row TMs, is of critical importance at least in the following aspects: (a) ground state prediction;^23–28^ (b) SCO prediction and estimation of the transition temperature^29–32^ or populations of different spin states for reactive species;^33^ (c) interpretation of the electronic spectra^16,17,34,35^ and magnetic properties^36,37^ of TM complexes; (d) interpretation of the kinetic^22^ or thermodynamic^38^ features in spin-forbidden reactions.^18^

As mentioned above, accurate computation of TM spin-state energetics is recognized as a grand challenge for quantum chemistry methods. A frequently occurring problem is that different methods lead to divergent and inconsistent results. This behavior is well known for approximate density functional theory (DFT) methods,^9,12,39^ but can be observed even for high-level wave function theory (WFT) methods, making it problematic to establish unambiguous reference values.^11,40^ For example, the predictions of the singlet–quintet energy gap in [Fe^II^(NCH)_6_]^2+^ (a widely studied, simplified model of SCO compounds) originating from the best available diffusion Monte Carlo (DMC)^41,42^ and coupled cluster (CC) calculations at the CCSD(T) level^43,44^ differ from each other by as much as 20 kcal mol^−1^. Various methods have been advocated in the literature by different authors for the purpose of accurately describing mononuclear TM complexes, *e.g.*, CCSD(T) or its local-correlation approximations,^43–50^ multiconfigurational perturbation theory (CASPT2)^51^ or its modifications like CASPT2/CC,^52^ CASPT2+δMRCI^53^ or CASPT2.5,^54^ multireference configuration interaction (MRCI+Q),^55^ multiconfigurational pair-density functional theory (MC-PDFT),^56,57^ density matrix renormalization group (DMRG) and DMRG-based methods^58,59^ as well as various Monte Carlo (MC) approaches, including FCIQMC,^60^ FCIQMC-tailored distinguishable cluster,^61^ AFQMC,^28,62^ and DMC.^41,42^ It is presently unclear which of these methods yield most reliable spin-state splittings, what are typical error bars of their predictions, whether one should trust more in single- or multi-reference methods and how one should interpret the discrepancies between the results of different methods.^11,28^ The difficulty of obtaining indisputably accurate spin-state energetics from theory and the scarcity of reliable benchmark studies significantly impair our ability to carry out conclusive computational studies of open-shell TM systems.

Whereas the majority of theoretical studies attempt to obtain benchmark-quality spin-state energetics from high-level computations (see examples above), we recently focused on the alternative strategy of deriving the reference values from appropriate experimental data.^63,64^ As recently reviewed by one of us,^11^ the experimental data which are particularly valuable in the context of method benchmarking are: (1) SCO enthalpies and (2) energies of spin-forbidden d–d optical transitions. Out of these it is possible to derive the reference values for, respectively, adiabatic (1) or vertical energy (2) differences between the involved spin states. The best strategy seems to be combining data from the above two sources in order to gather in one benchmark set the spin-state energetics of chemically diverse SCO and non-SCO complexes.^11^

Clearly, these ideas are not entirely new. The use of SCO data is relatively common in the context of DFT benchmarking, with seminal contributions of Jensen and Cirera^65^ and Kepp,^29^ followed by Cirera and Ruiz with co-workers,^30,31,66^ Vela *et al.*,^67^ Ohlrich *et al.*,^68^ and Mariano *et al.*^69^ The use of spin-forbidden d–d transition energies has been pioneered by Hughes and Friesner,^70^ who also pointed out that these spectral data allow probing a more diverse range of ligand field strengths and TMs than is available from the SCO data. Some SCO or non-SCO experimental data have also been used occasionally for testing the accuracy of selected WFT methods (see references in our review^11^). Still, these ideas have not received sufficient attention in the literature—particularly with regard to the joint use of SCO and non-SCO data, assessing the accuracy of WFT and DFT methods simultaneously based on one common benchmark set, and taking into account appropriate corrections for vibrational and environmental effects—before our first benchmark study of four octahedral Fe complexes^63^ and subsequent study of metallocenes.^64^ One obvious limitation of the mentioned studies, which we would like to eliminate now, was the small number of studied complexes, leading to potential concerns about the representability of these benchmarks.

**Fig. 1:**
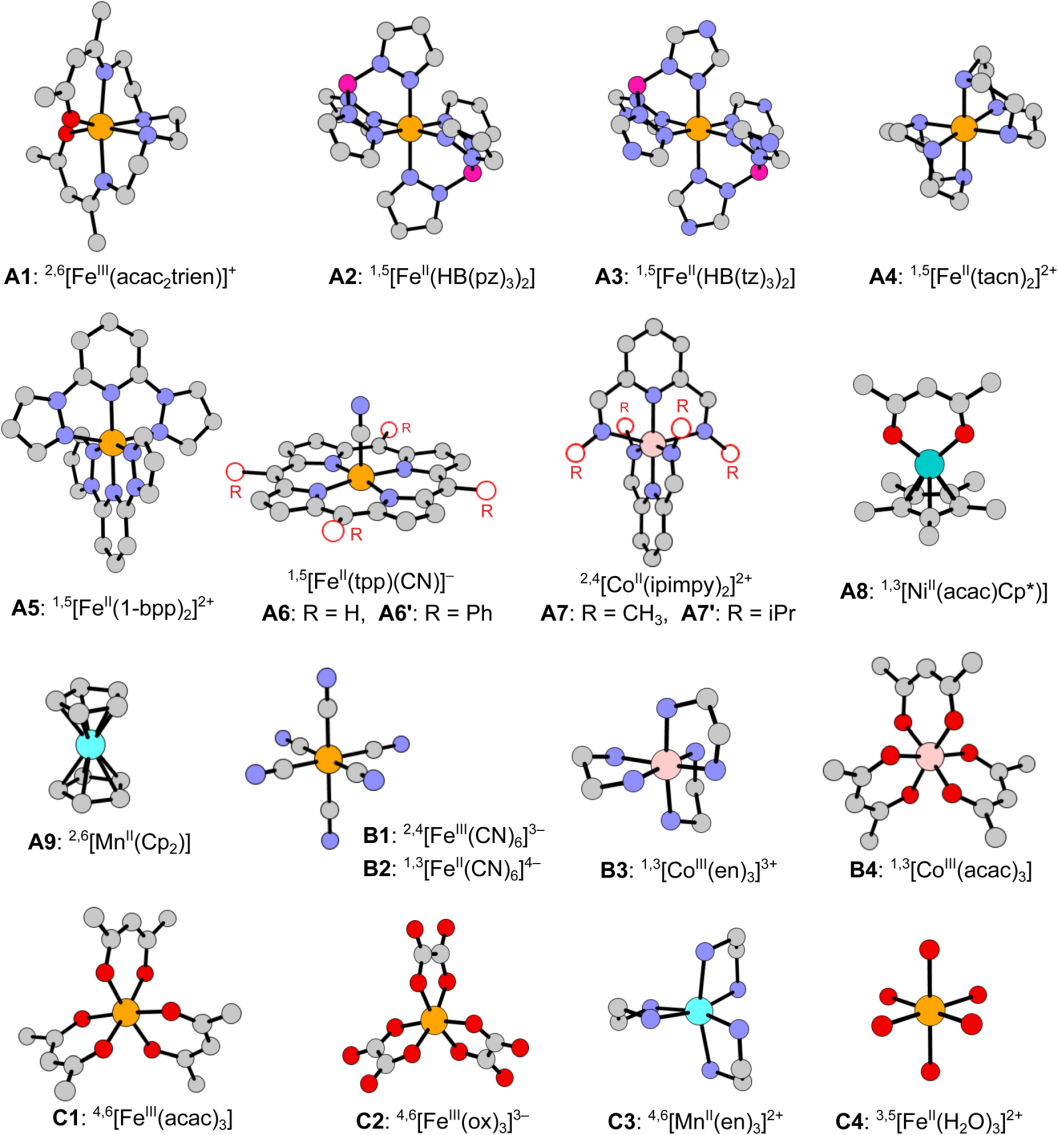
Molecular structures of 17 complexes studied in this work (hydrogens omitted for clarity): A1**–**A9 SCO complexes, B1**–**B4 complexes with LS ground state, C1**–**C4 complexes with HS ground state. Multiplicities of the considered spin states are given in the superscript. Ligand abbreviations: acac_2_trien = dianion of Schiff base obtained from the 2 : 1 condensation of acetylacetone with triethylenetetramine; HB(pz)_3_ = hydrotris(pyrazol-1-yl)borate; HB(tz)_3_ = hydrotris(1,2,4-triazol-1-yl)borate; tacn = 1,4,7-triazacyclononane; 1-bpp = 2,6-di(pyrazol-1-yl)pyridine; tpp = tetraphenylporphyrin; ipimpy = 2,6-bis(isopropyliminomethyl)pyridine; acac = acetylacetonate; Cp = cyclopentadienyl; Cp* = pentamethylcyclopentadienyl; en = ethylenediamine; ox = oxalate.

In this work we develop a novel benchmark set of spin-state energetics (SSE17), which is based on the experimental data of 17 first-row TM complexes: enthalpy differences for 9 SCO complexes (A1**–**A9) and spin-forbidden absorption maxima for 8 non-SCO complexes (B1**–**B4, C1**–**C4). The molecular structures of all complexes are shown in Fig. 1. The present set of TM complexes is not only larger than in the previous studies,^63,64^ but also more balanced considering the diversity of TM ions (Fe^II^, Fe^III^, Co^II^, Co^III^, Mn^II^, Ni^II^), ligand-field strength and coordination architecture. The most important class of Fe^II^ SCO complexes is decently represented by 5 items (A2**–**A6), but does not dominate the entire set as we also include SCO complexes of Fe^III^ (A1), Co^II^ (A7), Ni^II^ (A8), and Mn^II^ (A9). Non-SCO complexes with LS ground state (B1**–**B4) and HS ground state (C1**–**C4) are evenly represented, accounting for the range of strong and weak ligand fields, in which the most common singly spin-forbidden transitions are observed: Fe^II^ doublet–quartet (B1), Fe^II^ and Co^III^ singlet–triplet (B2**–**B4), Fe^III^ and Mn^II^ sextet–quartet (C1**–**C3), and Fe^II^ quintet–triplet (C4). The selection of complexes is dictated by the availability of credible experimental data and the possibility of performing most expensive WFT calculations, including canonical CCSD(T). The latter condition, with our recently developed protocols to efficiently estimate the complete basis set (CBS) limit,^71^ presently restricts the molecular size to *ca.* 50 atoms.

When deriving electronic spin-state splittings from the experimental data, it is necessary to back-correct for vibrational and environmental corrections, which can reach up to several kcal mol^−1^ in magnitude.^11^ The vibrational correction originates from the change of vibrational frequencies with the change of spin state. The environmental correction describes the effect of solvation or crystal packing on the investigated spin-state splitting as compared with that of isolated molecules. We use state-of-the-art approaches for estimating both corrections. We now also introduce some improvements related to evaluation of these corrections and the usage of experimental data. Firstly, wherever possible we now include data for SCO complexes in multiple environments, *i.e.*, crystal and solution or solutions in different solvents, in order to obtain more reliable averaged back-corrected values and estimate the uncertainty related to determination of the environmental correction from the spread of different back-corrected values. Secondly, employing the vibronic simulation approach introduced in ref. 64, we now include the vibrational correction also for vertical transitions, which leads to a more balanced treatment of non-SCO and SCO data. Thirdly, with the aim of avoiding large environmental corrections previously observed for vertical energies in ionic complexes,^63^ we now use reference geometries optimized within an electrostatic screening model as they are closer to experimental condensed-phase geometries.^72^ Finally, recognizing pronounced sensitivity of vertical excitation energies to the quality of molecular geometries^64^ and the difficulty of computing these geometries with sufficient accuracy for TM complexes in solution, we decided to include now only the data of spin-forbidden d–d transitions measured for solid-state compounds with known crystal structures. For such cases, the experimental crystal structure will be directly used to calculate the environmental correction, thereby alleviating the mentioned sensitivity problem. We use diffuse reflectance spectroscopy^73^ to measure spin-forbidden d–d transitions for complexes B1**–**B4, C1**–**C4 in solid state. To satisfy the constraint of having simultaneously the spectra and crystal structures available for identical solid-state compounds and recognizing the scarcity of appropriate data in the literature, we decided, specially for developing the SSE17 benchmark set, to record most of the required reflectance spectra and to obtain a crystal structure of a new compound [Mn(en)_3_]Cl_3_·H_2_O (1) containing HS Mn^II^ complex C3.

This paper is organized as follows. After presenting some necessary methodology details, the Results and discussion section describes the SSE17 benchmark set, including the experimental data and applied corrections, based on which the reference spin-state splittings are derived. The SSE17 reference data are subsequently used to benchmark the accuracy of selected WFT and DFT methods, thereby providing us with statistically relevant conclusions on their performance for the spin-state energetics of mononuclear first-row TM complexes.

## 2 Computational and experimental methods

### 2.1 DFT calculations

#### 2.1.1 Geometry optimizations

Geometries of complexes comprising the SSE17 set were optimized at the PBE0 ^74^-D3(BJ)^75^/def2-TZVP^76^ level using Turbomole v7.5.^77,78^ The COSMO model^79^ (with *ε* = ∞^80^) was used to describe the electrostatic screening effect of a condensed phase on molecular geometries of TM complexes.^11,72^ Details of the COSMO calculations are given in Section S2.1, ESI.† Both spin states were optimized for SCO complexes (A1**–**A9) or only the ground state for others (LS for B1**–**B4, HS for C1**–**C4). Jahn–Teller (JT) geometry distortions in degenerate electronic states were accounted for by properly reducing the computational symmetry, where applicable. It has been verified by running frequency calculations that the optimized geometries are energy minima (or very close to them for A9, see the ESI†).

#### 2.1.2 Single-point energy calculations

Employing the optimized COSMO/PBE0-D3(BJ)/def2-TZVP geometries (see above), subsequent single-point calculations in vacuum were performed with the def2-QZVPP basis set using 32 DFT methods (for the list of functionals, see Results and discussion) including dispersion corrections wherever available. The energies reported below include additive corrections for scalar-relativistic effects at the second-order Douglas–Kroll (DK) level^81^ calculated as described in Section S2.1, ESI.† Depending on the functional, the calculations were performed using either Turbomole,^77,78^ Gaussian 16 (ref. 82) or Orca v5.0.^83,84^ More details can be found in the ESI.†

### 2.2 WFT calculations

#### 2.2.1 Single-point energy calculations

Employing the optimized COSMO/PBE0-D3(BJ)/def2-TZVP geometries (see above), single-point calculations in vacuum were performed with selected WFT methods. Single-reference coupled-cluster (CC) calculations were performed at the CCSD(T) level employing Hartree–Fock (HF) orbitals in the reference Slater determinant. Alternatively, KS-CCSD(T) calculations were performed employing Kohn–Sham (KS) orbitals in the reference determinant; we compared the PBE0 and PBE orbitals, leading to methods abbreviated as PBE0-CCSD(T) and PBE-CCSD(T). All CC calculations for open-shell systems utilized the ROHF/UCCSD(T) formulation.^85,86^ Among multireference methods we used CASPT2 (IPEA shift 0.25 a.u.), CASPT2/CC,^52^ CASPT2+δMRCI,^53^ and MRCI+Q in Celani–Werner (CW) internally contracted formulation.^87^ The calculations were performed using Molpro,^88–90^ except for CASPT2 calculations performed using OpenMolcas.^91^ All valence electrons and TM 3s3p electrons were correlated.

#### 2.2.2 Basis sets and approaching complete basis set (CBS) limit

In order to efficiently approach the CBS limit in the CCSD(T) calculations, we employed our recently developed CCSD(T#)-F12a protocol,^71^ which is based on the explicitly correlated CCSD-F12a theory of Werner with co-workers,^92^ but uses a modified scaling of the perturbative triples. In the benchmark study of small TM complexes, the CCSD(T#)-F12a method in combination with a relatively small basis set cT(D), which is composed of cc-pwCVTZ for TM atom, cc-pVTZ for ligand atoms directly bound to TM atom and cc-pVDZ for the remaining ligand atoms, has been shown to reproduce the CCSD(T)/CBS limits of relative spin-state energetics to within 1 kcal mol^−1^ (mean deviation 0.2, mean absolute deviation 0.4, maximum deviation 0.8 kcal mol^−1^).^71^ Following this strategy, the best estimates of the CCSD(T) energies in the CBS limit were calculated as

1 Δ*E*^CCSD(T)^_final_ = Δ*E*^CCSD(T#)-F12a^_cT(D)_ + Δ(DK)^CCSD(T)^,

where the last term is correction for scalar-relativistic effects at the second-order DK level, obtained from conventional CCSD(T) calculations

2 Δ(DK)^CCSD(T)^ = Δ*E*^CCSD(T)^_cT(D)-DK_ − Δ*E*^CCSD(T)^_cT(D)_.

The cT(D)-DK basis set is DK-recontraction of the cT(D). Calculations with the remaining WFT methods were performed using the cT(D)-DK basis set and the resulting energy differences were corrected to the CBS limit of each method based on the observed^71^ excellent transferability of the basis set incompleteness error between CCSD(T) and other WFT methods, *i.e.*,

3 Δ*E*^method^_final_ = Δ*E*^method^_cT(D)-DK_ + Δ*E*^CCSD(T)^_final_ − Δ*E*^CCSD(T)^_cT(D)-DK_.

Full computational details can be found in the ESI.†

#### 2.2.3 Choice of active space in multireference calculations (CASPT2, MRCI)

Based on Pierloot's rules for mononuclear TM complexes,^93,94^ the set of active orbitals was chosen to include: (a) five valence TM 3d orbitals, (b) one or two mostly doubly occupied ligand orbitals considerably overlapping with the TM 3d orbitals to form covalent metal–ligand combinations, and (c) up to five mostly virtual orbitals with the TM 4d character to describe the double-shell effect, in some complexes jointly with π-backdonation (the number of these orbitals was reduced from five down to three in some lower-spin states, in which the corresponding 3d orbitals are nearly empty, for the sake of avoiding uncontrolled orbital rotations). For detailed description of the active orbitals, see Table S6, ESI.† The resulting active space of 10–12 orbitals is regarded as the standard choice for octahedral complexes^52,63,95,96^ as it reasonably accounts for metal–ligand covalency and double-shell effects. A slightly larger active space of 14 active orbitals was chosen for organometallic complex A8 following the work of Pierloot *et al.*^97^ (see Table S7, ESI†).

### 2.3 Vibrational, environmental, and substituent corrections

The vibrational (*δ*_vibr_), environmental (*δ*_env_), and substituent (*δ*_subst_) corrections defined in Section 3.1 were computed using methods and models detailed in Section S3, ESI,† and only briefly mentioned here. The *δ*_vibr_ term was determined from the PBE0-D3(BJ)/def2-TZVP harmonic frequencies (Section S3.1†). The *δ*_env_ term for SCO complexes in solution was determined at the PBE0-D3(BJ)/def2-TZVP level within the COSMO model with the dielectric constant of the actual solvent; in some cases hydrogen-bonded solvent molecules were explicitly added (see Section S3.2.1†). The *δ*_env_ term for SCO complexes in crystals was determined based on periodic, plane-wave PBE+U-D3(BJ) calculations (see Section S3.2.2†). The *δ*_env_ term for vertical excitations was determined at the CASPT2/cT(D)-DK level for a cluster model of the crystal environment (see Section S3.2.3†). The *δ*_subst_ term was determined based on the average of the PBE0-D3(BJ)/def2-TZVP and PBE-D3(BJ)/def2-TZVP results (Section S3.3†).

### 2.4 Experimental procedures

#### 2.4.1 Diffuse reflectance spectra evidencing spin-forbidden d–d transitions

Diffuse reflectance spectra were measured in slow mode on a Shimadzu UV-3600 UV-VIS-NIR spectrophotometer equipped with ISR-260 integrating sphere attachment. The BaSO_4_ (Shimadzu, spectroscopic grade) was used as the reference. Samples were prepared by mixing a crystalline compound with a small amount of BaSO_4_ and grated in an agate mortar. Gaussian analysis of the spectra was performed to locate the maxima of overlapping bands (see the ESI†). Fe(acac)_3_, Co(acac)_3_ and K_3_[Fe(ox)_3_]·3H_2_O were synthesized as described in the literature and recrystallized twice prior to use. K_4_[Fe(CN)_6_]·3H_2_O (p.a.) was from Aldrich.

#### 2.4.2 Synthesis and crystal structure of [Mn(en)_3_]Cl_2_·H_2_O, (1)

Ethylenediamine (en), Sigma-Aldrich, p.a., was kept with solid NaOH for one week under argon and then distilled under argon prior to use. 0.1 g (0.51 mM) of MnCl_2_·4H_2_O was placed in a glass vial and 3 mL of freshy distilled en was added under argon. The vial was sealed with a torch and kept at *ca.* 90 °C (water bath) for *ca.* one month. The vial was then cooled to room temperature and the formed colorless crystals were taken off for X-ray crystal structure analysis and reflectance spectra measurements. The crystals for the X-ray analysis were covered with apiezon to avoid decomposition, while for the reflectance spectra the crystals were dried with filter paper prior to the measurements. The rest of the crystals were filtered off, washed with water and a small amount of MeOH. All manipulations were performed under argon. Anal. calcd for 1·0.5MeOH·1.5H_2_O: C, 21.26; N, 22.89; H, 8.51%. Found: C, 21.36; N, 23.23; H, 8.085%. The X-ray crystal structure analysis was performed at 250 K using the MoKα radiation, with full details described in the ESI.† CCDC 2259710 contains additional crystallographic data.

## 3 Results and discussion

### 3.1 Benchmark set of spin-state energetics (SSE17)

The presently reported SSE17 benchmark set of spin-state energetics is derived from experimental data of 17 complexes (A1**–**A9, B1**–**B4, C1**–**C4), whose structures are shown in Fig. 1. Following the general idea introduced in our previous studies,^11,63,64^ we derive the reference value of the adiabatic spin-state splitting (Δ*E*_ad_) for each SCO complex (A1**–**A9) from the experimental enthalpy difference (Δ*H*), whereas for each of the remaining complexes (B1**–**B4, C1**–**C4) we derive the reference values of the vertical spin-state splitting (Δ*E*_ve_) from the experimental energy of the lowest, singly spin-forbidden d–d absorption maximum (Δ*E*_max_). In both cases, the raw experimental value (Δ*E*_exptl_, *i.e.*, either Δ*H* or Δ*E*_max_) is back-corrected for relevant vibrational (*δ*_vibr_) and environmental (*δ*_env_) effects in order to provide the reference value of the corresponding, purely electronic energy difference (Δ*E*_ref_, *i.e.*, either Δ*E*_ad_ or Δ*E*_ve_):

4 Δ*E*_ref_ = Δ*E*_exptl_ − *δ*_vibr_ − *δ*_env_ − *δ*_subst_.

In addition, for A6 and A7, which are simplified models of the actual complexes studied experimentally (A6′, A7′), we also back-correct for the effect of the ligand's side substituents (*δ*_subst_) present in the actual complex, but simplified to H atoms in the model; for other complexes the *δ*_subst_ term is zero by definition. Below, we discuss the experimental data and applied corrections (*δ*_vibr_, *δ*_env_, *δ*_subst_) leading to determination of the SSE17 dataset, which is summarized in Table 1. Full details of calculating the *δ*-corrections are given in Section S3, ESI.†

**Table 1:** The SSE17 benchmark set: experimental data, applied corrections, and reference values of electronic energy differences^a^

	Complex^b^^,^^c^	Type^d^	Environ.^e^	Δ*E*_exptl_^f^	*δ*_env_	*δ*_vibr_	*δ*_subst_	Δ*E*_ref_
A1	^2,6^[Fe^III^(acac_2_trien)]^+^	ad	CH_2_Cl_2_	1.7 (ref. 98)	0.5	−1.2		3.0(7)^g^
			Acetone	2.0 (ref. 98)	0.8	−1.2		
			MeCN	2.4 (ref. 98)	0.8	−1.2		
			MeOH	3.1 (ref. 98)	0.8	−1.2		
			THF	3.4 (ref. 98)	0.9	−1.2		
A2	^1,5^[Fe^II^(HB(pz)_3_)_2_]	ad	CHCl_3_	5.7 (ref. 99)	−0.2	−1.0		6.9
A3	^1,5^[Fe^II^(HB(tz)_3_)_2_]	ad	Crystal^h^	3.8 (ref. 100)	−0.5	−1.0		5.3
A4	^1,5^[Fe^II^(tacn)_2_]^2+^	ad	Water	5.7 (ref. 101)	2.4	−1.6		4.7(5)^g^
			MeCN	5.0 (ref. 102)	1.8	−1.7		
			DMF	5.0 (ref. 102)	2.4	−1.7		
A5	^1,5^[Fe^II^(1-bpp)_2_]^2+^	ad	Crystal^i^	4.1 (ref. 103)	−0.4	−1.1		5.2(4)^g^
			Acetone	5.8 (ref. 104)	2.0	−1.1		
A6	^1,5^[Fe^II^(tpp)(CN)]^−^	ad	Crystal^j^	3.2 (ref. 105)	0.0	−0.8	−0.1	4.8
A7	^2,4^[Co^II^(ipimpy)_2_]^2+^	ad	Crystal^k^	2.4 (ref. 106)	1.3	−1.0	−0.9	3.0(1)
			Acetone	2.4 (ref. 106)	1.1	−0.8	−0.9	
A8	^1,3^[Ni^II^(acac)(Cp*)]	ad	Toluene	2.7 (ref. 107)	−0.2	−0.3		3.2
A9	^2,6^[MnCp_2_]	ad	Toluene	3.1 (ref. 108)	0.2	−1.3		4.2
B1	^2,4^[Fe^III^(CN)_6_]^3−^	ve	Crystal^l^	58.0^t^^,^^109^	−0.4	−2.3		60.7
B2	^1,3^[Fe^II^(CN)_6_]^4−^	ve	Crystal^m^	68.0^t^	−3.5	−2.9		74.5
B3	^1,3^[Co(en)_3_]^3+^	ve	Crystal^n^	39.5^t^	−0.6	−2.1		42.1
B4	^1,3^[Co(acac)_3_]	ve	Crystal^o^	26.0^t^	1.5	−1.8		26.4
C1	^4,6^[Fe(acac)_3_]	ve	Crystal^p^	−27.4^t^	1.9	−0.2		−29.1
C2	^4,6^[Fe(ox)_3_]^3−^	ve	Crystal^q^	−30.3^t^	2.2	−0.2		−32.3
C3	^4,6^[Mn(en)_3_]^2+^	ve	Crystal^r^	−45.2^t^	0.0	−1.6		−43.5
C4	^3,5^[Fe(H_2_O)_6_]^2+^	ve	Crystal^s^	−37.2^t^	1.0	−0.2		−38.0

a All values in kcal mol^−1^.

b Superscript gives multiplicities of the considered spin states.

c For ligand abbreviations see the caption of Fig. 1.

d Type of energy difference: adiabatic (ad) or vertical (ve).

e Molecular environment, *i.e.* solvent or crystal, in which experimental data were obtained.

f Raw experimental value: enthalpy difference Δ*H* for adiabatic energies of complexes A1**–**A9 or energy corresponding to band maximum position Δ*E*_max_ for vertical spin-forbidden transitions in complexes B1**–**B4, C1**–**C4, with reference to the source of data.

g For complexes characterized in multiple environments, the assumed reference value is the mean of different back-corrected values, the uncertainty estimate is based on the maximum deviation of the back-corrected values from the mean.

h [Fe(HB(tz)_3_)_2_], refcode BAXFIS[01].^100^

i [Fe(1-bpp)_2_](BF_4_), refcode XENBEX03.^103^

j [K(222)][Fe(tpp)(CN)], refcode QOVKIW[03].^110^

k [Co(ipimpy_2_)(ClO_4_)_2_], refcode IQICEQ.^111^

l K_3_[Fe(CN)_6_], ICSD 60535.^112^

m K_4_[Fe(CN)_6_]·3H_2_O, refcode XUNNAX.^113^

n [Co(en)_3_]Cl_3_, refcode IRIRAC.^114^

o [Co(acac)_3_], refcode COACAC03.^115^

p [Fe(acac)_3_], refcode FEACAC05.^116^

q K_3_[Fe(ox)_3_]·3H_2_O, refcode KALGOU.^117^

r [Mn(en)_3_]Cl_3_·H_2_O, CCDC 2259710 (this work).

s [Fe(H_2_O)_6_](NH_4_)_2_(SO_4_)_2_, ICSD 14346.^118^

t This work.

Note that all energy differences between spin states are consistently defined under the following sign convention (which also applies to the *δ*-corrections):

5 Δ*E* = *E*(higher-spin) − *E*(lower-spin).

Thus, Δ*E* < 0 for complexes with HS ground state (C1**–**C4).

#### 3.1.1 SCO complexes (A1**–**A9)

The reference experimental value is the molar enthalpy of the SCO process (Δ*H*), which we use to derive the adiabatic electronic energy difference between the involved spin states (Δ*E*_ad_). All the experimental Δ*H* values were taken from the literature (see references in Table 1). These values originate either from calorimetric measurements (for A3 and A5 in the crystal) or thermodynamic analysis of temperature-dependent spin equilibria (*e.g.*, fitting magnetic susceptibility or magnetic resonance data as a function of temperature). Note that for all considered SCO complexes, the observed transitions are single-step and without hysteresis, making it straightforward to relate the observed Δ*H* to the underlying Δ*E*_ad_ of the spin-transiting molecule.

The vibrational correction (*δ*_vibr_) needed to relate the Δ*H* and Δ*E*_ad_ values accounts for the difference in zero-point energies (ZPEs) and thermal vibrational energies between the two spin states.^11^ It was computed based on DFT frequencies using a well-known expression from statistical thermodynamics (see Section S3.1 and eqn (S.8), ESI†), which is based on the harmonic oscillator model. The *δ*_vibr_ corrections are within 2 kcal mol^−1^ in magnitude and uniformly negative (*cf.*Table 1) due to the lowering of metal–ligand vibrational frequencies upon the LS → HS transition.^119,120^

The environmental correction (*δ*_env_) describes the influence of the environment (solution or crystal) on the Δ*E*_ad_ value. This correction was computed depending on the experimental conditions under which a given SCO complex has been characterized. For complexes characterized in solution (A1, A2, A4, A5, A7**–**A9), the *δ*_env_ correction was determined using COSMO/DFT calculations with the dielectric constant corresponding to the actual solvent used in the experiment. In addition, when considering complexes (A1 and A4) that contain solvent exposed N–H groups, which are potential H-bond donors, in solvents that are potential H-bond acceptors (acetone, MeCN, MeOH, THF, DMF, water), we added explicit solvent molecules to attain a more realistic description (for details, see Section S3.2.1, ESI†). As might be expected, the *δ*_env_ corrections are negligible in non-polar solvents such as toluene, but become more important in polar solvents, especially when H-bonding is operative. For SCO complexes characterized in the solid state (A3, A5**–**A7), the *δ*_env_ correction was determined from periodic DFT+U calculations using a methodology similar to that recently described by Vela with co-workers,^67^ which is detailed in Section S3.2.2, ESI.† The *δ*_env_ corrections due to crystal packing are within 1.5 kcal mol^−1^, sometimes negligible (A6). However, the present sample of solid-state SCO complexes is too small to draw general conclusions about the role of crystal packing effects, which are known to be much larger in certain cases.^11,121^ Also note that the present definition of the *δ*_env_ term is slightly different from that of Vela *et al.*,^67^ who assumed for isolated complexes geometries excised from respective crystal models, whereas in the present definition these are the COSMO/PBE0-D3(BJ)/def2-TZVP geometries, identical with those used in subsequent single-point WFT and DFT calculations.

The substituent correction (*δ*_subst_) for complexes A6 and A7 was quantified using dispersion-corrected DFT calculations (Section S3.3, ESI†). A negligible *δ*_subst_ value is obtained for A6 showing that Ph side substituents of the porphyrin ring present in A6′, but replaced with H atoms in A6, have almost no effect on the singlet–quintet splitting. This is similar to the previous case of triplet–quintet splitting in [Fe^II^(tpp)].^121^ Note, however, that larger substituent effects have been observed in other metalloporphyrins.^121^ Moreover, the ligand's substituents may indirectly influence spin-state energetics through the crystal packing effect (which is obviously included in the *δ*_env_ correction, calculated here with full ligand representation). In the case of A7, the *δ*_subst_ correction (due to simplification of the iPr groups in A7′ to CH_3_ groups in A7) is *ca.* 1 kcal mol^−1^.

#### 3.1.2 Non-SCO complexes (B1**–**B4, C1**–**C4)

The reference experimental value is the position of the absorption maximum of a spin-forbidden d–d transition, translated to energy units

6 Δ*E*_max_ = ±*hcN*_A_*

<svg xmlns="http://www.w3.org/2000/svg" version="1.0" width="13.454545pt" height="16.000000pt" viewBox="0 0 13.454545 16.000000" preserveAspectRatio="xMidYMid meet"><metadata>
Created by potrace 1.16, written by Peter Selinger 2001-2019
</metadata><g transform="translate(1.000000,15.000000) scale(0.015909,-0.015909)" fill="currentColor" stroke="none"><path d="M160 840 l0 -40 -40 0 -40 0 0 -40 0 -40 40 0 40 0 0 40 0 40 80 0 80 0 0 -40 0 -40 80 0 80 0 0 40 0 40 40 0 40 0 0 40 0 40 -40 0 -40 0 0 -40 0 -40 -80 0 -80 0 0 40 0 40 -80 0 -80 0 0 -40z M80 520 l0 -40 40 0 40 0 0 -40 0 -40 40 0 40 0 0 -200 0 -200 80 0 80 0 0 40 0 40 40 0 40 0 0 40 0 40 40 0 40 0 0 80 0 80 40 0 40 0 0 80 0 80 -40 0 -40 0 0 40 0 40 -40 0 -40 0 0 -80 0 -80 40 0 40 0 0 -40 0 -40 -40 0 -40 0 0 -40 0 -40 -40 0 -40 0 0 -80 0 -80 -40 0 -40 0 0 200 0 200 -40 0 -40 0 0 40 0 40 -80 0 -80 0 0 -40z"/></g></svg>

*_max_,

where **_max_ is the wave number at the band maximum position, *h* is the Plack constant, *c* the velocity of light, and *N*_A_ the Avogadro constant. The sign ± is chosen for complexes with LS or HS ground state, respectively, due to the sign convention (5). We use the Δ*E*_max_ values obtained from experimental spectra (more of which is explained later) to derive vertical energy differences (Δ*E*_ve_) between the pairs of involved spin states. Note that for the purpose of developing the SSE17 dataset, we are only interested in the lowest-energy, singly spin-forbidden d–d transitions, *i.e.*, doublet–quartet for the LS d^5^ complex B1; singlet–triplet for LS d^6^ complexes B2–B4; sextet–quartet for HS d^5^ complexes C1–C3; and quintet–triplet for the HS d^6^ complex C4. The corresponding bands are straightforward to assign based on Tanabe–Sugano diagrams^15,122,123^ (see Fig. S9, ESI†).

The vibrational correction (*δ*_vibr_) accounts for the difference between the position of the absorption maximum and the underlying vertical excitation energy, *i.e.*, deviation from the vertical energy approximation.^64,124,125^ The *δ*_vibr_ term was quantified from simulations of the vibrational progression of the d–d transition within the Franck–Condon approximation, following the approach introduced in our previous work^64^ and detailed in Section S3.1.2, ESI.† As can be seen from Table 1, the resulting vibronic corrections to vertical energies are uniformly negative (under the sign convention (5)) and their magnitudes range from negligible for some HS complex up to 2–3 kcal mol^−1^ in the case of LS complexes. These *δ*_vibr_ corrections have good correlation with the ZPE differences between the spin states (Table S8, ESI†), suggesting^11,125^ that the main physical effect responsible for deviation from the vertical energy approximation is the change of vibrational frequencies upon the spin transition.

The environmental correction (*δ*_env_) describes the effect exerted on the Δ*E*_ve_ value by the molecular environment in which the optical spin-transition is measured. Being aware from previous studies^11,34,64,126^ that d–d vertical excitation energies are very sensitive to assumed molecular geometries, and that the latter ones are difficult to computationally predict with sufficient accuracy (especially for TM complexes in solution), we decided to include in the SSE17 benchmark set only complexes for which the d–d bands have been characterized for solid-state compounds with known crystal structures. The availability of the crystal structure evidences not only the geometry of light-absorbing TM complex, but also its molecular environment in the second coordination sphere, both of which may influence the vertical excitation energy. Both types of structural information are also not easily available for TM complexes in solution, which is why we intentionally do not consider any solution-state data of d–d transitions in the construction of the SSE17 benchmark. The use of arbitrary computed geometries without a proper backup from the experimental crystal structures could easily lead to significant and uncontrollable errors in calculated vertical energies, which is precisely what we would like to avoid in developing the benchmark set.

To determine the *δ*_env_ correction for a spin-excitation in the solid state, a cluster model of each light-absorbing TM complex was constructed based on the experimental crystal structure of the actual compound used in the measurements (see footnotes under Table 1 for references). The cluster model was composed of a single TM complex surrounded by its neighboring counterions (treated quantum-mechanically), whereas the interaction with the remaining ions present in the crystal lattice was described by the Ewald potential (electrostatic embedding).^127^ For non-ionic complexes B4 and C1, the cluster model was limited to a single TM complex in its crystalline geometry. Details of the cluster models can be found in Section S3.2.3, ESI.† The environmental correction *δ*_env_ was obtained as the difference between two vertical excitation energies calculated at the CASPT2 level: one for the cluster model, another for the isolated TM complex in vacuum using its COSMO/PBE0-D3(BJ) geometry, *i.e.*, the same one as adopted in subsequent single-point WFT and DFT calculations. Such definition of the *δ*_env_ term (a) utilizes geometry information from the experimental crystal structure and (b) ensures consistency between the geometry adopted in the single-point calculations and the reference value (resulting from subtraction of the *δ*_env_ term from the experimental band maximum position), and thus effectively (c) eliminates the above mentioned problem with the sensitivity of the vertical energy to the choice of geometry.

In our approach we choose COSMO, rather than vacuum geometries, as the former ones are usually closer to crystalline geometries,^11,72,80,119^ and thus typically lead to smaller *δ*_env_ corrections. For example, in the case of B3 considered before,^11^ the *δ*_env_ correction to the singlet–triplet vertical excitation energy is only −0.6 kcal mol^−1^ with respect to the COSMO geometry (present choice), but would be −4.2 kcal mol^−1^ for the vacuum geometry. The effect is even more pronounced for [Fe(CN)_6_]^4−^(B2), in which the *δ*_env_ correction for the singlet–triplet vertical excitation energy would be greater than 20 kcal mol^−1^ with respect to the vacuum geometry, to be compared with only −3.5 kcal mol^−1^ with respect to the COSMO geometry (Table S11, ESI†). The difference is related mainly to the Fe–C distance being much longer in vacuum (1.986 Å) than in the crystal (1.918 Å) or COSMO model (1.912 Å). Similar differences between the gaseous and crystalline geometries of TM cyanides were noticed by Hocking *et al.*^128^ Interestingly, even in the case of K_4_[Fe(CN)_6_]·3H_2_O where strong CN^−^⋯K^+^ bonding interactions^129^ are present in the crystal structure (and in our cluster model), it is mainly the geometry of the inner [Fe(CN)_6_]^4−^ that determines the *δ*_env_ correction; the interactions with added K^+^ cations and the rest of ionic lattice contribute only 0.5 kcal mol^−1^ (*cf.* Table S11†).

As mentioned above, all the experimental data of complexes B1**–**B4 and C1**–**C4 were obtained for solid-state compounds with known crystal structures (see references below Table 1) and diffuse reflectance spectroscopy was used to record their spin-forbidden d–d transitions in the solid state. The reflectance spectra of complexes B1**–**B4 and C1**–**C4 are provided in Fig. S1–S8, ESI.† These are new experimental data with the exception of K_3_[Fe(CN)_6_] (containing B1), for which we used a good quality reflectance spectrum available in the literature.^109^ For K_3_[Fe(ox)_3_]·3H_2_O (containing C2), the presently obtained spectrum is similar as given by Jørgensen^15^ (Fig. 8† therein), although his spectrum was provided in a very small size and without sufficient details, making it necessary to record the new one. The spin-forbidden bands of our interest are usually well resolved in these reflectance spectra, giving separate low-intensity maxima. Only in three cases (B1, B2, C4) they are overlapped on more intense spin-allowed bands, making it necessary to perform the Gaussian analysis to assign the maximum position.

#### 3.1.3 Discussion of the benchmark set

Approximately one-half of the SSE17 set are SCO complexes with the energy differences (Δ*E*_ad_ values) from 3 to 7 kcal mol^−1^. The rest of the SSE17 benchmark set is evenly divided into LS (B1**–**B4) or HS (C1**–**C4) non-SCO complexes, for which the reference spin-state splittings (Δ*E*_ve_ values) are much greater in magnitude. Due to the diversity of TMs, ligand-field strengths, and coordination architecture, the present SSE17 set is a significant step beyond the previous similar attempts from our group, which were limited to four Fe octahedral complexes^63^ or metallocenes.^64^

Compared with the set of octahedral complexes studied in ref. 63, we now treat the vibrational and environmental corrections more consistently. We also decided to exclude two of the previously studied complexes in view of some controversies associated with them. The first of these complexes, [Fe(H_2_O)_6_]^3+^, is presently excluded in view of recurring suggestions^53*a*^ that its sextet–quartet band could originate from a hydrolysis product. (An in-depth analysis of [Fe(H_2_O)_6_]^3+^, which disproves these suggestions, will be published separately.) The second complex, [Fe(en)_3_]^3+^, is excluded due to the lack of an experimental crystal structure of a compound in which the doublet–quartet absorption band described in the literature^130^ could be conclusively observed to fullfil the requirements of our present methodology. (The previous analysis in ref. 63 was based on the computed crystal structure of [Fe(en)_3_]Cl_3_, which was based on the assumption^130^ that it is isomorphic to [Cr(en)_3_]Cl_3_. Despite undertaken efforts, we are unable, so far, to resolve the crystal structure of the tentative [Fe(en)_3_]Cl_3_.) The two removed complexes are replaced in the SSE17 set by other HS Fe^III^ (C1, C2) or LS Fe^III^ (B1) complexes, showing analogous spin-forbidden transitions.

We found it challenging to meet the requirement of having simultaneously a reflectance spectrum and a crystal structure of a compound containing C3, which epitomizes the important class of HS Mn^II^N_6_ complexes. These complexes tend to be unstable towards oxidation and hence are difficult to handle in synthesis and measurements, possibly explaining the scarcity of appropriate data in the literature. Although Jørgensen^131^ reported C3 in solution (stabilized with hydrazine) already in 1969, no crystals were obtained. In 2017, Manke with co-workers^132^ characterized the crystal structure of [Mn(en)_3_](OAc)_2_, whereas Ren with co-workers,^133^ who used KI to stabilize a Mn^II^ complex, obtained crystalline [Mn(en)_3_]I_2_. We have modified the latter method to synthesize the chloride salt of C3, [Mn(en)_3_]Cl_2_·H_2_O (1), for which we now provide both the reflectance spectrum (Fig. S7†) and the crystal structure (CCDC 2259710, ESI†).

An important element of the SSE17 benchmark set is environmental (*δ*_env_) and vibrational (*δ*_vibr_) corrections. As can be seen from Table 1, both types of corrections can reach up to 3 kcal mol^−1^ in magnitude. The vibrational corrections are uniformly negative (under the sign convention (5)), which is due to the lowering of the vibrational frequencies upon transition from the lower-spin to the higher-spin state. The environmental corrections vary for different systems and can be both positive or negative. In some cases one of these corrections is negligible or the two corrections, taken together, tend to cancel out, but neither of these holds true in general. Thus, *δ*_env_ and *δ*_vibr_ corrections are generally important and it seems that neither of them is possible to predict (or neglect) in advance without performing the appropriate calculations. For vibronic corrections of non-SCO complexes the approximation *δ*_env_ ≈ 0.9 × ΔZPE holds to within 0.9 kcal mol^−1^ (*cf.* Table S8†), which may be useful as a rough estimate in future studies.

It should be stressed as a side remark that the *δ*_env_ corrections used in this work are defined with respect to the COSMO/PBE0-D3(BJ) geometries, the same ones as used in subsequent single-point WFT and DFT calculations. The use of COSMO geometries is different from the previous work^63^ where vacuum geometries where used. The difference is of limited importance for adiabatic energies in SCO complexes, but potentially very important for vertical energies^11^ (see also examples above). In any case, the present benchmark set is valid only for single-point calculations on top of the provided (COSMO/PBE0-D3(BJ)) geometries. Any modification of these geometries would require re-determination of the reference values by recomputing the *δ*_env_ corrections.

Of particular attention are SCO complexes characterized simultaneously in different environments: both in solution and in the crystal (A5, A7) or in several solvents (A1, A4). In such cases, the energy differences back-corrected from different environments are slightly different, reflecting limited accuracy of the models and methods used to quantify the *δ*_env_ term. We use the mean of the back-corrected values to provide the most objective reference value, whereas deviations of individual back-corrected values from the mean provide a rough measure of the uncertainty due to imperfect description of the environmental effects. In the case of A5 (which was already discussed in the recent perspective^11^), the reference values back-corrected from acetone solution and BF_4_^−^ salt are in a relatively good mutual agreement, corresponding to the mean value of 5.2 kcal mol^−1^ with only 0.4 kcal mol^−1^ deviations of the individual values from the mean. An even better agreement is observed in the case of A7, for which the energies back-corrected from the crystal and solution are identical to within 0.1 kcal mol^−1^. In the case of A1, the energies back-corrected from different solvents span the range of 2.3–3.6 kcal mol^−1^. The observed spread shows that variation of the experimental Δ*H* value with solvent is not perfectly paralleled by the calculations. Still, however, these data allow estimation of the reference energy difference for A1 as the mean value of 3.0 kcal mol^−1^ with maximum deviation of 0.7 kcal mol^−1^. In the case of A4, the values back-corrected from different solvents fall between 4.3 and 4.9 kcal mol^−1^ (mean 4.7 kcal mol^−1^), which is again a good mutual agreement. It is obviously not possible to apply similar procedures in all cases (due to the lack of experimental data in different environments), but these examples suggest that uncertainties associated with estimation of the *δ*_env_ term are likely within 1 kcal mol^−1^.

Other sources of error in our reference values are related to the *δ*_vibr_ correction, the *δ*_subst_ correction (for A6 and A7) and uncertainties of the experimental data (*e.g.*, from the fitting procedure used to determine the Δ*H* value; associated with reading the position of the maximum for a weak d–d band, especially when Gaussian analysis has to be used to resolve overlapping bands). Overall, our tentative, but conservative estimate of possible errors in the reference values is 1–3 kcal mol^−1^. This also accounts for sensitivity of the *δ*-corrections to the choice of method or computational parameters (see Section S3.4, ESI†). The above estimate of the error bars of 1–3 kcal mol^−1^ means that errors of 1 kcal mol^−1^ are likely, whereas errors beyond 3 kcal mol^−1^ are increasingly unlikely. The SSE17 reference data are thus certainly not appropriate to discuss individual deviations in a sub-kcal mol^−1^ range. However, anticipating the results discussed below, many of the calculated spin-state splittings show much larger deviations, which can be hardly blamed on uncertainties of the reference data.

### 3.2 Performance of quantum chemistry methods

**Fig. 2:**
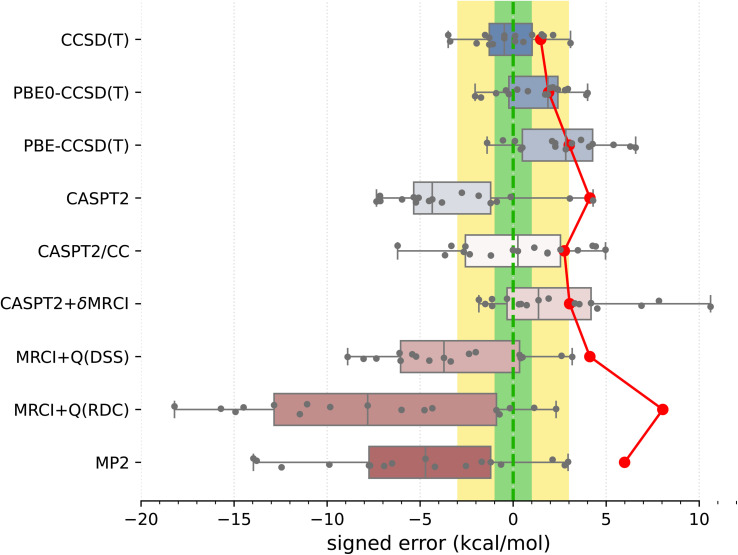
Distribution of errors in the SSE17 spin-state splittings calculated using selected WFT methods (box-plot) and the resulting MAE of each method (point-plot). Each box represents 50% of the population (with the median marked in the middle) and the whiskers extend from the minimum to the maximum of the population. Individual data are shown as points. To guide the eye, error ranges ±1 kcal mol^−1^ (“chemical accuracy”) and ±3 kcal mol^−1^ (“TM chemical accuracy”) are colored in green and yellow, respectively.

**Fig. 3:**
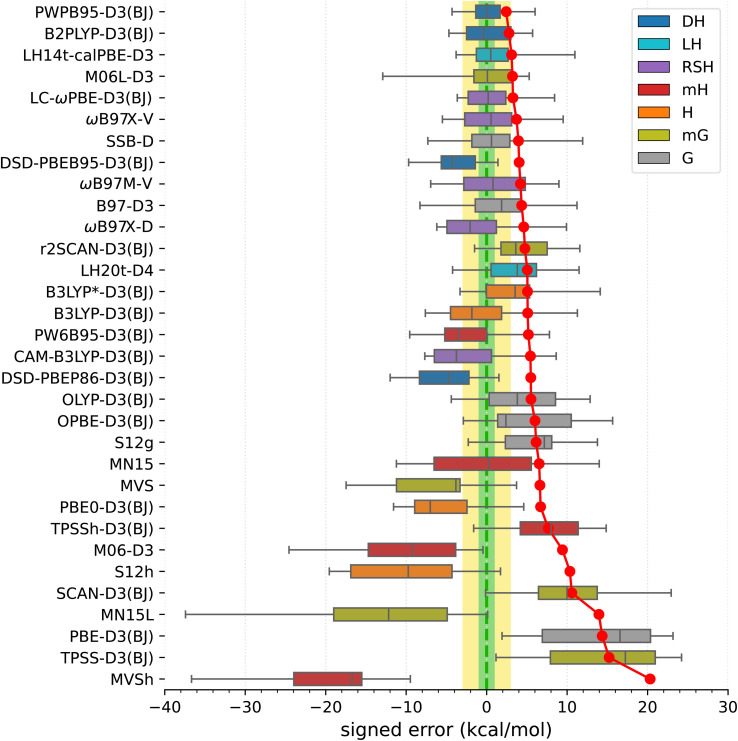
Distribution of errors in the SSE17 spin-state splittings calculated using selected DFT methods (box-plot) and the resulting MAE of each method (point-plot). The boxes are colored by functional type: gradient (G), meta-gradient (mG), hybrid (H), meta-hybrid (mH), range-separated hybrid (RSH), local hybrid (LH), double-hybrid (DH).

Armed with the present SSE17 benchmark, we are now able to quantify the accuracy of spin-state energetics predicted by various quantum chemistry methods. To this end, Fig. 2 and 3 show the distributions of errors in the SSE17 spin-state splittings calculated using selected WFT and DFT methods, respectively. The signed errors being analyzed are deviations of the calculated values from the corresponding reference values (from Table 1). The distribution of errors is presented is the form of a box-plot, whereas the mean absolute error (MAE) of each method is shown as the point-plot. Numerical data for individual complexes and additional error statistics can be found in the ESI (Tables S17 and S18).†

#### 3.2.1 WFT methods

We have benchmarked several WFT methods that were previously recommended for computation of spin-state energetics: CCSD(T) with HF reference orbitals and KS-CCSD(T) with either PBE or PBE0 reference orbitals (*i.e.*, PBE-CCSD(T), PBE0-CCSD(T)), CASPT2, CASPT2/CC,^52^ CASPT2+δMRCI,^53^ and MRCI+Q (using CW internally contracted formulation^87^). We also included MP2 for comparison. Note that all WFT methods were applied without any local-correlation approximations and their results are approximate CBS limits (see Computational and experimental methods).

It is clear from Fig. 2 than none of the benchmarked WFT methods can perfectly reproduce the reference data (which also have intrinsic errors, possibly 1–3 kcal mol^−1^, as was discussed above). However, the CCSD(T) method based on HF orbitals is able to reproduce the reference data most accurately, with the MAE of only 1.5 kcal mol^−1^, the RMSD (root mean square deviation) of 1.8 kcal mol^−1^ and the maximum error of −3.5 kcal mol^−1^. The inspection of CCSD(T) results for individual complexes (Table S17†) reveals that the largest negative errors, indicative of the higher-spin state being overstabilized, are observed for Fe^III^ complexes A1 and B1. The largest positive error, indicative of the lower-spin state being overstabilized, is observed for the Co^III^ complex B4. The occurrences of positive and negative errors are well balanced across the SSE17 set, resulting in the mean and median errors within −0.5 kcal mol^−1^. Thus, the CCSD(T) method appears to be (on average) not significantly biased toward either higher-spin or lower-spin states.

We have investigated whether the observed CCSD(T)'s errors can be correlated with various diagnostics of multireference character commonly used in the literature (including the diagnostics based on the CCSD amplitudes, the triples contribution to differential correlation energy, the weight of the leading configuration in a CASSCF wave function, occupation numbers of the CASSCF natural orbitals, and the effect of varying the exact exchange admixture in DFT); in all cases the answer obtained by us is definitely negative (ESI, Section S4.2†). For all presently investigated complexes, CCSD(T) appears to maintain its relatively high accuracy for relative spin-state energetics, although some of the diagnostic values observed here are rather high compared with the criteria suggested in the literature^134^ (*e.g.*, the *D*_1_ diagnostic is above 0.15 in more than 60% of cases and above 0.20 in four cases; see Table S16†). This corroborates that these diagnostics cannot be used to predict the accuracy achieved in CCSD(T) calculations.^11^

An interesting question, widely discussed in the literature,^45,48,135–138^ is whether switching from HF to KS orbitals in the reference Slater determinant leads to more accurate CCSD(T) energetics. Looking at the present results, we can compare the accuracy of CCSD(T) energetics based on three choices of orbitals: HF, PBE0 (25% exact exchange), and PBE (no exact exchange). For some complexes, the use of PBE0 or PBE orbitals is beneficial to reduce the CCSD(T) errors (*e.g.*, A1, A7), but for other cases the errors increase (*e.g.*, A3**–**A5) or there is almost no effect (*e.g.*, A8). Overall, the MAE and maximum error are slightly greater for PBE-CCSD(T) and PBE0-CCSD(T) than for genuine CCSD(T). Thus, although some improvement may be observed for certain complexes, our data do not support the hypothesis that the use of KS orbitals is systematically better than the use of HF orbitals. (In fact, the opposite is true for the presently studied SSE17 data, although the deterioration of the accuracy is minor.) These observations agree with the conclusions of Benedek *et al.*,^138^ who also observed no systematic improvement in the CC energies of small molecules when switching from HF to KS orbitals.

Note that some of the recent claims advocating the usage of KS orbitals in CCSD(T) calculations^137,139^ were based on the CCSD(T) energies calculated under the DLPNO (domain-based local-pair natural orbitals) approximation. The accuracy of this approximation may depend on the type of reference orbitals and sometimes strongly degrades when HF orbitals are used.^48,71^ This probably explains the strong dependence of spin-state energetics on the type of reference orbitals, which was observed in the DLPNO-CCSD(T) studies, as well as therein claimed significant improvement of the accuracy upon switching from HF to KS orbitals. However, these effects are specific to the DLPNO approximation and are not general features of the CCSD(T) method. In our study, which is based on the canonical CCSD(T) method, *i.e.*, without any local correlation approximations, the effect of switching from HF to KS orbitals is generally smaller than in the DLPNO-based studies (see also discussion in ref. 71).

The relatively high accuracy of the CCSD(T) spin-state energetics has already been noted in our previous benchmark study of four Fe complexes.^63^ In that work, the reduction of the CCSD(T)'s error by 1.6 kcal mol^−1^ by switching from HF to KS orbitals (B3LYP, 20% of exact exchange) was observed for one of the investigated complexes [Fe(tacn)_2_]^2+^, which is identical with the present A4. However, such improvement is no longer observed in the present study, which is due to a combination of reasons. First, the presently determined reference value for A4 is higher by 0.9 kcal mol^−1^ than that determined in ref. 63 due to the usage of different functionals in the determination of the *δ*-corrections and deriving the present reference value by averaging data back-corrected from three solvents. Second, the presently determined CCSD(T) energy is smaller than that in ref. 63, which is mainly caused by the usage of the more reliable^71^ CCSD(T#)-F12a method to determine the CBS limit in the present work. Finally, we realized that in order to properly capture the (T) energy term in KS-CCSD(T) calculations, one should use the open-shell CC program even for closed-shell singlets, which was not the case in ref. 63. If the KS-(T) term is computed properly, like in this study, the KS-CCSD(T) method leads to a larger splitting than the CCSD(T) method (opposite to the behavior observed in ref. 63). Consequently, not only for A4, but also for all other Fe^II^ SCO complexes included in the SSE17 set (A2**–**A6), the CCSD(T) based on HF orbitals yields smaller singlet–quintet gaps than either PBE0-CCSD(T) or PBE-CCSD(T).

Proceeding now to multireference methods, we observe the already known^52,63^ tendency of the CASPT2 method (with the standard choice of active space and the default value of the IPEA shift parameter) to overstabilize higher-spin states, *i.e.*, CASPT2 calculations usually lead to negative errors in the SSE17 benchmark, with the mean signed error of −3.3 kcal mol^−1^, maximum error of −7.3 kcal mol^−1^, and the MAE of 4.1 kcal mol^−1^. The negative errors observed in CASPT2 calculations are reduced by both CASPT2/CC and CASPT2+δMRCI methods. For CASPT2/CC, the median and the mean signed error are very close to zero. For CASPT2+δMRCI, the mean signed error is about 2 kcal mol^−1^. Both of these methods have an MAE of *ca.* 3 kcal mol^−1^. Somewhat surprisingly, however, for organometallic complexes A8 and A9, the genuine CASPT2 method leads to positive errors of 3–4 kcal mol^−1^, which neither CASPT2/CC nor CASPT2+δMRCI can reduce (*cf.* Table S17†). In fact, complex A8 is responsible for the maximum error (nearly +11 kcal mol^−1^) of the CASPT2+δMRCI method. Other considerable outliers for the CASPT2+δMRCI method are complexes A2 and A9, with errors of 7–8 kcal mol^−1^. In the case of CASPT2/CC, the largest error of −6 kcal mol^−1^ is observed for A7.

It has been suggested^53*b*^ that the CASPT2+δMRCI method outperforms CCSD(T) for complexes with significant π-backdonation. However, this conjecture is not confirmed by the SSE17 benchmark, in which A6, B1 and B2 (with cyanide ligands) as well as A8 and A9 (with Cp ligands) are typical complexes featuring π-backdonation. Inspections of the detailed results (Table S17†) reveals that for none of these complexes the CASPT2+δMRCI method is significantly more accurate than CCSD(T). In fact, we observe a slight improvement only for B1 (CCSD(T) error of −3.4 kcal mol^−1^, CASPT2+δMRCI error of −1.8 kcal mol^−1^), but a slight deterioration for B2 (CCSD(T) error of 0.5 kcal mol^−1^, CASPT2+δMRCI error of 4.2 kcal mol^−1^) and a significant deterioration for A8 and A9, for which CASPT2+δMRCI has errors of 10.6 and 7.8 kcal mol^−1^, respectively.

Although CASPT2+δMRCI was originally motivated as a computationally tractable approximation to a more expensive MRCI method,^53*a*^ our data show that it is actually more accurate than the MRCI+Q itself. This is presumably due to the size-consistency problem in a truncated MRCI, which is only partially resolved by adding an approximate size-consistency correction in the MRCI+Q approach. This problem is alleviated in the CASPT2+δMRCI method, where only a small number of active electrons plus 8 electrons on TM 3s3p orbitals undergo the MRCI treatment.^53^ In our MRCI+Q calculations (in which all valence and TM 3s3p electrons were correlated), we compared several size-consistency corrections:^140^ the original Davidson correction (DC), the renormalized DC (RDC), the Davidson–Silver–Siegbahn (DSS) correction, and the Pople correction (PC). Only the DSS and RDC results are presented in Fig. 2, but all can be found in Table S17, ESI.† For the present set of spin-state energetics, the most accurate formulation is MRCI+Q(DSS), which has statistical errors similar to CASPT2, closely followed by the MRCI+Q(PC), whereas MRCI+Q(RDC) and MRCI+Q(DC) lead to much larger errors, which are in fact greater than those of the MP2 method. Inspection of the detailed results (Table S17†) reveals that discrepancies between different size-consistency corrections are more pronounced for larger complexes, *i.e.*, with a greater number of correlated electrons, suggesting these errors are connected with the violation of size-extensivity. The analogous problems of MRCI+Q calculations were also observed in our previous study of four complexes,^63^ and are now fully confirmed for the larger SSE17 set.

#### 3.2.2 DFT methods

We have benchmarked 32 functionals from different rungs of the Jacob's ladder: gradient functionals (PBE, OLYP, OPBE, SSB, S12g, B97), meta-gradient functionals (TPSSh, M06L, MN15L, MVS, SCAN, r2SCAN), global hybrids (PBE0, B3LYP, B3LYP*, S12h) and meta-hybrids (TPSSh, M06, MN15, PW6B95, MVSh), range-separated hybrids (CAM-B3LYP, LC-ωPBE, ωB97X-V/D, ωB97M-V), local-hybrids (LH14t-calPBE, LH20t), and double-hybrids (PWPB95, B2PLYP, DSD-PBEB95, DSD-PBEP86); see Section S2.1 in the ESI† for references. Most functionals were benchmarked with dispersion corrections, usually D3(BJ).^75^

In view of the results shown in Fig. 3 (for corresponding numeric data, see Table S18, ESI†), the best performers are double-hybrid functionals PWPB86-D3(BJ) and B2PLYP-D3(BJ). These two functionals show relatively small MAEs (2.4 and 2.8 kcal mol^−1^, respectively), nearly zero mean signed and median errors, and maximum errors within 6 kcal mol^−1^. The other two tested double-hybrids (DSD-PBEB95/PBEP86-D3(BJ)) perform considerably worse, showing overstabilization of higher-spin states. Some other functionals highly ranked in the SSE17 benchmark are the following: local hybrid LH14t-calPBE-D3(BJ),^141^ range-separated hybrid LC-ωPBE-D3(BJ),^142^ meta-gradient M06L-D3,^143^ range separated meta-hybrid with nonlocal dispersion ωB87X-V,^144^ and gradient functional SSB-D.^145^ All these have MAEs within 4 kcal mol^−1^, and mean signed errors within 2 kcal mol^−1^, but all of them also feature maximum errors of about 8.5 kcal mol^−1^ or greater.

Functionals traditionally recommended for spin states of TM complexes,^29,65,146,147^ such as B3LYP*-D3(BJ) and TPSSh-D3(BJ) hybrids with 10–15% of exact exchange, do not perform well in the SSE17 benchmark. These two functionals have MAE of 5.1 and 7.7 kcal mol^−1^, respectively, and lead to maximum errors of 14–15 kcal mol^−1^. Inspection of numeric results (*cf.* Table S18†) reveals that these maximum errors are due to overstabilization of lower-spin states in HS complexes C1**–**C4, but even if we restrict our attention to SCO complexes A1**–**A8 (or even a narrower class of Fe^II^ SCO complexes A2**–**A6), these two functionals are also by no means optimal. In fact, considering the entire SSE17 set, B3LYP*-D3(BJ) performs only slightly better than the original B3LYP-D3(BJ) (with 25% of the exact exchange). B3LYP*-D3(BJ) is clearly superior for some SCO complexes, providing nearly accurate results for A1, A4, and A9, but it leads to significant errors of 4–5 kcal mol^−1^ for A2, A3, A6 and A7. The inferior performance of B3LYP* and TPSSh functionals, particularly their significant overstabilization of the quartet state with respect to the sextet state in complexes C1**–**C3, agrees with similar problems of these functionals evidenced in a different benchmark SSCIP6, which is based on probing the ability to reproduce correct ground states in the set of crystalline iron-porphyrins.^121^

**Fig. 4:**
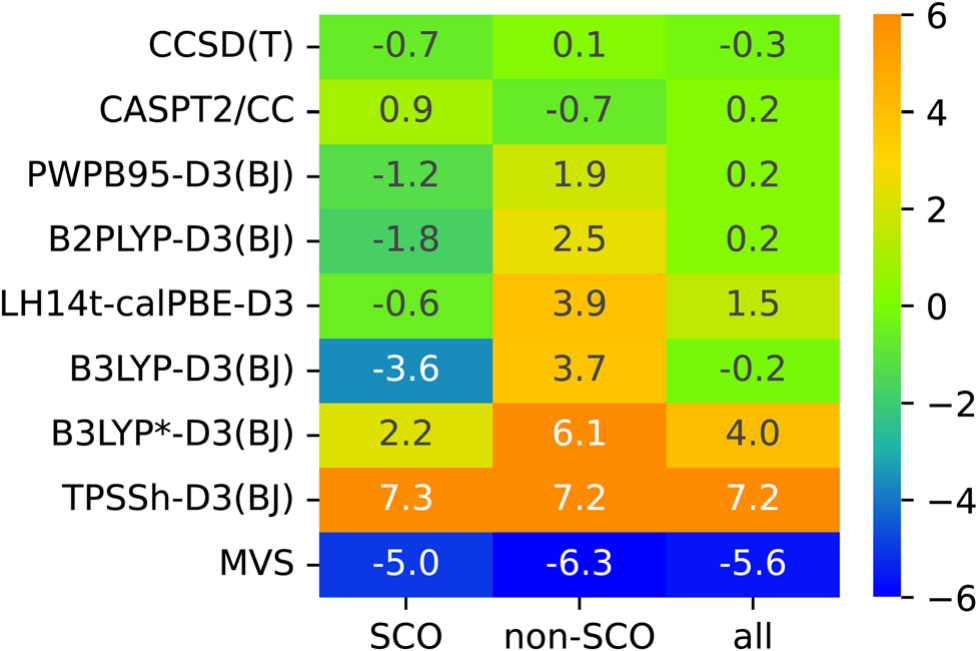
Mean signed errors (kcal mol^−1^) of selected methods for different classes of complexes.

The lack of universality is a problem of many approximate DFT methods. To illustrate this point, Fig. 4 presents mean signed errors of selected methods separately for SCO (A1**–**A9) and non-SCO (B1**–**B4, C1**–**C4) complexes, and for the entire SSE17 set. With CCSD(T) and CASPT2/CC wave-function methods, the errors observed for different classes are comparable. TPSSh-D3(BJ) and MVS are examples of functionals giving rather universally positive or negative errors. By contrast, LH14t-calPBE-D3 is very accurate for SCO complexes, but features significant positive errors for non-SCO complexes. Comparable non-universal behavior is observed for B3LYP-D3(BJ) and B3LYP*-D3(BJ).

For the PWPB95 and TPSSh functionals, we compared the results obtained with the D3(BJ)^75^ and D4 (ref. 148) dispersion corrections (Table S19, ESI†). Obviously, only adiabatic energies (for complexes A1**–**A9) are affected by the dispersion correction term. As shown in Table S19,† individual results vary in some cases by up to 4.4 kcal mol^−1^, but the overall performance of each functional is similar with both choices of the dispersion correction (in fact, slightly worse with the newer D4 one).

## 4 Conclusions

Adhering to the recently recommended strategy of developing benchmark sets for theory in close cooperation with experiment,^149^ we have formulated the novel benchmark set for first-row TM spin-state energetics (SSE17) based on curated experimental data of 17 chemically diverse complexes, classical and organometallic ones, containing various metals and having different ligand-field strengths. The employed experimental data, which are SCO enthalpies or spin-forbidden d–d excitation energies, originate in condensed-phase measurements, but are suitably back-corrected for environmental and vibrational effects to produce reference data directly comparable to electronic energy differences of isolated complexes in vacuum. The presented benchmark set is not only useful for assessing the accuracy of existing quantum chemistry methods, but it is also hoped to be useful for validation of new methods, parameterization of new functionals or developing machine-learning models.

This is the first time that the performance of both WFT and DFT quantum chemistry methods can be quantitatively benchmarked against such an extensive and statistically relevant set of experiment-derived spin-state energetics, and the results obtained here considerably challenge the existing state of knowledge. The most accurate of all tested methods is found to be the single-reference CCSD(T) method, which across the SSE17 set features the mean absolute error (MAE) of 1.5 kcal mol^−1^, the mean signed error of −0.3 kcal mol^−1^, and the maximum error of 3.5 kcal mol^−1^. In contrast to earlier claims in the literature, we have found that the overall accuracy of CCSD(T) spin-state energetics does not systematically improve by using KS orbitals (PBE or PBE0) instead of HF orbitals. Among several multireference approaches that have been benchmarked, the variational MRCI+Q method does not appear to outperform the computationally much cheaper CASPT2; both of them produce MAEs of 4 kcal mol^−1^ and maximum errors of around 7–9 kcal mol^−1^. The form of size-consistency correction is critically important for the accuracy of MRCI+Q. The recently proposed methods CASPT2/CC and CASPT2+δMRCI outperform the original CASPT2 method in terms of typical errors (MAE values of around 3 kcal mol^−1^), but they still lead to considerable maximum errors for some outliers. Neither of the tested multireference methods can consistently outperform the single-reference CCSD(T) method across the SSE17 set. The CCSD(T) maintains its relatively high accuracy despite its single-reference character, and the observed small deviations from the reference values are not correlated with any common diagnostic of multireference character. Clearly, one should not extrapolate the present results to complexes with two or more metals, in which certain spin states may involve antiferromagnetic coupling between different metal sites, not expected to be correctly described using single-reference CCSD(T) calculations. Having such binuclear or polynuclear complexes in mind, in remains an important goal for future studies to find multireference methods that perform well for spin-state energetics.

Among 32 approximate DFT methods that have been benchmarked, the best performers are double-hybrids (PWPB95-D3(BJ) and B2PLYP-D3(BJ)), which due to the MAEs within 3 kcal mol^−1^, the mean signed errors of only 0.2 kcal mol^−1^, and the maximum errors within 6 kcal mol^−1^ appear to be (on average) equally accurate as CASPT2/CC. Our results confirm that the non-universality problem exists in many approximate DFT methods. The functionals traditionally recommended for spin-state energetics, such as TPSSh-D3(BJ) or B3LYP*-D3(BJ), which contain 10–15% of exact exchange, do not perform well across the SSE17 benchmark by yielding the MAEs of 5–7 kcal mol^−1^ and maximum errors beyond 10 kcal mol^−1^. One should be aware of such problems in computational reactivity studies, where these or similar hybrid functionals are still predominantly used. A practical solution for DFT-based reactivity studies is, for example, to add relatively simple corrections based on CCSD(T) spin-state energetics of simplified models.^33^

Although the present benchmark set is comprehensive, it is still mainly focused on Fe complexes, comprising 11 out of 17 items. This slight over-representation of Fe complexes is understandable given their overall importance and abundance of high-quality experimental data. However, it would be beneficial to extend the benchmark set in future studies by including more complexes of Mn, Co and Ni as well as other first-row TMs, depending on the availability of suitable experimental data, analyzed using similar methodology as established here.

## Data availability

The data supporting this article have been included as part of the ESI.† Crystallographic data for [Mn(en)_3_]Cl_2_·H_2_O (1) have been deposited at the CCDC under deposition number 2259710. Additional supporting data (structures and total energies from selected calculations) may be accessed as an ioChem-BD collection under the following link: https://doi.org/10.19061/iochem-bd-7-8.

## Author contributions

M. Radoń: conceptualization, methodology, investigation (computational), data analysis and visualization (lead), funding acquisition, resources (computational), writing — original draft (lead), writing — review & editing. G. Drabik: investigation (computational), data analysis and visualization (computational). M. Hodorowicz: investigation (experimental), data analysis and visualization (crystallographic), writing — original draft (support). J. Szklarzewicz: investigation (experimental), resources (experimental), writing — original draft (support).

## Conflicts of interest

There are no conflicts to declare.

## Supplementary Material

SC-015-D4SC05471G-s001

SC-015-D4SC05471G-s002

SC-015-D4SC05471G-s003

SC-015-D4SC05471G-s004
