# Supplementary material for: Performance of quantum chemistry methods for a benchmark set of spin-state energetics derived from experimental data of 17 transition metal complexes (SSE17)
Source: Chem Sci. 2024 Oct 28;15(48):20189–204. doi: 10.1039/d4sc05471g (PMC11577268; doi:10.1039/d4sc05471g)
Supplement: SC-015-D4SC05471G-s001 [file SC-015-D4SC05471G-s001.pdf]

Supplementary Information (SI) for Chemical Science.
This journal is © The Royal Society of Chemistry 2024

Electronic Supplementary Information (ESI): Performance of Quantum Chemistry Methods for Benchmark Set of Spin–State Energetics Derived from Experimental Data of 17 Transition Metal Complexes (SSE17)

Mariusz Radoń,[*,†] Gabriela Drabik,[‡,†] Maciej Hodorowicz,[†] and Janusz Szklarzewicz[†]

†*Faculty of Chemistry, Jagiellonian University, Kraków, Poland*

‡*Jagiellonian University, Doctoral School of Exact and Natural Sciences, Kraków, Poland*

E-mail: mradon@chemia.uj.edu.pl

Phone: +48 12 6862489

Contents

List of Tables **S-3**

List of Figures **S-4**

S1 Experimental Data **S-6**

S1.1 Reflectance Spectra Evidencing Spin-Forbidden Absorption Bands S-6

S1.2 Crystal structure of $[Mn(en)_3]Cl_2 \cdot H_2O$ S-12

S2 Full Computational Details **S-18**

S2.1 DFT Calculations S-18

S2.2 WFT Calculations S-21

S3 Vibrational, Environmental, and Substituent Corrections **S-27**

S3.1 Vibrational Corrections S-27

S3.1.1 Case 1: Vibrational Enthalpy Corrections to Adiabatic Energies S-27

S3.1.2 Case 2: Vibronic Corrections to Vertical Energies S-28

S3.2 Environmental Corrections S-34

S3.2.1 Case 1a: SCO in Solution S-34

S3.2.2 Case 1b: SCO in Solid State S-38

S3.2.3 Case 2: Spin-Forbidden d–d Transitions in Solid State S-39

S3.3 Substituent Corrections S-46

S3.4 Method-Dependence of δ-Corrections S-47

S4 Numeric Data for WFT and DFT Methods **S-51**

S4.1 Deviations from Reference Values S-51

S4.2 Diagnostics of Nondynamic Correlation S-56

References **S-66**

List of Tables

S1 Crystallographic details for $[Mn(en)_3]Cl_2 \cdot H_2O$ S-13

S2 Bond parameters for $[Mn(en)_3]Cl_2 \cdot H_2O$ S-14

S3 Hydrogen bonds for $[Mn(en)_3]Cl_2 \cdot H_2O$. S-15

S4 Basis Sets cT(D)-DK, cT(D), and Auxiliary Basis Sets for F12 Calculations S-22

S5 Detailed CC Results S-22

S6 Description of Active Spaces in Multiconfigurational Calculations. S-24

S7 CASPT2(12,14) and (14,15) Results for **A8** S-25

S8 Calculated Vibronic Corrections, Differential ZPEs and Relaxation Energies. [a] . . S-29

S9 Solvation Corrections for SCO Complexes in Solution S-37

S10 Crystal Packing Effects for SCO Complexes in Solid State S-39

S11 Singlet–Triplet Excitation Energy of $[Fe(CN)_6]^{4-}$ for Different Models and Geometries S-44

S12 Environmental Corrections for d–d Transitions in Crystals S-45

S13 Substituent Corrections for **A6** and **A7** S-46

S14 Environmental Corrections for SCO in Solution Using PBE0-D3(BJ) and PBE-D3(BJ) S-48

S15 Environmental Corrections for SCO in Crystal Using Different U Values S-49

S16 Environmental Corrections for d–d Transitions Using Different IPEA Values S-50

S17 Deviations of WFT Spin–State Energetic from Reference Values S-52

S18 Deviations of DFT Spin–State Energetic from Reference Values S-53

S19 Comparison of PWPB95 and TPSSh Results with D3(BJ) and D4 Dispersion Corrections S-56

S20 Diagnostics of Nondynamic Correlation S-59

List of Figures

S1 Reflectance spectrum of $K_3[Fe(CN)_6]$ S-7

S2 Reflectance spectrum of $K_4[Fe(CN)_6] \cdot 4\,H_2O$ S-7

S3 Reflectance spectrum of $[Co(en)_3]Cl_3$ S-8

S4 Reflectance spectrum of $[Co(acac)_3]$ S-8

S5 Reflectance spectrum of $[Fe(acac)_3]$ S-9

S6 Reflectance spectrum of $K_3[Fe(ox)_3] \cdot 3\,H_2O$ S-9

S7 Reflectance spectrum of $[Mn(en)_3]Cl_2 \cdot H_2O$ S-10

S8 Reflectance spectrum of $(NH_4)_2Fe(SO_4)_2(H_2O)_6$ S-10

S9 Tanabe–Sugano diagrams for d^5 and d^6 octahedral complexes S-11

S10 Asymmetric unit of $[Mn(en)_3]Cl_2 \cdot H_2O$ S-15

S11 Structure of $[Mn(en)_3]^{2+}$ S-16

S12 Crystal packing for $[Mn(en)_3]Cl_2 \cdot H_2O$ S-17

S13 Natural orbitals of BS solutions for **A8** singlet and **C4** triplet S-20

S14 Vibrational progression of doublet → quartet transition for $[Fe(CN)_6]^{3-}$ S-30

S15 Vibrational progression of singlet → triplet transition for $[Fe(CN)_6]^{4-}$ S-30

S16 Vibrational progression of singlet → triplet transition for $[Co(en)_3]^{3+}$ S-31

S17 Vibrational progression of singlet → triplet transition for $[Co(acac)_3]$ S-31

S18 Vibrational progression of sextet → quartet transition for $[Fe(acac)_3]$ S-32

S19 Vibrational progression of sextet→quartet transition for $[Fe(ox)_3]^{3-}$ S-32

S20 Vibrational progression of sextet → quartet transition for $[Mn(en)_3]^{2+}$ S-33

S21 Vibrational progression of quintet → triplet transition for $[Fe(H_2O)_6]^{2+}$ S-33

S22 Models used in microsolvation calculations for **A1** S-35

S23 Models used in microsolvation calculations for **A4** S-36

S24 Definition of the environmental correction for d–d transition in crystal S-41

S25 Cluster models: $\{K_{14}[Fe(CN)_6]\}^{11+}$ (**B1**) and $\{K_{14}[Fe(CN)_6]\}^{10+}$ (**B2**). S-42

S26 Cluster models: $\{[Co(en)_3]Cl_9\}^{6-}$ (**B3**) and $\{[Mn(en)_3]Cl_6 \cdot 2\,H_2O\}^{4-}$ (**C3**). S-42

S27 Cluster model $\{K_{14}[Fe(ox)_3]\cdot 3\,H_2O\}^{11+}$ (**C2**). S-43
S28 Cluster model $\{[Fe(H_2O)_6](SO_4)_8(NH_4)_{10}\}^{4-}$ (**C4**). S-43
S29 CCSD(T) errors vs T_1 diagnostic S-60
S30 CCSD(T) errors vs D_1 diagnostic S-61
S31 CCSD(T) errors vs D_2 diagnostic S-62
S32 CCSD(T) errors vs M diagnostic S-63
S33 CCSD(T) errors vs %MC diagnostic S-64
S34 CCSD(T) errors vs %DCE and B diagnostics S-65

S1 Experimental Data

S1.1 Reflectance Spectra Evidencing Spin-Forbidden Absorption Bands

The diffuse reflectance spectra evidencing absorption bands due to spin-forbidden d–d transitions are shown in Figures S1–S7. In each case, the absorption intensity is expressed using the standard Kubelka–Munk (KM) function[S1]

$$F_R = \frac{(1-R)^2}{2R}, \tag{S.1}$$

where R is the measured reflectance. Note that F_R is presented in logarithmic scale.

The spectra were resolved into a minimum number of contributing Gaussian bands, whose parameters were fitted using the least-squares Levenberg–Marquardt method as implemented in Fityk v1.3.[S2] In computation of the weighted sum of squared residuals (i.e., the quantity being minimized), the experimental points were weighted by the factor $w = w_1 w_2$, where w_1 takes into account how the constant measurement error in R propagates through the KM function[S1]

$$w_1 = \left(\frac{1+R}{R(1-R)}F_R\right)^{-2}, \tag{S.2}$$

and $w_2 = \lambda^{-2}$ serves to compensate for non-uniform spacing of the experimental points on the energy scale (spectrophometers acquire data uniformly on the wavelength scale). Low-energy noisy regions of the spectra, in which vibrational overtones or combination bands were observed in some cases, were excluded from the Gaussian analysis. (The bands of vibrational origin can be distinguished by their smaller widths and different shape.) Higher-energy regions of the spectrum were included when it was necessary due to band overlapping. The energy range used to perform the Gaussian analysis in each case is indicated in the figure caption. The position of the spin-forbidden absorption band in the experimental spectrum is usually evident even without performing the Gaussian analysis. It only becomes mandatory for **B1** and **B2** ($[Fe(CN)_6]^{3-/4-}$) and **C4** ($[Fe(H_2O)_6]^{2+}$) for which the interesting spin-forbidden band is overlapped on more intense spin-allowed bands.

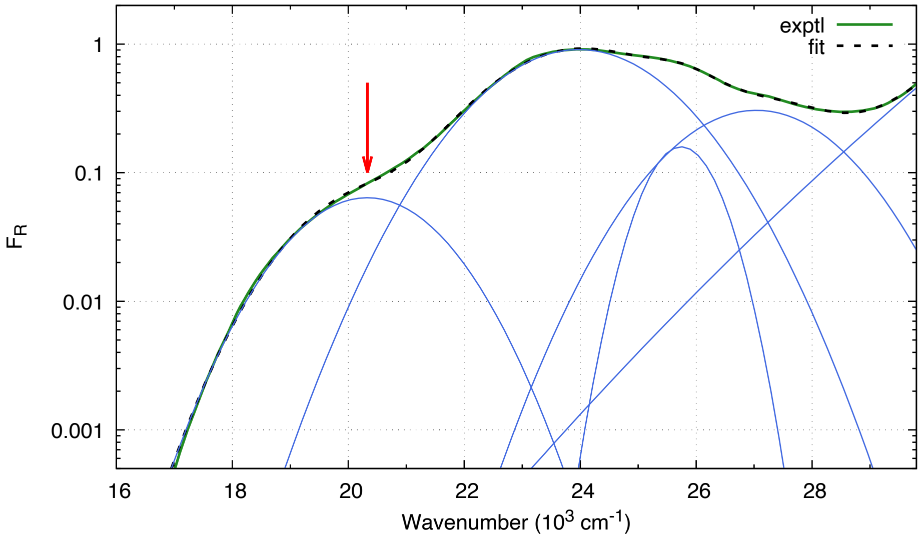

Figure S1: Reflectance spectrum of $K_3[Fe(CN)_6]$ (containing **B1**) digitilised from fig. 1 of ref S3 after KM transformation and its Gaussian analysis in the 16.3–29.8 $\times 10^3$ cm^{-1} range. The arrow shows band maximum position of the lowest d–d transition ($^2T_{2g} \rightarrow {}^4T_{1g}$, $\tilde{\nu}_{max} = 20.3 \times 10^3$ cm^{-1}).

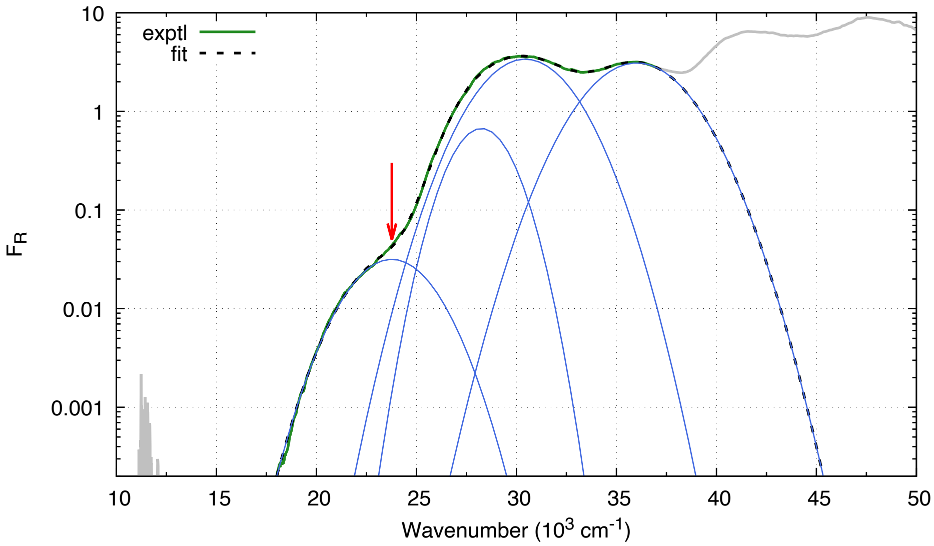

Figure S2: Reflectance spectrum of $K_4[Fe(CN)_6] \cdot 4\,H_2O$ (containing **B2**) after KM transformation and its Gaussian analysis in the 18–37 $\times 10^3$ cm^{-1} range. The arrow shows band maximum position of the lowest d–d transition ($^1A_{1g} \rightarrow {}^3T_{1g}$, $\tilde{\nu}_{max} = 23.8 \times 10^3$ cm^{-1}).

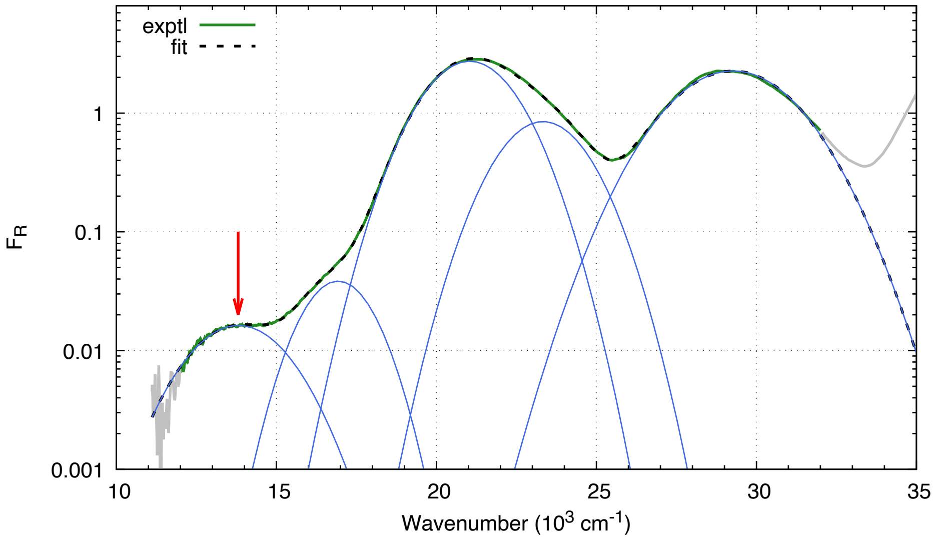

Figure S3: Reflectance spectrum of $[Co(en)_3]Cl_3$ (containing **B3**) after KM transformation and its Gaussian analysis in the 12–32 $\times 10^3$ cm^{-1} range. The arrow shows band maximum position of the lowest d–d transition ($^1A_{1g} \rightarrow {}^3T_{1g}$, $\tilde{\nu}_{max} = 13.8 \times 10^3$ cm^{-1}).

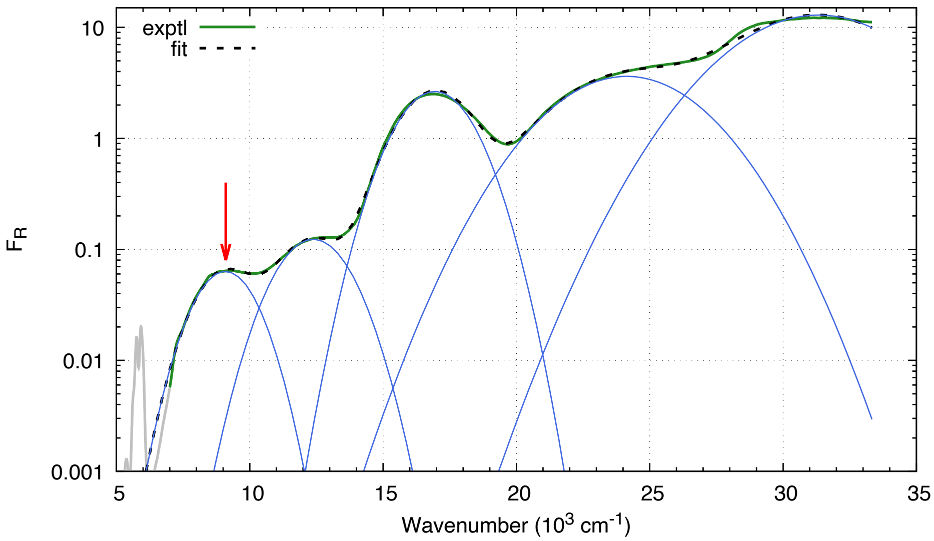

Figure S4: Reflectance spectrum of $[Co(acac)_3]$ (**B4**) after KM transformation and its Gaussian analysis in the $\geq 7 \times 10^3$ cm^{-1} range. Sharp bands near 6×10^3 are vibrational overtones. The arrow shows band maximum position of the lowest d–d transition ($^1A_{1g} \rightarrow {}^3T_{1g}$, $\tilde{\nu}_{max} = 9.1 \times 10^3$ cm^{-1}).

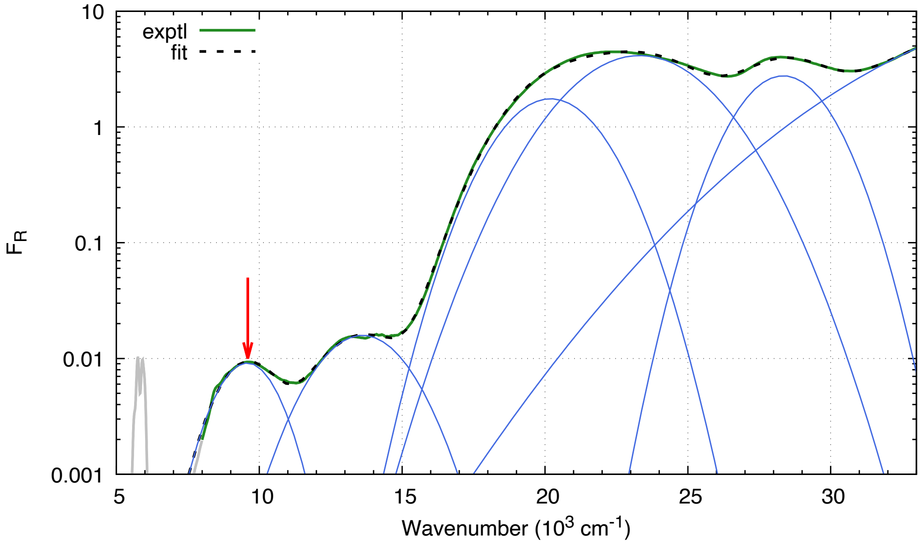

Figure S5: Reflectance spectrum of [Fe(acac)$_3$] (**C1**) after KM transformation and its Gaussian analysis in the $\geq 8 \times 10^3$ cm^{-1} range. Sharp bands near 6×10^3 are vibrational overtones. The arrow shows band maximum position of the lowest d–d transition ($^6A_{1g} \rightarrow {}^4T_{1g}$, $\tilde{\nu}_{\max} = 9.6 \times 10^3$ cm^{-1}).

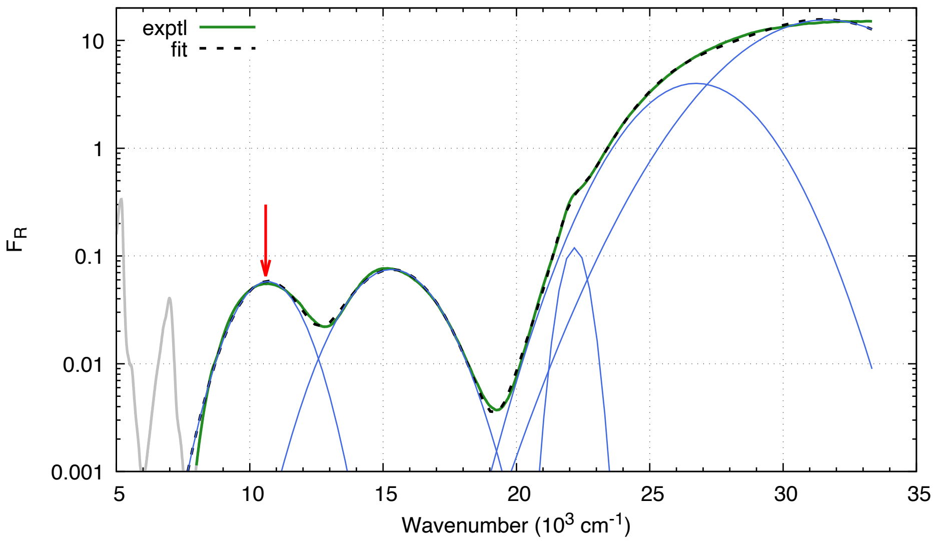

Figure S6: Reflectance spectrum of $K_3[Fe(ox)_3] \cdot 3\,H_2O$ (containing **C2**) after KM transformation and its Gaussian analysis in the $\geq 8 \times 10^3$ cm^{-1} range. Sharp bands near 5 and 6×10^3 cm^{-1} are vibrational overtones or combination bands. The arrow shows band maximum position of the lowest d–d transition ($^6A_{1g} \rightarrow {}^4T_{1g}$, $\tilde{\nu}_{\max} = 10.6 \times 10^3$ cm^{-1}).

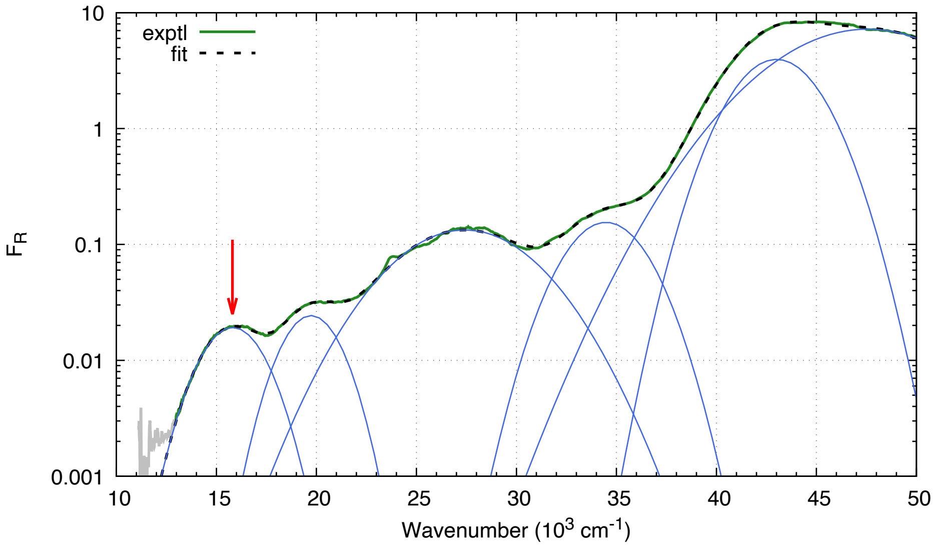

Figure S7: Reflectance spectrum of $[Mn(en)_3]Cl_2 \cdot H_2O$ (containing **C3**) after KM transformation and its Gaussian analysis in the 12.9–50 $\times$ 10^3 cm^{-1} range. The arrow shows band maximum position of the lowest d–d transition ($^6A_{1g} \rightarrow {}^4T_{1g}$, $\tilde{\nu}_{max} = 15.8 \times 10^3$ cm^{-1}).

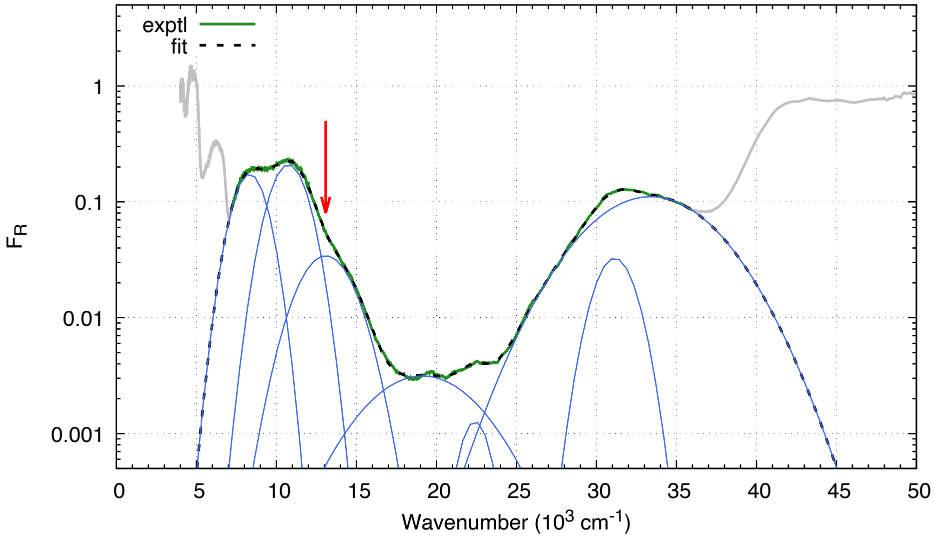

Figure S8: Reflectance spectrum of $(NH_4)_2Fe(SO_4)_2(H_2O)_6$ (containing **C4**) after KM transformation and its Gaussian analysis in the 7.2–36 $\times$ 10^3 cm^{-1} range. The arrow shows band maximum position of the lowest spin-forbidden d–d transition ($^5T_{2g} \rightarrow^3 T_{1g}$, $\tilde{\nu}_{max} = 13.1 \times 10^3$ cm^{-1}), which is overlapped on the higher-energy side of the spin-allowed $^5T_{2g} \rightarrow^5 E_g$ band split due to dynamic JT effect.[S4] Sharp bands near 5×10^3 cm^{-1} are vibrational overtones or combination bands.

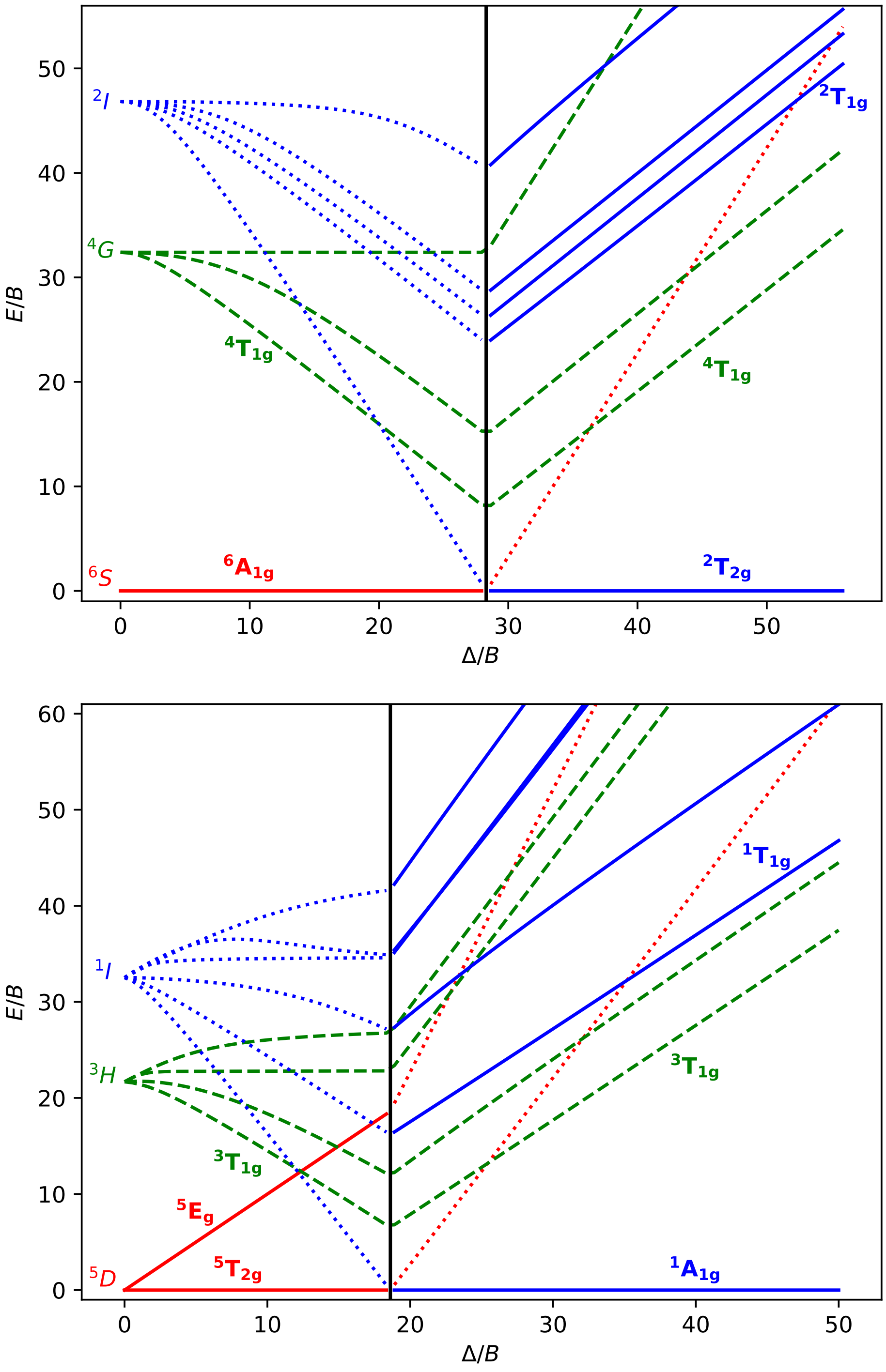

Figure S9: Simplified Tanabe–Sugano diagrams for d^5 (*top*) and d^6 (*bottom*) octahedral complexes, supporting identification of the lowest-energy singly spin-forbidden d–d transitons. The energy levels are drawn as solid lines for states with the same multiplicity as the ground state; as dashed lines for excited states providing singly spin-forbidden transitions ($|\Delta S| = 1$); as dotted lines for excited states providing doubly spin-forbidden transitions ($|\Delta S| = 2$). Excited states providing the lowest spin-allowed and singly spin-forbidden transitions are labeled.

S1.2 Crystal structure of $[Mn(en)_3]Cl_2 \cdot H_2O$

Data acquisition and analysis. Diffraction intensity data for a single crystal of $[Mn(en)_3]Cl_2 \cdot H_2O$ was collected at 250 K on a Rigaku XtaLAB Synergy-S diffractometer with mirror-monochromated MoKα radiation ($\lambda = 0.71073$ Å). Cell refinement and data reduction were performed using firmware.[S5] The positions of all non-hydrogen atoms were determined using the SHELXT software (ver. 2018/3).[S6,S7] All non-hydrogen atoms were refined anisotropically using a weighted full least squares matrix on F^2. Refinement and further calculations were performed using the SHELXL software. All hydrogen atoms bonded to carbon atoms were aligned with idealized geometry and refined using a driving model with U_{iso}(H) fixed at 1.5 U_{eq}. The positions of the water molecule's hydrogen atoms were determined based on a differential Fourier map. The drawings were made with Diamond ver. 4.6.8.[S8] CCDC 2259710 contains additional crystallographic data for the described structure.

Crystal structure description. The crystallographic data and detailed information on the structure solution and refinement for the titled structure are given in Table S1. Selected bond parameters are summarized in Table S2.

The asymmetric part of the unit cell of the crystal of the title compound, crystallizing in the centrosymmetric space group Pbcn, consists of the $[Mn(en)_3]^{2+}$ cation in the occupancy of 0.5, the Cl^- anion and the crystallizing water molecule, where both molecules are fully occupied (Figure S10). The Mn atom, located in a special position on the 2-fold axis, is chelated with three ethylenediamine (en) ligands, which form the coordination environment of the central atom (Figure S11). The coordination geometry is best described as a slightly distorted trigonal antiprism (based on the geometric criteria given by Kepert[S9]).

Three Mn-N bond lengths are typical for the $[Mn(en)_3]^{2+}$ cation and are 2.2602(1) Å for N7, 2.3059(1) Å for N4 and 2.3030(1) Å for N8. The C–C bond lengths in the en ligands range from 1.5141(1)–1.5111(1) Å and are typical typical for these ligands in TM complexes. The C–N bond distances are in the range of 1.4716(1)–1.4756(1) Å and also show no deviation from typical values

observed for analogous $(en)_3$ complexes with TMs. The configuration of the cation $[Mn(en)_3]^{2+}$ is a typical arrangement of $\Delta(\lambda\lambda\lambda)$ and $\Lambda(\delta\delta\delta)$ in equal numbers, which results from the symmetry operators of the centrosymmetric space group.[S10] Based on the packing analysis, a V-shaped arrangement of Cl···O···Cl can be distinguished in the structure (Figure S12), in which individual molecules are connected by hydrogen bonds of the OH···Cl type (Table S3). This is a typical structural motif occurring in systems containing Cl^- anions and water molecules, which was confirmed based on the resources of the CSD database (6461 structures, ConQuest Ver. 2022.3.0, Build 364735).

Table S1: Crystal data and structure refinement for $[Mn(en)_3]Cl_2 \cdot H_2O$ (CCDC 2259710)

Empirical formula	$C_6H_{26}Cl_2MnN_6O$	
Formula weight	324.17	
Temperature	250(2) K	
Wavelength	0.710 73 Å	
Crystal system	Orthorhombic	
Space group	Pbcn	
Unit cell dimensions	$a = 11.9372(2)$ Å	$\alpha = 90°$
	$b = 14.3092(2)$ Å	$\beta = 90°$
	$c = 8.86370(10)$Å	$\gamma = 90°$
Volume	1514.02(4) $Å^3$	
Z	4	
Density (calculated)	1.422 Mg/m^3	
Absorption coefficient	1.218/mm	
F(000)	684	
Crystal size	0.150 x 0.100 x 0.100 mm^3	
Theta range for data collection	2.222 to 30.729°	
Index ranges	$-15 \leq h \leq 17$, $-20 \leq k \leq 20$, $-12 \leq l \leq 12$	
Reflections collected	62494	
Independent reflections	2233 [R(int) = 0.0371]	
Completeness to theta = 25.242°	99.6 %	
Refinement method	Full-matrix least-squares on F^2	
Data / restraints / parameters	2233 / 0 / 78	
Goodness-of-fit on F^2	1.054	
Final R indices [I>2sigma(I)]	R1 = 0.0238, wR2 = 0.0632	
R indices (all data)	R1 = 0.0303, wR2 = 0.0658	
Largest diff. peak and hole	0.256 and –0.343 e/$Å^3$	

Table S2: Bond lengths (Å), angles (degree) and torsion angles (°) for $[Mn(en)_3]Cl_2 \cdot H_2O$ (CCDC 2259710).

Mn(1)-N(7)#1	2.2602(8)
Mn(1)-N(7)	2.2602(8)
Mn(1)-N(8)	2.3030(9)
Mn(1)-N(8)#1	2.3030(9)
Mn(1)-N(4)	2.3058(8)
Mn(1)-N(4)#1	2.3059(8)
O(1)-H(1O)	0.834(19)
O(1)-H(1O)#2	0.834(19)
N(7)-C(6)	1.4756(14)
N(4)-C(5)	1.4751(14)
N(8)-C(9)	1.4716(16)
C(5)-C(6)	1.5141(15)
C(9)-C(9)#1	1.511(3)
N(7)#1-Mn(1)-N(7)	172.15(4)
N(7)#1-Mn(1)-N(8)	92.22(3)
N(7)-Mn(1)-N(8)	93.94(3)
N(7)#1-Mn(1)-N(8)#1	93.94(3)
N(7)-Mn(1)-N(8)#1	92.22(3)
N(8)-Mn(1)-N(8)#1	76.46(5)
N(7)#1-Mn(1)-N(4)	97.46(3)
N(7)-Mn(1)-N(4)	77.31(3)
N(8)-Mn(1)-N(4)	93.74(3)
N(8)#1-Mn(1)-N(4)	165.27(3)
N(7)#1-Mn(1)-N(4)#1	77.31(3)
N(7)-Mn(1)-N(4)#1	97.46(3)
N(8)-Mn(1)-N(4)#1	165.27(3)
N(8)#1-Mn(1)-N(4)#1	93.74(3)
N(4)-Mn(1)-N(4)#1	97.84(5)
H(1O)-O(1)-H(1O)#2	98(3)
C(6)-N(7)-Mn(1)	109.73(6)
C(5)-N(4)-Mn(1)	107.09(6)
C(9)-N(8)-Mn(1)	108.97(7)
N(4)-C(5)-C(6)	109.38(8)
N(7)-C(6)-C(5)	109.57(8)
N(8)-C(9)-C(9)#1	109.81(8)
Mn(1)-N(4)-C(5)-C(6)	45.52(9)
Mn(1)-N(7)-C(6)-C(5)	40.61(9)
N(4)-C(5)-C(6)-N(7)	−59.32(11)
Mn(1)-N(8)-C(9)-C(9)#1	42.60(12)

[a] Symmetry transformations used to generate equivalent atoms: #1 -x+1,y,-z+1/2 #2 -x+1,y,-z+3/2.

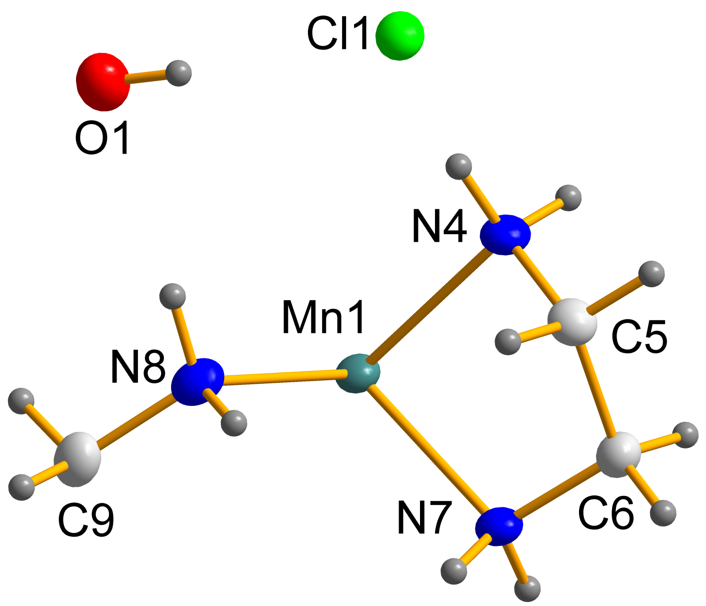

Figure S10: The asymmetric unit of $[Mn(en)_3]Cl_2 \cdot H_2O$. All non-hydrogen atoms are represented at 30% probability thermal ellipsoids.

Table S3: Hydrogen bonds for $[Mn(en)_3]Cl_2 \cdot H_2O$.

D–H···A	d(D–H) [a]	d(H···A) [a]	d(D···A) [a]	∠(DHA) [b]
N(7)-H(7A)...Cl(1)#3	0.90	2.51	3.3785(9)	161.6
N(7)-H(7B)...Cl(1)#4	0.90	2.45	3.3194(9)	163.5
N(4)-H(4A)...Cl(1)#5	0.90	2.88	3.5812(9)	135.5
N(4)-H(4B)...Cl(1)	0.90	2.67	3.4568(9)	147.1
N(8)-H(8A)...Cl(1)#3	0.90	2.62	3.4636(10)	156.9
N(8)-H(8B)...O(1)	0.90	2.35	3.2435(9)	174.2
C(6)-H(6A)...Cl(1)#5	0.98	2.85	3.5771(11)	131.5
O(1)-H(1O)...Cl(1)	0.834(19)	2.310(19)	3.1354(11)	170.7(17)

[a]Value in Å. [b]Value in °. Symmetry transformations used to generate equivalent atoms: #3 -x+1/2,-y+3/2,z-1/2; #4 x,y,z-1; #5 x,-y+1,z-1/2.

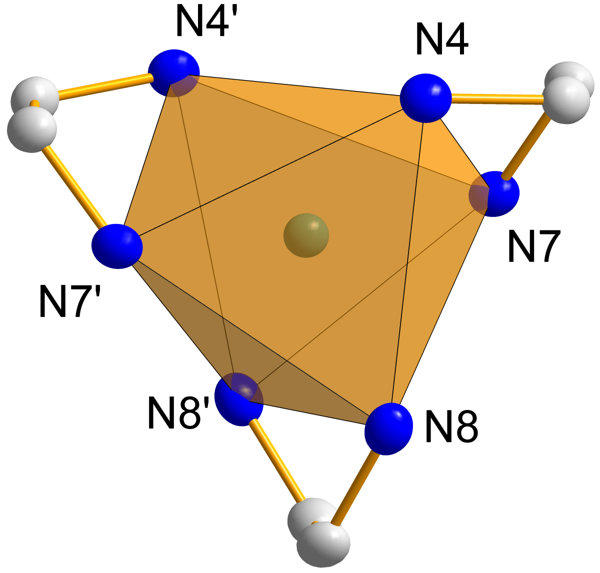

Figure S11: Structure of the cationic complex of the $[Mn(en)_3]^{2+}$ molecule. The coordination environment around the Mn atom has the shape of a slightly distorted trigonal antiprism. Nitrogen atoms are shown in blue and carbon atoms in gray. All H atoms have been omitted for clarity. All atoms other than hydrogen are represented by thermal ellipsoids with a probability of 30%. $'$: $[1-x, y, 1/2-z]$.

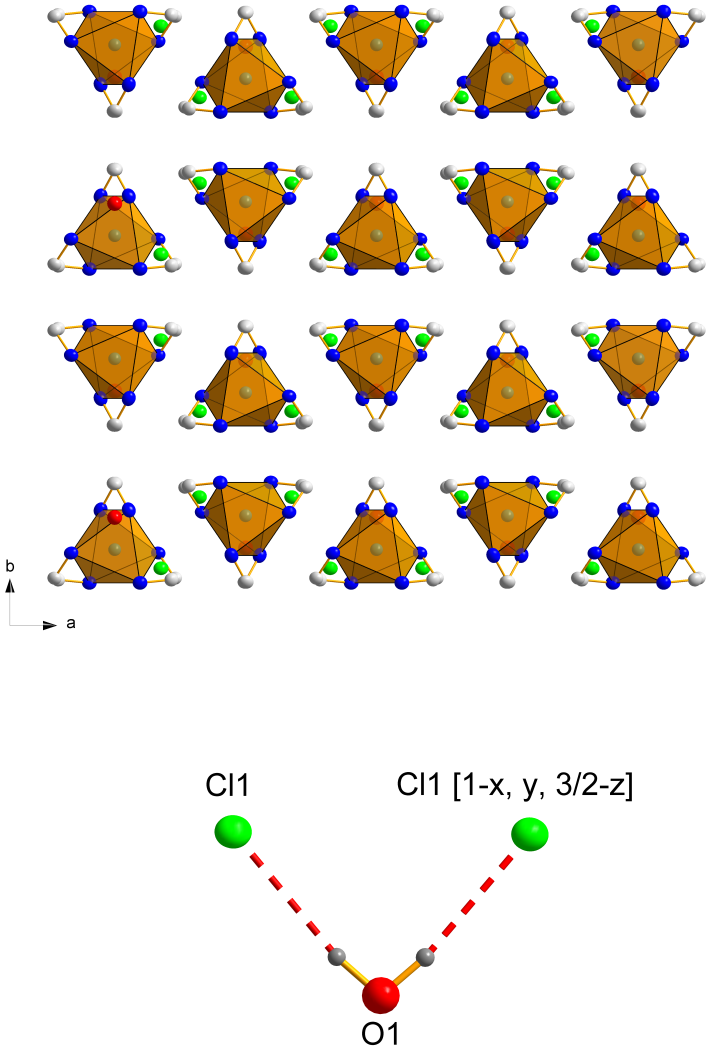

Figure S12: Top: packing of the structure seen in the [001] direction. Hydrogen atoms omitted for clarity. Coordination polyhedra around the manganese atoms are clearly visible, with the geometry of a trigonal antiprism. Bottom: V-shaped structural motif of Cl···HOH···Cl.

S2 Full Computational Details

S2.1 DFT Calculations

DFT calculations were performed using different programs depending on the choice of functional:

Turbomole v7.5 PBE,[S11] PBE0,[S12] M06,[S13] M06L,[S13] TPSS,[S14] TPSSh,[S15] CAM-B3LYP,[S16] ωB97X-D,[S17] ωB97X-V,[S18] ωB97M-V,[S19] LH14t-calPBE,[S20] LH20t,[S21] SCAN,[S22] and r2SCAN[S23]

Gaussian 16 B3LYP,[S24,S25] B3LYP*,[S26] OLYP,[S27] OPBE,[S27] B97-D3,[S28] MN15,[S29] MN15L,[S30] PW6B95D3,[S31] LC-ωPBE[S32–S34]

Orca v5.0 SSB-D,[S35,S36] S12g,[S37] S12h,[S37] MVS,[S38] MVSh,[S38] B2PLYP,[S39,S40] PWPB95,[S40] DSD-PBEP86,[S41,S42] DSD-PBEB95[S42]

After geometry optimization at the COSMO(∞)/PBE0-D3(BJ)/def2-TZVP level, single-point energy calculations in vacuum were performed using the def2-QZVPP basis set.[S43] The obtained nonrelativistic energy difference were corrected for scalar-relativistic effects by adding the Δ(DK) term determined at the second-order DK level using the B3LYP functional

$$\Delta(\mathrm{DK}) = \Delta E_{\mathrm{B3LYP/cc\text{-}pVTZ\text{-}DK}} - \Delta E_{\mathrm{B3LYP/cc\text{-}pVTZ}} \tag{S.3}$$

(where the basis set was either cc-pVTZ-DK[S44] in the relativistic calculations or cc-pVTZ[S45] in the nonrelativistic calculations). This approach is based on approximate independence of the scalar-relativistic correction on the theory level (e.g., the choice of functional).[S46–S50]

The following dispersion corrections were used:

none (due to the lack of parameters) in the case of MN15, MN15, MVS, MVSh

built-in (determined as a part of the functional construction) in the case of SSB-D, S12g, S12h, PW6B95D3, B97-D, ωB97X-D, ωB97X-V, and ωB97M-V.

D3 (with zero-damping[S51]) in the case of M06 and M06L

D3(BJ) (with Becke–Johnson damping[S52]) for all the remaining functionals

The SSB-D, S12g and S12h functionals in Orca were provided using the LibXC[S53] interface and the corresponding dispersion correction were manually added using the Grimme's DFT-D3 program.[S54] The set of dispersion parameters provided with this program was also used for the OLYP and OPBE, for which the dispersion parameters are not natively implemented in Gaussian. For B3LYP* and r2SCAN functionals we used identical D3(BJ) parameters as for the original B3LYP and SCAN functionals, respectively (note that similar choices were also made in refs S55 and S56). The DSD-PBEP86-D3(BJ) parameters are those from ref S42.

Note that ωB97X-V and ωB97M-V contain non-local, density dependent dispersion correction based on the VV10 model,[S57] which was applied self-consistently.

All Turbomole calculations employed the RIJ approximation.[S58,S59] The m5 integration grid was used, except for SCAN and r2SCAN calculations where a larger grid was used (gridsize 5, radsize 40). All Orca calculations employed the RIJ approximation for pure functionals or the RIJCOSX approximation[S60] for functionals containing the exact exchange, and the RI-MP2 approximation[S61] for double-hybrid functionals. The default integration grid (defgrid2) was used, except for MVS and MVSh (defgrid3).

Comment on the geometry of A9 For the D_{5h}-symmetric geometry of $MnCp_2$ (**A9**) in the sextet state, vibrational analysis at the PBE0-D3(BJ)/def2-TZVP level reveals two tiny imaginary frequencies ($i16\ cm^{-1}$). The proper energy minimum was found to be only 0.2 kcal/mol lower in energy for the C_{2v}-symmetric geometry. The distortion of the sextet state geometry from the D_{5h} to C_{2v} symmetry is *not* related to JT effect and is presumably an artifact of the D3(BJ) dispersion model, because it is not observed[S62] with the newer D4 one. We thus assumed the D_{5h}-symmetric geometry of $^6[MnCp_2]$ in further calculations.

Spin Contamination and Broken-Symmetry (BS) Solutions. DFT calculations for singlets were spin-restricted, otherwise spin-unrestricted, but leading usually to small spin-contamination (see provided $\langle S^2 \rangle$ values). Exceptions are complex **A8** in the singlet state and complex **C4** in the triplet state, for which BS solutions were obtained with considerable contamination due to, respectively, the triplet and quintet states. Natural orbitals of representative BS solution are provided in Figure S13. The BS energies were corrected using the Yamaguchi formula[S63] [eq. (S.4)]

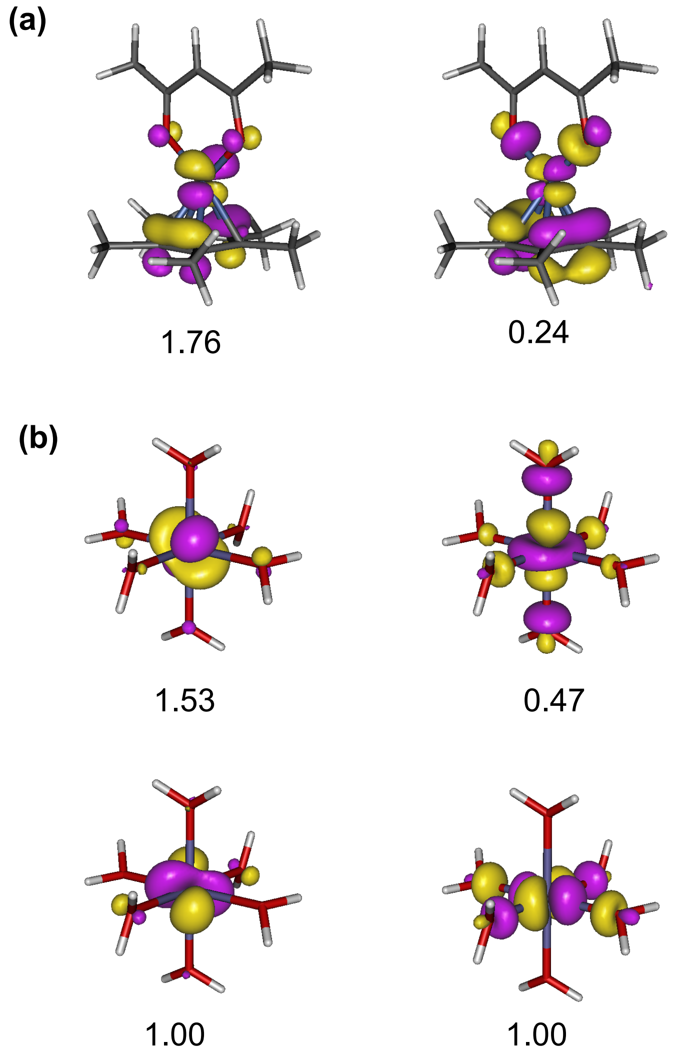

Figure S13: Contour plots of natural orbitals with occupation numbers (annotated) significantly deviating from 0 and 2 from BS PBE0/def2-TZVP solutions: (a) **A8** singlet and (b) **C4** triplet.

$$E_{2S+1}^{\text{corrected}} = E_{2S+1}^{\text{BS}} - \left(\langle S^2\rangle_{\text{BS}} - S(S+1)\right) \cdot \frac{E_{2S+3} - E_{2S+1}^{\text{BS}}}{\langle S^2\rangle_{2S+3} - \langle S^2\rangle_{\text{BS}}}. \tag{S.4}$$

Note that for singlet **A8** the spin-restricted solution was used in the geometry optimization step and to determine the vibrational and environmental corrections.

Details of COSMO calculations . We used the default value of rsolv parameter (1.3 Å) except for complexes **B1** and **B2** ($[Fe(CN)_6]^{3-/4-}$), for which rsolv was slightly increased (to 1.5 Å) in order to avoid problems with the SCF convergence. The following atomic radii were used: 2.223 Å for TM, 1.72 Å for O, 1.83 Å for N, 2.00 Å for C, and 1.30 Å for H atoms. The optimizations to provide molecular geometries of the complexes comprising the SSE17 benchmark set were performed with $\varepsilon = \infty$ to roughly describe the charge screening effect of a condensed phase on molecular geometries of TM complexes. Additional calculations to yield environmental corrections for SCO complexes **A1**, **A2**, **A4**, **A5**, **A7**, **A8**, **A9** in specific solvents (Section S3.2.1), were performed with the actual dielectric constant of each solvent: $\varepsilon = 4.711$ for $CHCl_3$, 8.93 for CH_2Cl_2, 7.426 for THF, 20.493 for acetone, 32.613 for MeOH, 35.688 for MeCN, 37.219 for DMF, and 78.355 for water.

S2.2 WFT Calculations

Basis sets. Table S4 gives description of the orbital basis sets cT(D)-DK and cT(D) used in WFT calculations and auxiliary basis sets for F12 calculations with the cT(D) orbital basis sets. Table S5 gives detailed results used to obtain final CCSD(T) energies (see eq. (1) and (2) in main article).

Singlet states in KS-CCSD(T) calculations. All KS-CCSD(T) calculations, even for singlet states, were performed using the open-shell coupled-cluster program of Molpro (uccsd) to ensure that the perturbative triples contributions to correlation energy are computed correctly. When using the closed-shell program for singlets with non-canonical orbitals, the triples contributions are significantly underestimated, at least with the 2015–2022 versions of Molpro tested by us.

Table S4: Basis Sets cT(D)-DK, cT(D), and Auxiliary Basis Sets for F12 Calculations

basis	TM	atoms bonded to TM	remaining atoms
cT(D)-DK	cc-pwCVTZ-DK [a]	cc-pVTZ-DK [b]	cc-pVDZ-DK [b,j]
cT(D)	cc-pwCVTZ [a]	cc-pVTZ [c]	cc-pVDZ [c]
cT(D), df_basis [d]	aug-cc-pVTZ/mp2fit [e]	aug-cc-pVTZ/mp2fit [f]	aug-cc-pVDZ/mp2fit [f]
cT(D), df_basis_exch [g]	def2-TZVPP/jkfit [h]	aug-cc-pVTZ/jkfit [i]	aug-cc-pVDZ/jkfit [i]
cT(D), ri_basis [j]	def2-TZVPP/jkfit [h]	aug-cc-pVTZ/optri [k]	aug-cc-pVDZ/optri [k]

[a] Ref. S64. [b] Ref. S44. [c] Ref. S45. [d] Density fitting auxiliary basis. [e] Ref. S65. [f] Ref. S66. [g] Density fitting auxiliary basis for the exchange and Fock operators. [h] Ref. S67. [i] Ref. S68. [j] Resolution-of-identity (RI) auxiliary basis. [k] Ref. S69. [j] For cluster models of **B1**, **B2** and **C2** in solid state, the basis set for potassium atoms was set to cc-pwCVDZ-DK (ref. S70) and their 3s3p electrons were correlated in CASPT2 calculations.

Table S5: Detailed CC Results.[a]

method	HF	CCSD	CCSD(T)		CCSD(T#)-F12a	CCSD(T)
basis<br>complex	cT(D)-DK	cT(D)-DK	cT(D)-DK	cT(D)	cT(D)	final [b]
**A1**	–85.8	–18.2	–5.1	–6.0	–1.5	–0.7
**A2**	–74.4	–9.5	3.9	1.1	5.1	7.9
**A3**	–70.6	–11.0	1.1	–1.4	2.8	5.4
**A4**	–66.6	–9.6	0.9	–1.2	2.7	4.8
**A5**	–77.1	–13.3	0.6	–2.4	1.1	4.1
**A6**	–76.1	–18.8	–4.1	–6.0	1.0	2.9
**A7**	–50.1	–10.7	–1.6	–3.0	–0.4	1.1
**A8**	–43.4	–5.8	2.9	2.6	5.0	5.3
**A9**	–106.9	–20.2	–3.0	–6.0	–0.3	2.7
**B1**	19.7	48.3	55.1	55.1	57.3	57.3
**B2**	45.3	67.5	73.3	72.8	74.5	75.0
**B3**	20.7	38.4	42.6	42.7	43.9	43.8
**B4**	9.8	24.7	28.4	28.4	29.4	29.4
**C1**	–75.3	–40.5	–32.2	–31.4	–28.8	–29.6
**C2**	–76.7	–42.8	–35.4	–34.6	–32.0	–32.8
**C3**	–72.6	–50.3	–47.0	–47.7	–45.5	–44.8
**C4**	–57.0	–40.8	–38.2	–38.7	–36.9	–36.4

[a] Values in kcal/mol. [b] Final estimate: $E^{\text{CCSD(\#T)-F12a}}_{\text{cT(D)}} + E^{\text{CCSD(T)}}_{\text{cT(D)-DK}} - E^{\text{CCSD(T)}}_{\text{cT(D)}}$, see main article eq. (1) and (2).

Treatment of TM outer-core (3s3p) correlation; CASPT2/CC and CASPT2+δMRCI methods. In all WFT calculations the core electrons on ligands and the inner-core electrons on TM atoms were frozen, but the outer-core electrons on TM atoms (3s3p) were correlated. In the case of CASPT2/CC method,[S71] the 3s3p electrons are frozen at the CASPT2 level, but their contribution to the correlation energy is recovered at the CCSD(T) level, i.e.

$$\Delta E^{\text{CASPT2/CC}}_{\text{cT(D)-DK}} = \Delta E^{\text{FC-CASPT2}}_{\text{cT(D)-DK}} + \Delta E^{\text{CCSD(T)}}_{\text{cT(D)-DK}} - \Delta E^{\text{FC-CCSD(T)}}_{\text{cT(D)-DK}}, \tag{S.5}$$

where the prefix FC (frozen-core) refers to calculations in which the 3s3p electrons are frozen. In the case of CASPT2+δMRCI calculations, the energies were calculated as

$$\Delta E^{\text{CASPT2+}\delta\text{MRCI}}_{\text{cT(D)-DK}} = \Delta E^{\text{CASPT2}}_{\text{cT(D)-DK}} + \Delta E^{\text{FI-MRCI+Q}}_{\text{cT(D)-DK}} - \Delta E^{\text{FI-CASPT2}}_{\text{cT(D)-DK}}, \tag{S.6}$$

where the prefix FI (frozen-inactive) indicates calculations in which all inactive orbitals except TM 3s3p are frozen in accord with the procedure proposed by Reimann and Kaupp.[S50,S72]

Active space selection. Table S6 gives detailed description of the active spaces used in the CASSCF calculations underlying CASPT2 and MRCI.

As explained in the main article, the default choice was to make active: (a) five valence TM 3d orbitals, (b) one or two mostly doubly-occupied ligand orbitals considerably overlapping with the TM 3d orbitals to form covalent metal-ligand combinations, and (c) up to five mostly virtual orbitals with the TM 4d character to describe the double–shell effect, in some complexes jointly with π-backdonation.

With regard to (c), it was attempted to make the active space identical for both spin states. However, in some cases it turned out necessary—for the sake of avoiding uncontrolled orbital rotations, significantly changing the character of orbitals and eventually leading to undesired orbitals in the active space—to reduce the number of correlating 4d orbitals for lower-spin states. In such cases, the following rule was adopted (similar to the literature[S71]): the 4d$_i$ orbital is made active

Table S6: Description of Active Spaces in Multiconfigurational Calculations.

complex	active space[a]	active orbitals[b]				remarks
		3d	4d	M–L	other	
**A1**	(9,10/12)[c]	5	3/5[c]	2	0	
**A2**	(10,10/12)[c]	5	3/5[c]	2	0	
**A3**	(10,10/12)[c]	5	3/5[c]	2	0	
**A4**	(10,10/12)[c]	5	3/5[c]	2	0	
**A5**	(10,10/12)[c]	5	3/5[c]	2	0	[d]
**A6**	(10,10/12)[c]	5	3/5[c]	2	0	
**A7**	(11,11/12)[c]	5	4/5[c]	2	0	
**A8**	(12,14)	5	5	2	2[e]	
**A9**	(9,12)	5	5[f]	2	0	
**B1**	(9,12)	5	5[f]	2	0	
	(9,10)[g]	5	3[f]	2	0	
**B2**	(10,12)	5	5[f]	2	0	
	(10,10)[g]	5	3[f]	2	0	
**B3**	(10,12)	5	5	2	0	
**B4**	(10,12)	5	5	2	0	
**C1**	(9,12)	5	5	2	0	
**C2**	(9,12)	5	5	2	0	
**C3**	(9,12)	5	5	2	0	[d]
**C4**	(10,12)	5	5	2	0	[d]

[a]The notation "(N_e, N_o)" is used, where N_e is the number of active electrons, N_o the number of active orbitals. [b]Number of active orbitals of a given type: 3d = TM 3d orbitals; 4d = correlating orbitals with TM 4d character to describe the double-shell effect, in some cases mixed with ligands' virtual orbitals to describe π-backdonation (see footnote *f*); M–L = σ-bonding combinations of TM 3d with ligand lone pairs or with π-type orbitals in **A8** and **A9**. [c]Slash-separated numbers apply to lower-spin and higher-spin state, respectively (see text). [d]Core-fixing procedure was applied (see text). [e]Orbitals with Cp π^* character. [f]4d orbitals of appropriate symmetry are mixed with Cp/CN π^* virtual orbitals to describe not only double-shell effect, but also π-backdonation. [g]The smaller active space was used only to calculate the environmental correction.

only if the corresponding 3d$_i$ orbital is occupied in the leading configuration. As the the number of occupied 3d orbitals is smaller in the LS state than in the HS state, the number of 4d orbitals was also reduced accordingly (to three or four) in order to obtain a stable active space. For example, in the majority of Fe^{II} complexes, the active space is (10,10) for the LS (singlet) state, but (10,12) for the HS (quintet) state; this is indicated in Table S6 as “(10,10/12)”.

For organometallic complex **A8**, we considered a larger active space (14,15) used by Pierloot with co-workers.[S73] However, this active space is quite large (for example, considering the cost of MRCI calculations) and we noticed that one of the active orbitals—the lone pair of the acac ligand—is nearly doubly occupied in the singlet and triplet spin states (occupation numbers 1.9978 and 1.9986, respectively). Making this orbital inactive, leading to a slighly smaller (12,14) active space, has very small influence on the singlet–triplet splitting at the CASPT2 level (see Table S7). We thus adopoted the (12,14) active space for **A8**.

Table S7: CASPT2(12,14) and (14,15) Singlet–Triplet Splittings for **A8**. [a,b]

	CASPT2[c]	FC-CASPT2[d]
(12,14)	5.1	4.8
(14,15)	4.8	4.6

[a]Values in kcal/mol. [b]cT(D)-DK basis set, DK scalar relativistic. [c]TM 3s3p correlated. [d]TM 3s3p not correlated.

In some cases (**A5**, **C3**, **C4**; as indicated in Table S6), it turned out necessary to prevent the TM 3s3p orbitals from entering the active space with aid of “core-fixing procedures” described before.[S73,S74] To this end, the orbitals in CASSCF calculations were optimized gradually, starting from the initial guess taken from converged CASSCF calculations with a smaller active space lacking the M–L orbitals (i.e., only the 3d and 4d orbitals were active), by iterating the following two-step procedure. In step #1, the TM 3s3p orbitals were kept fixed. In step #2, the M–L orbitals were kept fixed. The procedure was iterated until the CASSCF energies obtained after steps #1 and 2 were identical (just 2–3 iterations were usually enough). In order to keep fixed selected orbitals during CASSCF optimization, the `supersymmetry` keyword was used in OpenMolcas, whereas

the `freeze` keyword was used in Molpro. In the case of $[Fe(H_2O)_6]^{2+}$ (**C4**), the above core fixing procedure was limited to the 3s orbital (the 3p orbitals cannot mix with any active orbitals due to the C_i symmetry). However, for the corresponding cluster model $\{[Fe(H_2O)_6](SO_4)_8(NH_4)_{10}\}^{4-}$, it turned necessary to fix not only 3s, but also 2s; the same was then done also for $[Fe(H_2O)_6]^{2+}$ when calculating the δ_{env} term.

S3 Vibrational, Environmental, and Substituent Corrections

S3.1 Vibrational Corrections

S3.1.1 Case 1: Vibrational Enthalpy Corrections to Adiabatic Energies (A1–A9)

In this case the vibrational correction is the difference between the relative enthalpy (ΔH) and adiabatic electronic energy (ΔE_{ad}) of the two spin states. The correction was computed using the statistical thermodynamics expression based on the harmonic oscillator model[S75,S76]

$$\delta_{\mathrm{vibr}}^{(\mathrm{case\ 1})} = \sum_i \left(\delta H_i^{\mathrm{HS}} - \delta H_i^{\mathrm{LS}}\right), \tag{S.7}$$

$$\delta H_i^m = N_A \left(\frac{1}{2}\hbar\omega_i^m + \frac{\hbar\omega_i^m}{\exp\left(\frac{\hbar\omega_i^m}{k_B T}\right) - 1} \right), \tag{S.8}$$

where $\hbar$ is the reduced Planck constant, N_A the Avogadro constant, k_B the Boltzmann constant, ω_i^m is the vibrational frequency of the i-th normal mode in a given spin state m (HS or LS), and the sum in (S.7) runs over the normal modes. The vibrational frequencies were computed at the PBE0-D3(BJ)/def2-TZVP level in vacuum. To be most consistent with the experimental determination of the ΔH value, the temperature assumed in (S.8) was the mid-range of temperatures in the experimental measurements or, in the case of **A3** and **A5** showing abrupt transitions, the experimental transition temperature. The quasi-harmonic approach of Cramer and Truhlar (QH/CT)[S77] was used, i.e., all frequencies smaller than 50 cm^{-1} were raised to the threshold value. The low frequencies raised in QH/CT approach are of small importance for the differential enthalpy correction (except at very low temperatures, which are not of our present interest)[S76] and this approach automatically solves the problem with the two tiny imaginary frequencies observed in the HS state of **A9** ($MnCp_2$, see above), which are replaced with the threshold value. Models with simplified ligands were used to obtain the δ_{vibr} corrections for **A6** and **A7**.

S3.1.2 Case 2: Vibronic Corrections to Vertical Energies (B1–B4, C1–C4)

In this case the vibrational correction is the difference between the energy corresponding to the absorption band maximum ($\Delta E_{\max}$) and the underlying vertical energy difference (ΔE_{ve}):

$$\delta_{\mathrm{vibr}}^{(\mathrm{case}\ 2)} = \Delta E_{\max} - \Delta E_{\mathrm{ve}}. \tag{S.9}$$

Note that under the adopted sign convention for energy differences, both $\Delta E_{\max}$ and ΔE_{ve} quantities are negative for complexes with HS ground state (**C1–C4**). To find relation between the two quantities and thereby determine the δ_{vibr} correction, we simulated vibrational progressions of the interesting spin-forbidden d–d transitions using a similar approach as introduced in our previous study.[S78] For consistency with the harmonic approximation assumed throughout the vibronic simulations to determine the $\Delta E_{\max}$ term, the ΔE_{ve} term in (S.9) is also understood as harmonic approximation of the vertical energy difference,[S78] i.e.

$$\Delta E_{\mathrm{ve}} = \Delta E_{\mathrm{ve}}^{\mathrm{harm}} \equiv \Delta E_{\mathrm{ad}} \pm \Delta E_{\mathrm{rlx}}^{\mathrm{harm}}, \tag{S.10}$$

where the $\pm$ sign applies to complexes with LS or HS ground state, respectively (due to the sign convention), ΔE_{ad} is the adiabatic energy difference, and the $\Delta E_{\mathrm{rlx}}^{\mathrm{harm}}$ term is the harmonic approximation of the relaxation energy on the excited state surface, i.e. assuming quadratic dependence of energy on mass-weighted displacements along the excited-state normal modes ($\Delta q_i^{\mathrm{exc}}$)

$$\Delta E_{\mathrm{rlx}}^{\mathrm{harm}} = \tfrac{1}{2} N_A \sum_i (\omega_i^{\mathrm{exc}} \Delta q_i)^2 = \tfrac{1}{2} N_A \hbar \sum_i \omega_i^{\mathrm{exc}} S_i^{\mathrm{exc}}, \tag{S.11}$$

where ω_i^{exc} are the excited-state frequencies and S_i^{exc} are the corresponding Huang–Rhys factors.

The vibronic absorption spectra were simulated using time-dependent (TD) approach[S79,S80] with Gaussian broadening, as implemented in the FCclasses code of Santoro and Cerezo,[S81] based on the adiabatic hessian (AH) approach within the Franck–Condon (FC) approximation (i.e., assuming that the intensity of an elementary transition is proportional to the overlap integral between

the initial and final vibrational states), taking into account the Duschinsky rotation of normal modes and the change of vibrational frequencies with electronic excitation. The underlying geometries and harmonic frequencies were calculated at the PBE0-D3(BJ)/def2-TZVP level in vacuum, except for $[Fe(CN)_6]^{4-}$ (**A2**), for which COSMO frequencies were used (due to observed dissociation of the triplet excited state in vacuum). The vibrational analysis in FCclasses was performed using internal coordinates,[S82,S83] except for $[Fe(CN)_6]^{3-/4-}$ (**A1**, **A2**), for which Cartesian coordinates were used. The list of internal coordinates was automatically generated using an in-house program based on the algorithm of Bakken and Helgaker.[S84] The use of internal coordinates was found important for complexes with larger organic ligands, similar to analogous reports in the literature for flexible organic molecules.[S82,S83]

Simulated vibrational progressions for the interesting spin-forbidden d–d transitions of complexes **B1**–**B4** and **C1**–**C4** are shown in Figures S14–S21. Note that the quantity presented on the horizontal axis is $E_\gamma \mp \Delta E_{\text{ve}}$, where $E_\gamma = N_A hc\tilde{\nu}$ is the energy of the absorbed photon with wave number $\tilde{\nu}$ (converted to kcal/mol); the sign $\mp$ applies to complexes with LS or HS ground state, respectively (due to the sign convention). Thus, the zero on the energy axis corresponds to the underlying vertical energy (graphically represented in as vertical solid line). Table S8 summarizes the vibronic corrections and also reports differential ZPEs and harmonic relaxation energies.

Table S8: Calculated Vibronic Corrections, Differential ZPEs and Relaxation Energies. [a]

	transition	δ_{vibr} [b]	ΔZPE [c]	$\Delta E_{\text{rlx}}^{\text{harm}}$ [d]
**B1**	doublet → quartet	−2.3	−2.6	13.0
**B2**	singlet → triplet	−2.9	−3.4	14.1
**B3**	singlet → triplet	−2.1	−2.4	8.0
**B4**	singlet → triplet	−1.8	−1.8	7.1
**C1**	sextet → quartet	−0.2	−1.2	10.9
**C2**	sextet → quartet	−0.2	−0.2	6.6
**C3**	sextet → quartet	−1.6	−1.2	13.8
**C4**	quintet → triplet	−0.2	−0.4	2.1

[a] All values in kcal/mol. [b] Vibronic correction, eq. (S.9), sign convention applies. [c] Zero point vibrational energy difference between the higher-spin and the lower-spin states. [d] Harmonic approximation of the relaxation energy on the excited state surface, eq. (S.11).

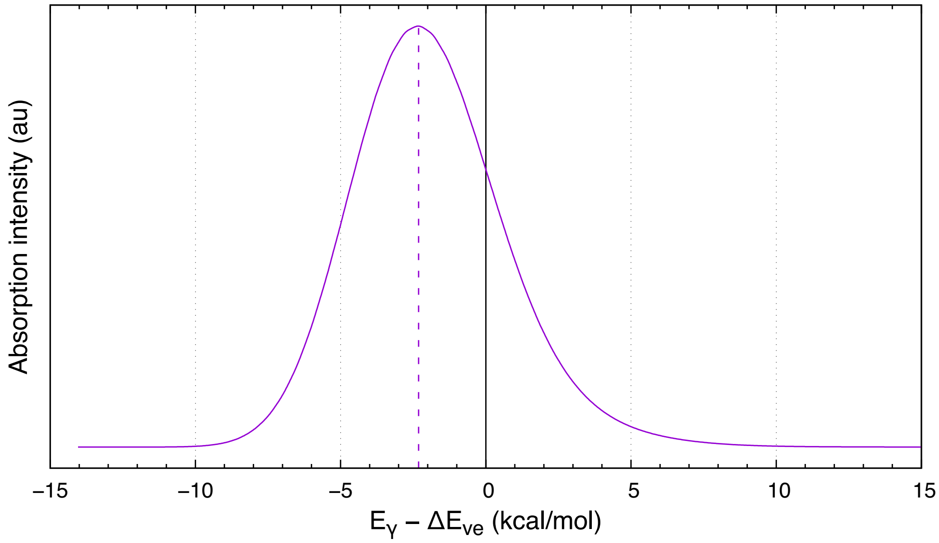

Figure S14: Vibrational progression of the lowest doublet → quartet transition for $[Fe(CN)_6]^{3-}$. Absorption spectrum based on FC vibrational overlap integrals, computed within AH model, was convoluted with a Gaussian function with HWHM = 0.015 eV.

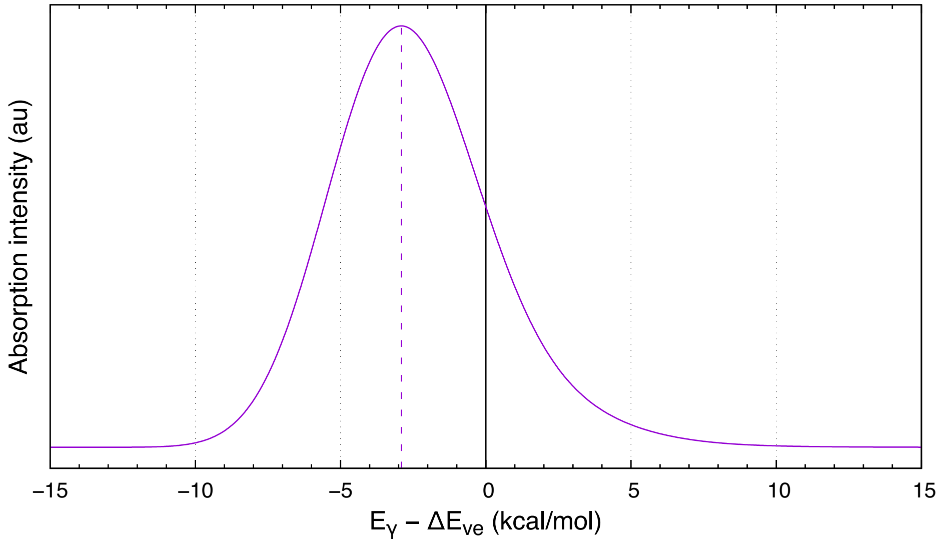

Figure S15: Vibrational progression of the lowest singlet → triplet transition for $[Fe(CN)_6]^{4-}$. Absorption spectrum based on FC vibrational overlap integrals, computed within AH model, was convoluted with a Gaussian function with HWHM = 0.015 eV.

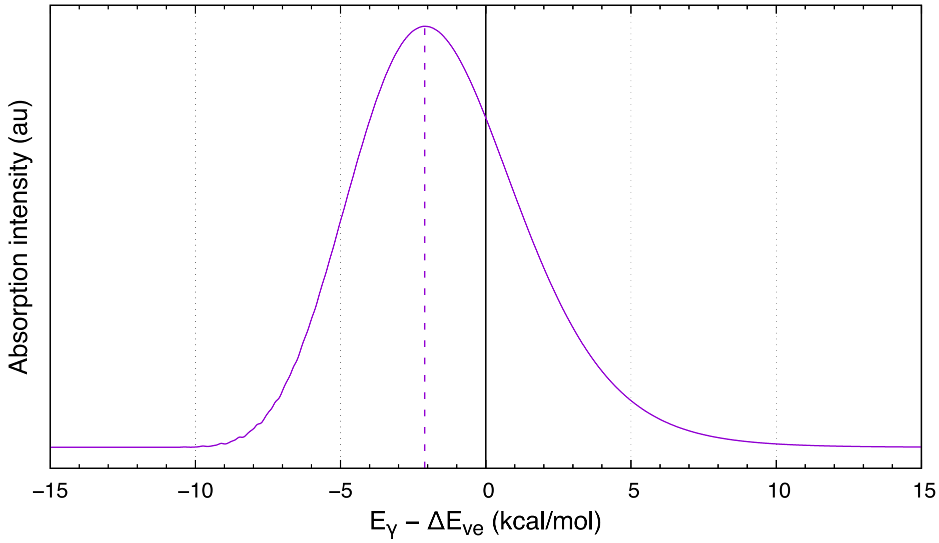

Figure S16: Vibrational progression of the lowest singlet → triplet transition for $[Co(en)_3]^{3+}$. Absorption spectrum based on FC vibrational overlap integrals, computed within AH model, was convoluted with a Gaussian function with HWHM = 0.005 eV.

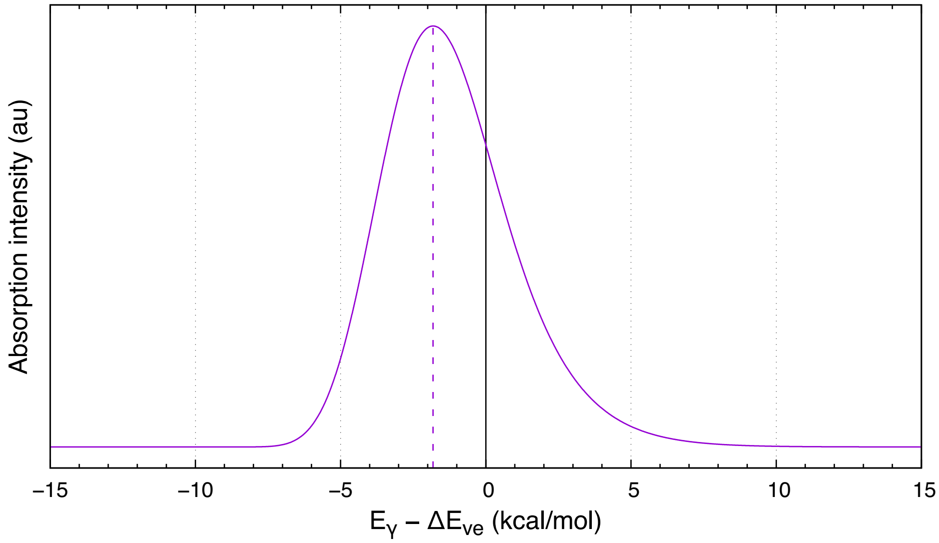

Figure S17: Vibrational progression of the lowest singlet → triplet transition for $[Co(acac)_3]$. Absorption spectrum based on FC vibrational overlap integrals, computed within AH model, was convoluted with a Gaussian function with HWHM = 0.005 eV.

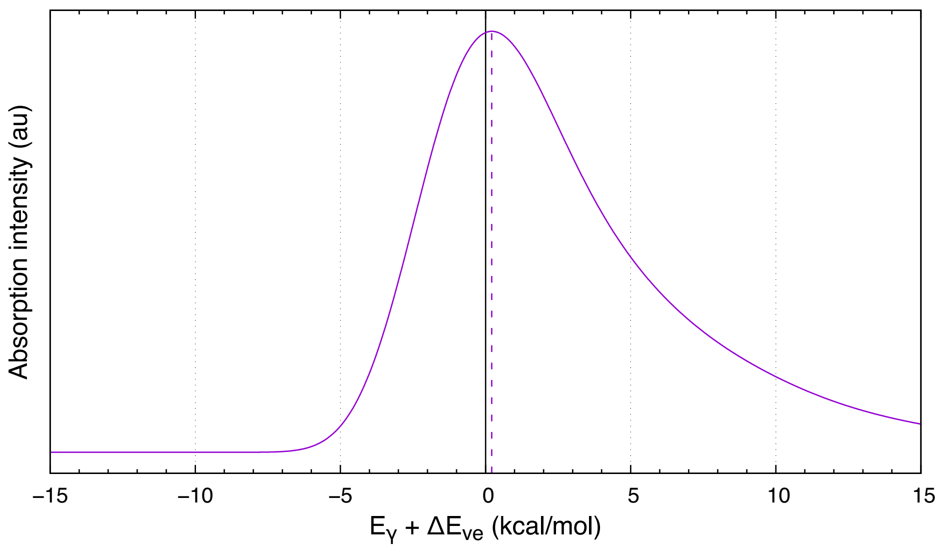

Figure S18: Vibrational progression of the lowest sextet → quartet transition for [Fe(acac)$_3$]. Absorption spectrum based on FC vibrational overlap integrals, computed within AH model, was convoluted with a Gaussian function with HWHM = 0.005 eV.

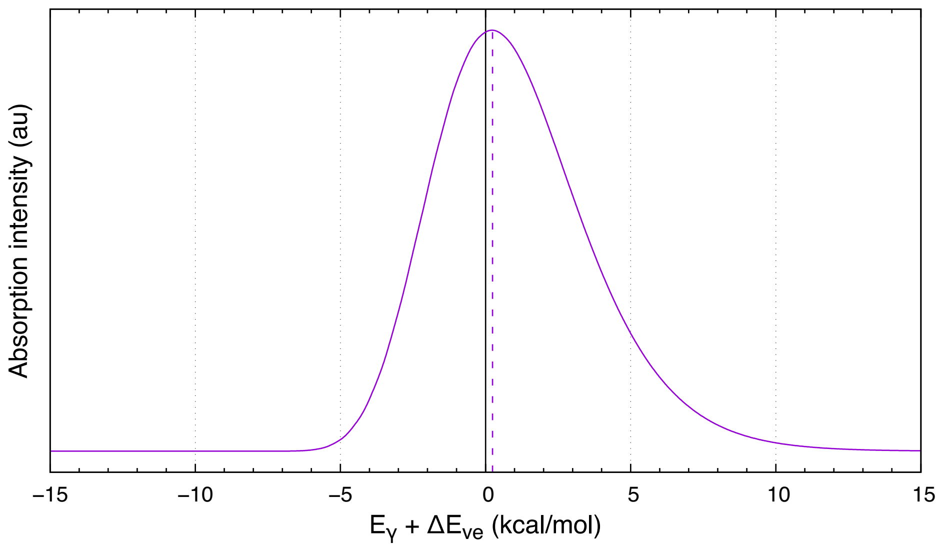

Figure S19: Vibrational progression of the lowest sextet → quartet transition for $[Fe(ox)_3]^{3-}$. Absorption spectrum based on FC vibrational overlap integrals, computed within AH model, was convoluted with a Gaussian function with HWHM = 0.015 eV.

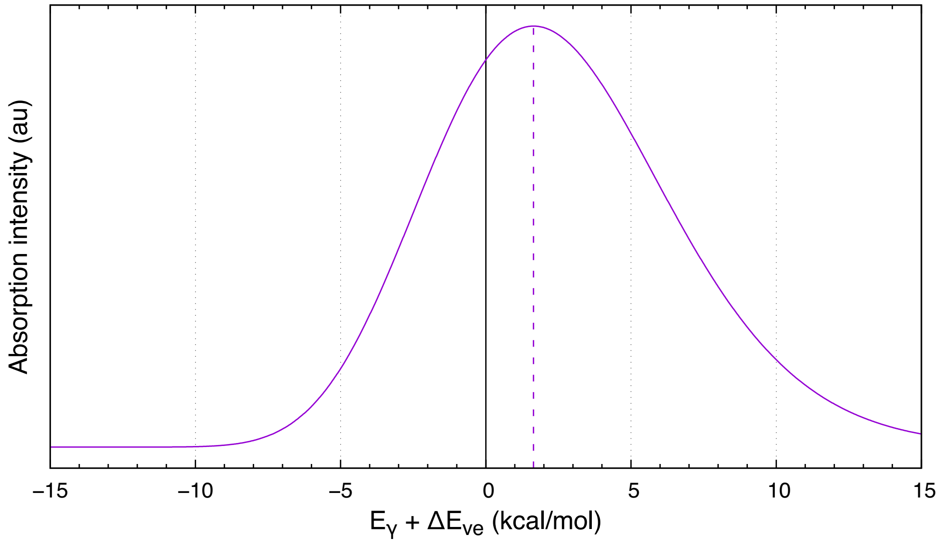

Figure S20: Vibrational progression of the lowest sextet → quartet transition for $[Mn(en)_3]^{2+}$. Absorption spectrum based on FC vibrational overlap integrals, computed within AH model, was convoluted with a Gaussian function with HWHM = 0.010 eV.

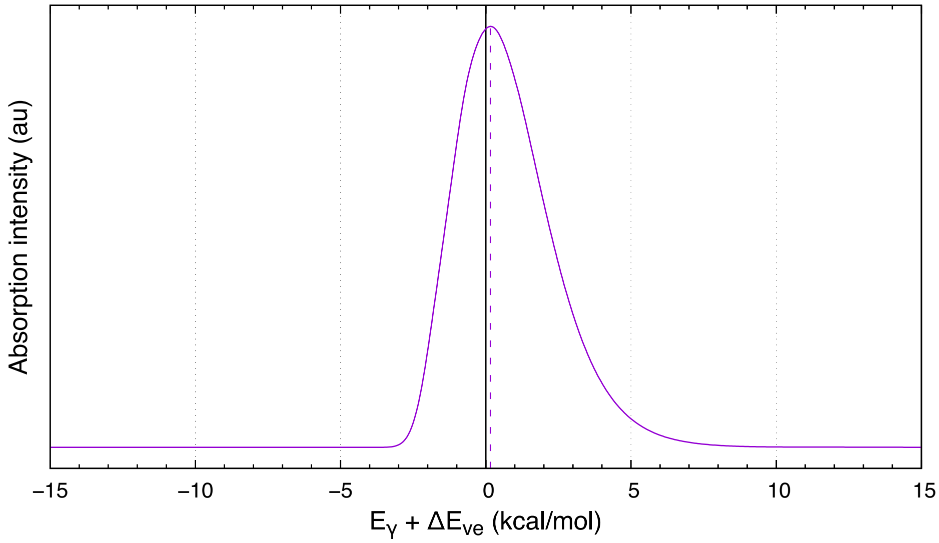

Figure S21: Vibrational progression of the lowest quintet → triplet transition for $[Fe(H_2O)_6]^{2+}$. Absorption spectrum based on FC vibrational overlap integrals, computed within AH model, was convoluted with a Gaussian function with HWHM = 0.025 eV.

S3.2 Environmental Corrections

S3.2.1 Case 1a: SCO in Solution (A1, A2, A4, A5, A7–A9)

In this case, the δ_{env} correction describes the influence of solvation on the adiabatic energy difference, i.e.

$$\delta_{\text{env}}^{(\text{case 1a})} = \Delta E_{\text{ad}}^{\text{solv}} - \Delta E_{\text{ad}}^{\text{vac}}. \tag{S.12}$$

The calculations to determine the correction were performed at the PBE0-D3(BJ)/def2-TZVP level. The energy in vacuum ($\Delta E_{\text{ad}}^{\text{vac}}$) was computed for the COSMO(∞)/PBE0-D3(BJ)/def2-TZVP geometries. This choice is consistent with the use of the COSMO(∞)/PBE0-D3(BJ)/def2-TZVP geometries in all further single-point WFT and DFT calculations of the benchmark study. The energy in solution ($\Delta E_{\text{ad}}^{\text{solv}}$) was calculated within the COSMO model of each solvent with geometries reoptimized for each ε value (see page S-21 for the list of dielectric constants and further details of the COSMO calculations). For complexes **A1** and **B4**, containing solvent-exposed N−H groups, which are potential H-bond donors, and solvents containing negatively-charged O or N atoms, which are potential H-bond acceptors (water, DMF, MeCN, MeOH, acetone, THF), we used microsolvation approach[S85] and added one solvent molecule interacting with each N−H group. This gives a difference of 0.2 to 1.1 kcal/mol in the δ_{env} correction as compared with straight COSMO calculations. Models used in the microsolvation calculations are shown in Figures S22 and S23, whereas their atomic coordinates are available in the ioChem-BD collection at `https://doi.org/10.19061/iochem-bd-7-8`. Table S9 summarizes the results of calculation leading to determination of the δ_{env} solvation corrections.

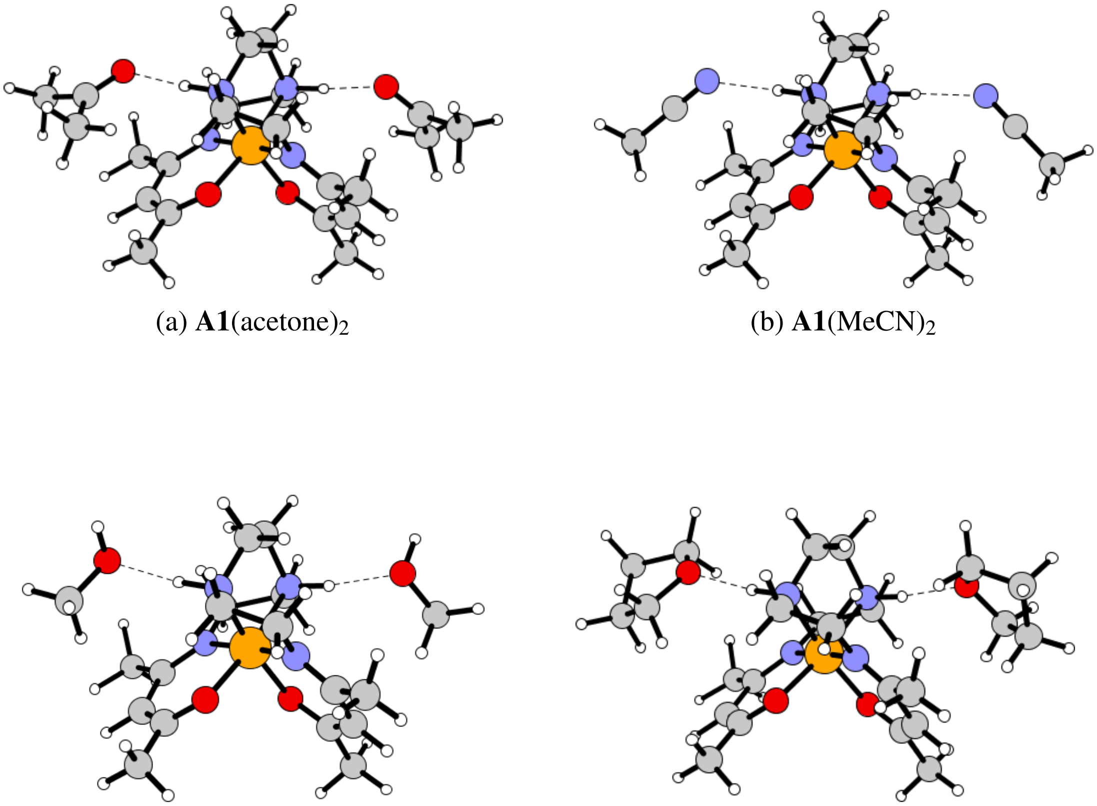

(a) **A1**(acetone)$_2$ (b) **A1**(MeCN)$_2$

(c) **A1**(MeOH)$_2$ (d) **A1**(THF)$_2$

Figure S22: Optimized structures of models used in microsolvation calculations for **A1**.

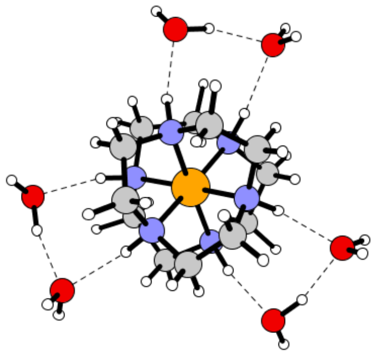

(a) **A4**(H_2O)$_6$

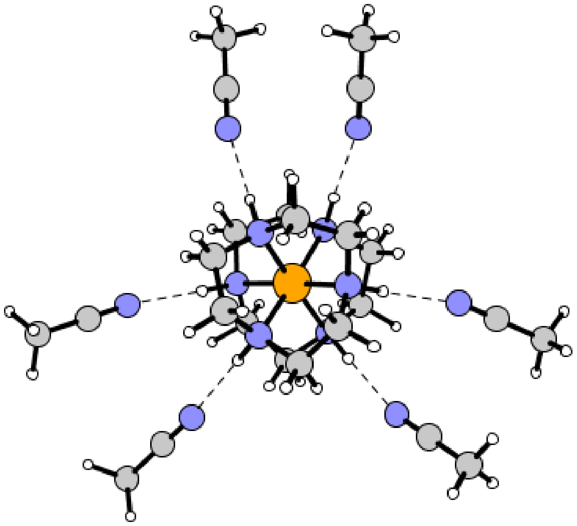

(b) **A4**(MeCN)$_6$

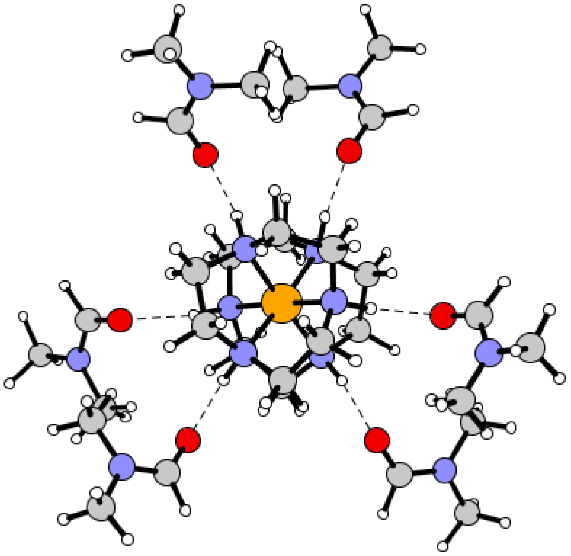

(c) **A4**(DMF)$_6$

Figure S23: Optimized structures of models used in microsolvation calculations for **A4**.

Table S9: Solvation Corrections for SCO Complexes in Solution.[a]

complex	environment	ΔE_{ad} [b]	δ_{env} [c,d]	
**A1**	vacuum	−6.7		
	DCM	−6.0	0.7	
	MeCN	−5.7	1.0	(0.7)
	MeOH	−5.7	1.0	(0.7)
	THF	−5.7	1.0	(0.6)
	acetone	−5.8	0.9	(0.7)
**A2**	vacuum	−1.8		
	CHCl3	−2.0	−0.2	
**A4**	vacuum	−7.2		
	DMF	−4.7	2.4	(2.9)
	MeCN	−5.4	1.8	(2.9)
	water	−4.8	2.4	(3.0)
**A5**	vacuum	−6.6		
	acetone	−4.6	2.0	
**A7**	vacuum	−4.6		
	acetone	−3.5	1.1	
**A8**	vacuum	−5.9		
	toluene	−6.1	−0.2	
**A9**	vacuum	−9.2		
	toluene	−9.1	0.2	

[a]Values in kcal/mol. [b]Energy difference between the considered spin states calculated at the PBE0-D3(BJ)/def2-TZVP level, either in vacuum or in solvent (see text). [c]Solvation effect, eq. (S.12). [d]Value in parenthesis is without explicit solvent molecules.

S3.2.2 Case 1b: SCO in Solid State (A3, A5–A7)

In this case, the δ_{env} correction describes the influence of crystal packing on the adiabatic energy difference, i.e.

$$\delta_{\text{env}}^{(\text{case 1b})} = \Delta E_{\text{ad}}^{\text{cryst}} - \Delta E_{\text{ad}}^{\text{vac}}. \tag{S.13}$$

The calculations to determine the correction were performed using periodic DFT+U calculations, following a similar methodology as recently proposed by Vela *et al.*[S86] To this end, periodic plane-wave calculations at Γ-point were performed using the PBE functional[S11] with the Grimme's D3(BJ) dispersion correction[S52] and the Hubbard-type correction (DFT+U) in the simplified version of Cococcioni and de Gironcoli,[S87] employing Vanderbilt's ultrasoft pseudopotentials from the GBRV library.[S88] The desired spin state was calculated by setting the total magnetization per unit cell to either 0 (singlet), 2Z (doublet), 3Z (quartet) or 4Z (quintet), where here Z is the number of TM sites per unit cell in a given crystal structure. In the first step, variable-cell geometry optimizations using the BFGS algorithm were performed with the U value set to 2.65 eV (recommended by Vela *et al.* for Fe^{II} complexes[S86]) starting from the experimental crystal structures containing **A3**, **A5**, **A6′** **A7′** (ref codes in the Cambridge Crystallography Data Center database: BAXFIS,[S89] XENBEX,[S90] QOVKIW,[S91] IQICEQ[S92]). The cutoffs for expansion of the wave function and density in the plane wave basis set were set to 70 Ry (ecutwfc) and 560 Ry (ecutrho), respectively. In the second step, the optimized crystalline geometries of each spin state were used in single-point calculations of the adiabatic energy difference in the crystal (the $\Delta E_{\text{ad}}^{\text{cryst}}$ term in eq. (S.13)) with the ecutwfc set to 35 Ry and ecutrho set to 280 Ry. In these calculations, the U parameter was successively varied to find the value of U which best reproduces the experimental estimate of the adiabatic energy difference in the crystal (i.e., $\Delta H - \delta_{\text{vibr}}$).[S86] This optimized U value and the plane-wave basis set with identical cutoffs were used to compute the adiabatic energy difference for isolated molecule of **A3**, **A5**, **A6′** or **A7′** in vacuum (the $\Delta E_{\text{ad}}^{\text{vac}}$ term in eq. (S.13)) using the COSMO/PBE0-D3(BJ)/def2-TZVP geometries of both spin states. In these calculations, the isolated molecule was placed in the cubic lattice with a large cell dimension (60 bohr) to reduce

unphysical interaction with its periodic images and the Makov–Payne correction[S93] was additionally used to correct the energy for long-range electrostatics. The U values optimized for each case and the resulting crystal packing corrections are given in Table S10. It was observed that even if the spin–state splittings are quite sensitive to the adopted value of U, the resulting $\delta_{\rm env}$ corrections are much less sensitive (see below, Table S15).

Note that since isolated molecule are computed in their relaxed equilibrium geometries, the present way of defining the crystal packing effect (CPE) is different than in ref S86, where isolated molecules were computed using their crystalline geometries (i.e., molecules excised from crystals). The present way of defining the CPE with respect to the COSMO/PBE0-D3(BJ)/def2-TZVP geometries is consistent with the use of these geometries in all further single-point WFT and DFT calculations of the benchmark study.

Table S10: Crystal Packing Effects for SCO Complexes in Solid State.

	compound	refcode [a]	U [b]	$\Delta E_{\rm ad}^{\rm vac}$ [c]	$\Delta E_{\rm ad}^{\rm cryst}$ [d]	$\delta_{\rm env}$ [e]
**A3**	$[Fe(HB(tz)_3)_2]$	BAXFIS[01]	2.37	5.3	4.8	–0.5
**A5**	$[Fe(1\text{-}bpp)_2][BF_4]_2$	XENBEX03	2.13	5.5	5.2	–0.4
**A6**	[K(222)][Fe(TPP)(CN)]	QOVKIW[03]	2.09	4.6	4.6	0.0
**A7**	$[Co(ipimpy)_2](ClO_4)_2$	IQICEQ	3.70	2.1	3.4	1.3

[a]Refcode in the CSD database for the CIF describing the experimental crystal structure. [b]Hubbard parameter, value in eV. [c]Energy difference for isolated molecule (for the COSMO/PBE0-D3(BJ)/def2-TZVP geometry), value in kcal/mol. [d]Energy difference for molecule in the crystal (geometry optimized in the crystal), value in kcal/mol. [e]Crystal packing effect defined as $\Delta E_{\rm cryst} - \Delta E_{\rm vac}$, value in kcal/mol.

S3.2.3 Case 2: Spin-Forbidden d–d Transitions in Solid State (B1–B4, C1–C4)

In this case, the $\delta_{\rm env}$ correction was obtained as the difference between two vertical excitation energies: one for the cluster model of a given TM complex in the crystal ($\Delta E_{\rm ve}^{\rm cryst}$), another one for isolated TM complex in vacuum ($\Delta E_{\rm ve}^{\rm vac}$). The COSMO/PBE0-D3(BJ)/def2-TZVP ground-state geometry was adopted for isolated TM complex in vacuum consistently with the same choice of geometry in further single-point DFT and WFT calculations of the benchmark study. Construction of the cluster model is described below.

Both excitation energies needed to determine the environmental correction were computed at the CASPT2 level using the cT(D)-DK basis set with the active space choice and other details described in Section S2.2. The underlying CASSCF calculations were state-specific for the ground state, but state-averaged over all three (degenerate or quasi-degenerate) components of the excited state ($^3T_{1g}$ or $^4T_{1g}$ under the idealized O_h symmetry). The average energy of the three components of the excited state was used to calculate the vertical energy for the crystal model, i.e. $\Delta E_{\mathrm{ve}}^{\mathrm{cryst}}(\mathrm{mean})$ to comply with the fact of observing in the experiment a single broad band with no fine structure (even if the calculations usually predict non-zero spread in the set of the three lowest vertical excitation energies). For isolated TM complex in vacuum, the lowest-energy component of the excited state was chosen, i.e., $\Delta E_{\mathrm{ve}}^{\mathrm{vac}}(\mathrm{lowest})$, to comply with the same choice made in the benchmarked WFT/DFT calculations. Thus, the working equation to define the environmental correction for d–d transition in solid state reads:

$$\delta_{\mathrm{env}}^{(\mathrm{case\ 2})} = \Delta E_{\mathrm{ve}}^{\mathrm{cryst}}(\mathrm{mean}) - \Delta E_{\mathrm{ve}}^{\mathrm{vac}}(\mathrm{lowest}). \tag{S.14}$$

See scheme in Figure S24 for graphical illustration of this definition. Note that the sign-convention defined in the main article applies to both vertical energy differences being subtracted (they are both negative for complexes **C1–C4**; i.e., the actual excitation energy is $-\Delta E_{\mathrm{ve}}^{\mathrm{cryst/vac}}$).

The cluster model comprising a single TM complex with the nearest-neighbor counterions (for ionic complexes) and hydrogen-bonded water molecules (for **C2–C4**) was constructed based on the experimental X-ray crystal structure of the actual compound for which the reflectance spectrum has been measured. The crystal structure used for **C3** is the one reported in this study (Section S1.2); for other complexes the literature crystal structures were used. See Table S12 for the details of the cluster models, including refcodes of their parent crystal structures. Structures of the cluster models used are also shown in Figures S25–S28, whereas their atomic coordinates are available in the ioChem-BD collection at `https://doi.org/10.19061/iochem-bd-7-8`. For non-ionic complexes **B4** and **C1**, the cluster model is limited to single TM complex molecule constrained to

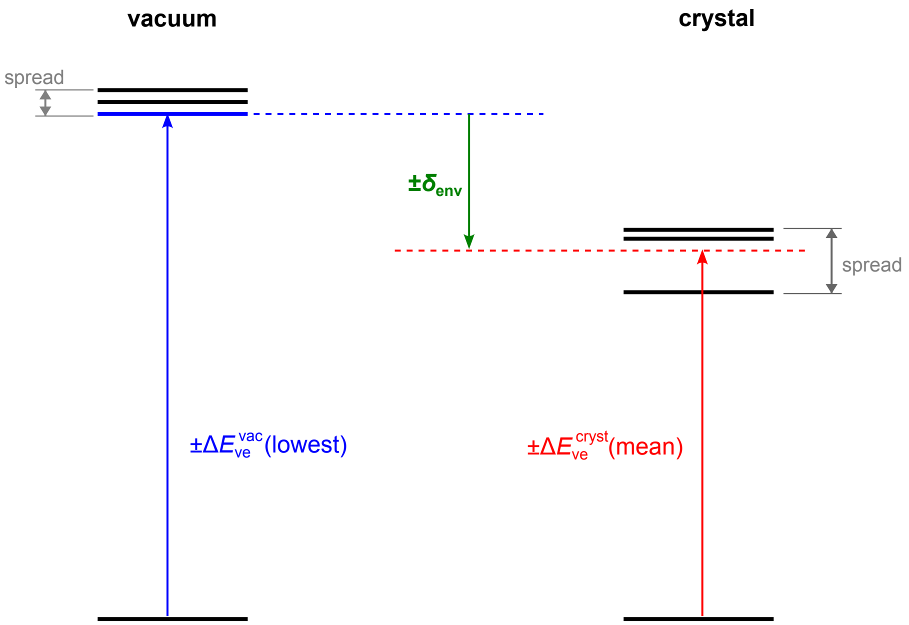

Figure S24: Scheme to illustrate definition of the environmental correction for d–d transition in crystal, eq. (S.14). The sign + or − before the δ_{env} and $\Delta E_{ve}^{vac/cryst}$ terms applies to the case of LS or HS ground state, respectively, due to the adopted sign convention for energy differences.

its experimental crystalline geometry.

When constructing cluster models, the crystallographic A – H bond lengths (where A is C, N, O) were increased by 10% to make them more realistic. (This procedure considerably affects total energies, but has only a minor effect on the excitation energies.) The Ewald potential of the ionic lattice was included by adding a finite set of point that numerically reproduces the Ewald potential in the cluster region using the method of Derenzo et al.[S94] The Ewald potential was determined under the point-charge approximation of all ions in the lattice (for TM complexes as well as NH_4^+ and SO_4^{2-} ions, the total ionic charge was placed in the position of the central atom).

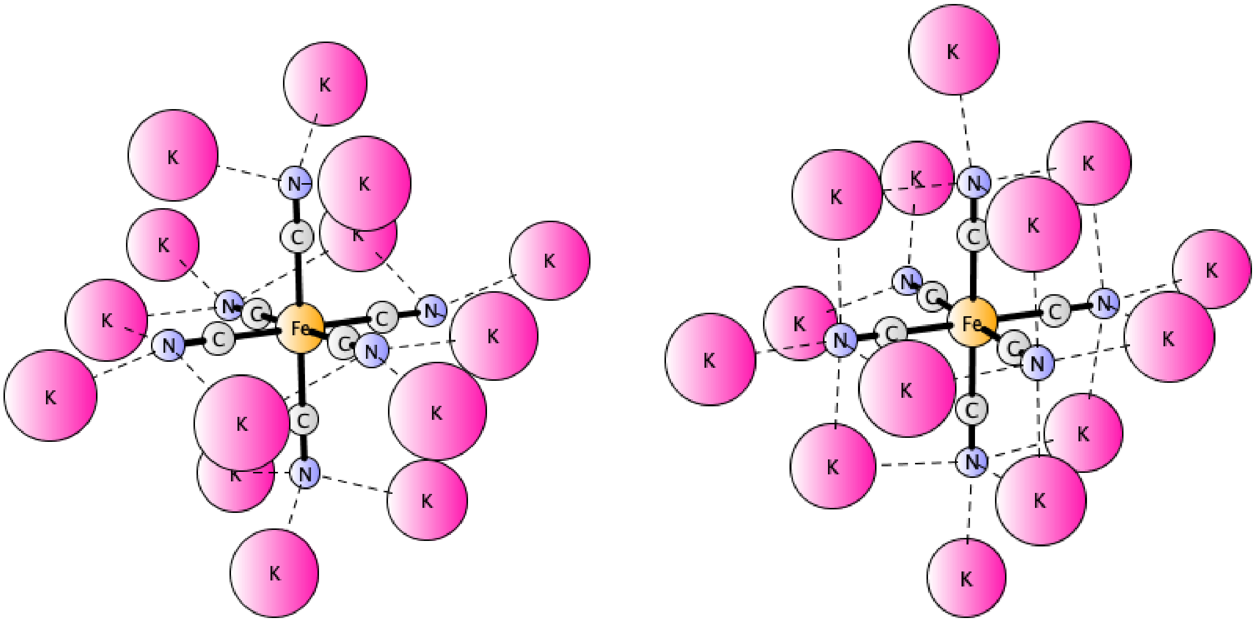

Figure S25: Cluster models: $\{K_{14}[Fe(CN)_6]\}^{11+}$ (**B1**) and $\{K_{14}[Fe(CN)_6]\}^{10+}$ (**B2**).

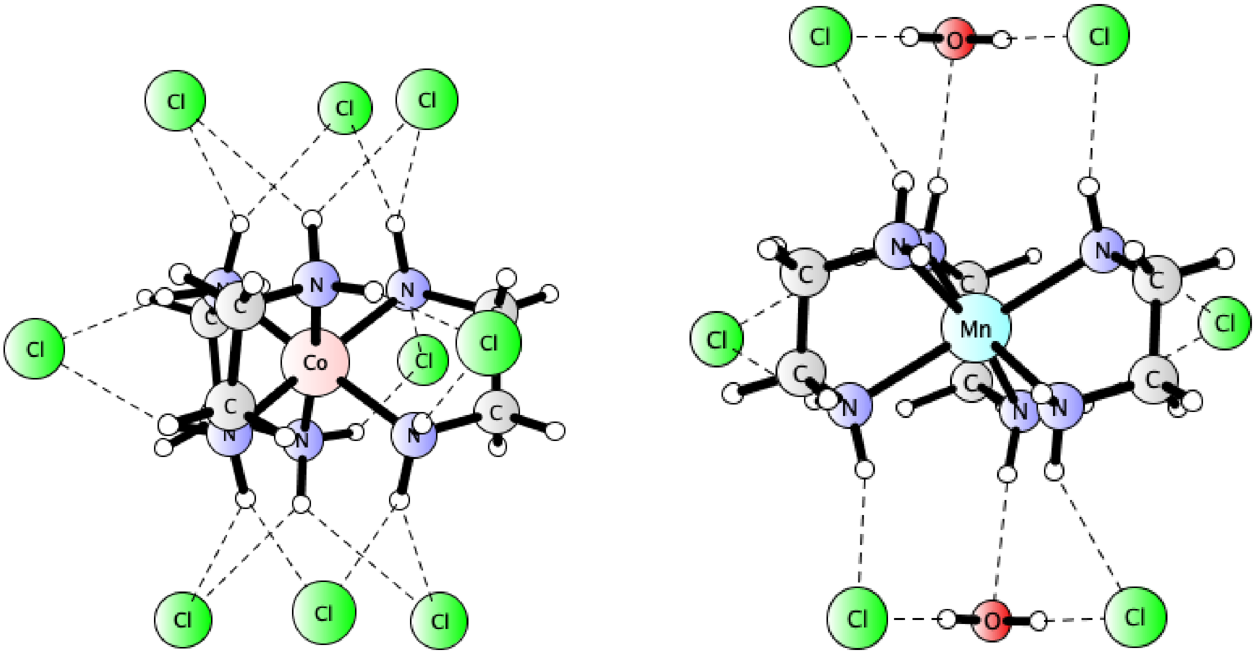

Figure S26: Cluster models: $\{[Co(en)_3]Cl_9\}^{6-}$ (**B3**) and $\{[Mn(en)_3]Cl_6 \cdot 2\,H_2O\}^{4-}$ (**C3**).

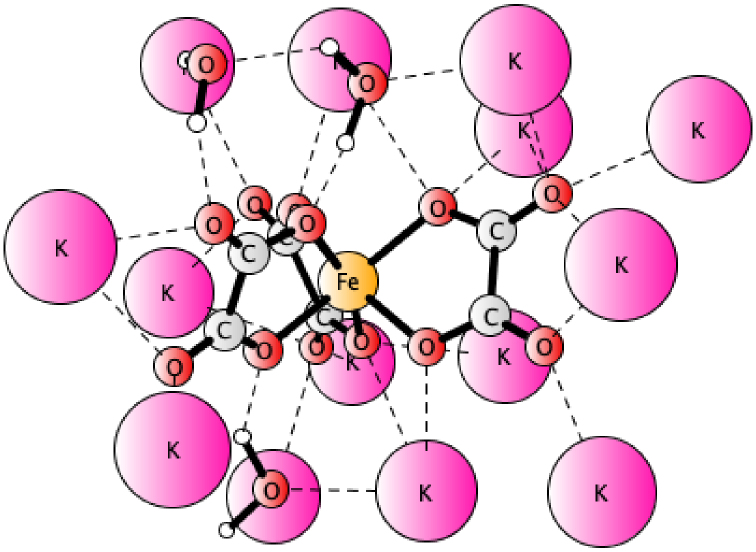

Figure S27: Cluster model $\{K_{14}[Fe(ox)_3] \cdot 3\,H_2O\}^{11+}$ (**C2**).

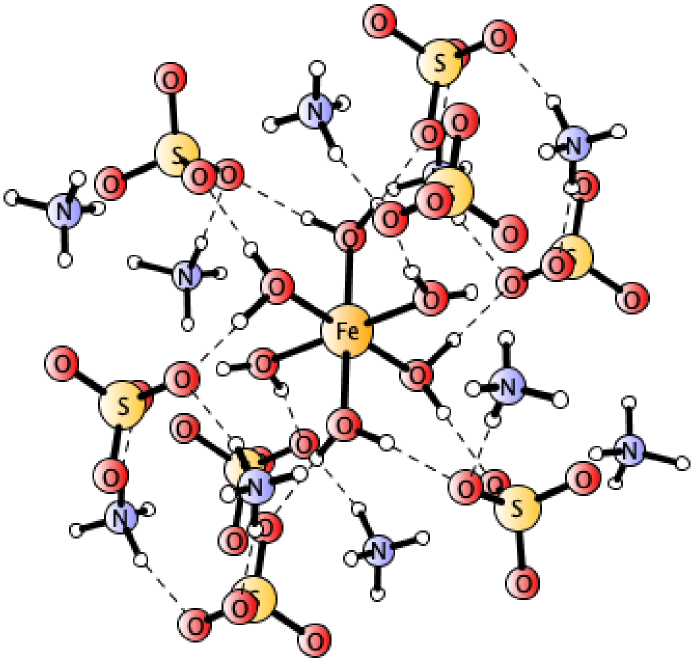

Figure S28: Cluster model $\{[Fe(H_2O)_6](SO_4)_8(NH_4)_{10}\}^{4-}$ (**C4**).

Table S11: Singlet–Triplet Vertical Excitation Energy of $[Fe(CN)_6]^{4-}$ Calculated for Different Models and Geometries. [a,b]

model	geometry	Fe – C [b]	C – N [b]	ΔE_{ve} [c]	spread [d]
$[Fe(CN)_6]^{4-}$	gaseous	1.986	1.174	49.1	0.0
	COSMO	1.912	1.168	69.5	0.0
	crystal	1.918 [f]	1.159 [g]	66.5	2.5
$\{K_{14}[Fe(CN)_6]\}^{10+}$ [e]	crystal	1.918 [f]	1.159 [g]	66.0	4.0

[a] Vertical excitation energies calculated at the CASPT2(10,10)/cT(D)-DK level for PBE0-D3(BJ)/def2-TZVP geometry (optimized in vacuum or within the COSMO model) or geometry of the appropriate model excised from the crystal structure (XUN-NAX). [b] Value in Å. [c] Average excitation energy (kcal/mol) for the three lowest triplets. [d] Energy difference (kcal/mol) between the highest and the lowest of the three lowest triplets. [e] Including Ewald potential of the infinite ionic lattice. [f] Average value (1.916, 1.918, 1.920 Å). [g] Average value (1.156, 1.160, 1.162 Å).

Table S12: Environmental Corrections for d–d Transitions in Crystals.

	compound	refcode/number[b]	cluster model	$\Delta E_{\mathrm{ve}}^{\mathrm{vac}}$ [c]			$\Delta E_{\mathrm{ve}}^{\mathrm{cryst}}$ [d]		δ_{env} [h]
				mean [e]	lowest[f]	spread [g]	mean [e]	spread [g]	
**B1**	$K_3[Fe(CN)_6]$	ICSD:60535[S95]	$\{K_{14}[Fe(CN)_6]\}^{11+}$	53.0	52.4	1.8	52.0	1.2	–0.4
**B2**	$K_4[Fe(CN)_6]\cdot 3\,H_2O$	XUNNAX[S96]	$\{K_{14}[Fe(CN)_6]\}^{10+}$	69.5	69.5	0.0	66.0	4.0	–3.5
**B3**	$[Co(en)_3]Cl_3$	IRIRAC[S97]	$\{[Co(en)_3]Cl_9\}^{6-}$	35.9	35.9	0.1	35.3	0.2	–0.6
**B4**	$[Co(acac)_3]$	COACAC03[S98]	$[Co(acac)_3]$	21.5	20.6	1.4	22.1	2.4	1.5
**C1**	$[Fe(acac)_3]$	FEACAC05[S99]	$[Fe(acac)_3]$	–35.7	–35.2	1.0	–33.4	0.5	1.9
**C2**	$K_3[Fe(ox)_3]\cdot 3\,H_2O$	KALGOU[S100]	$\{K_{14}[Fe(ox)_3]\cdot 3\,H_2O\}^{11+}$	–37.7	–37.6	0.4	–35.3	1.8	2.2
**C3**	$[Mn(en)_3]Cl_2\cdot H_2O$	2259710 [i]	$\{[Mn(en)_3]Cl_6\cdot 2\,H_2O\}^{4-}$	–50.8	–50.5	0.5	–50.5	3.6	0.0
**C4**	$[Fe(H_2O)_6](NH_4)_2(SO_4)_2$	ICSD:14346[S101]	$\{[Fe(H_2O)_6](SO_4)_8(NH_4)_{10}\}^{4-}$	–41.1	–40.3	1.5	–39.3	2.7	1.0

[a]Energy values in kcal/mol. [b]CSD refcode or deposition number, or ICSD number describing the experimental crystal structure. [c]Calculated for isolated molecule or ion in vacuum using COSMO/PBE0-D3(BJ)/def2-TZVP reference geometry. [d]Calculated for the cluster model build from the experimental crystal structure. [e]Averaged over sublevels of the excited state. [f]With respect to the lowest sublevel of the excited state. [g]Difference between the maximum and minimum energy sublevel of the excited state. [h]Environmental correction, eq. (S.14). [i]This work.

S3.3 Substituent Corrections for A6 and A7

With the aim of reducing computational cost in WFT calculations, **A6** and **A7** are simplified models of the actual complexes studied experimentally (**A6**′, **A7**′) in which some of the ligand substituents were replaced with H atoms (cf Figure 1 in main article). Note that models with full ligands (**A6**′, **A7**′) were used to determine the environmental correction; models with simplified ligands (**A6**, **A7**) were used to determine the vibrational corrections and in all further single-point energy calculations.

The effect of the side substituents on the spin–state splittings was determined as

$$\delta_{\text{subst}} = \Delta E_{\text{ad}}^{\text{full}} - \Delta E_{\text{ad}}^{\text{reduced}}. \tag{S.15}$$

(where "full" refers to **A6**′ or **A7**′, "reduced" refers to **A6** or **A7**) using DFT calculations in vacuum performed on top of the COSMO/PBE0-D3(BJ)/def2-TZVP geometries. With the def2-TZVP basis set, the PBE0-D3(BJ) and PBE-D3(BJ) methods were comparatively used to determine the substituent corrections (Table S13). As can be seen, the two functionals predict identical substituent effects to within 0.3 kcal/mol, and the mean value resulting from these calculations was used in the main article the best estimate of the δ_{subst} correction.

Table S13: Calculation of Substituent Corrections for **A6** and **A7**. [a]

	PBE0-D3(BJ)			PBE-D3(BJ)		
	$\Delta E_{\text{ad}}^{\text{full}}$ [b]	$\Delta E_{\text{ad}}^{\text{reduced}}$ [c]	δ_{subst} [d]	$\Delta E_{\text{ad}}^{\text{full}}$ [b]	$\Delta E_{\text{ad}}^{\text{reduced}}$ [c]	δ_{subst} [d]
**A6**	–6.8	–6.8	0.0	24.3	24.5	–0.2
**A7**	–4.6	–3.5	–1.1	18.1	18.9	–0.8

[a]Values in kcal/mol. [b]Spin–state splitting for full complexes, **A6**′ or **A7**′. [c]Spin–state splitting for reduced models, **A6** or **A7**. [d]Substituent effect, eq. (S.15), predicted by each functional; the δ_{subst} value used in the main article is the mean value from PBE0-D3(BJ) and PBE-D3(BJ) calculations.

S3.4 Method-Dependence of δ-Corrections

As discussed above, all the δ-corrections (vibrational, environmental, and substituent) are determined using approximate methods, i.e., DFT with a specific choice of functional or U parameter, or CASPT2 method. It is then clear that the resulting corrections have some method-dependence, which propagates to the reference values derived using these corrections. Whereas it is neither possible to calculate the corrections exactly, nor practical to calculate them using all possible approximate methods, one can probe method-dependence of the corrections by varying some critical parameters, such as the amount of exact exchange in DFT functional or the IPEA shift value in CASPT2, which are known to strongly influence the spin–state energetics. The appropriate considerations are detailed below, leading to general conclusions that with the change of method, the δ-corrections determined above vary by 1 kcal/mol or less, i.e., the effect is well within the error bar of 1–3 kcal/mol assumed for the reference values.

Vibrational corrections. For SCO complexes similar to or identical as those studied here, the method-dependence of the δ_{env} term has already been discussed in our recent Perspective (ref S76) by comparing vibrational enthalpy corrections calculated from harmonic frequencies computed using two functionals: PBE0 (25 % exact exchange) and PBE (0 % exact exchange). Although the two functionals yield very different spin–state splittings (due to the well effect of exact exchange), they lead to vibrational corrections identical to within 0.5 kcal/mol.[S76]

Substituent corrections. The sensitivity of the δ_{subst} term to the choice of functional has been already discussed above by comparing the PBE0-D3(BJ) and PBE-D3(BJ) results (cf Table S13). The resulting δ_{subst} values agree to better than 0.5 kcal/mol.

Environmental corrections – SCO complexes in solutions. For testing method-dependence, we considered the effect of switching from PBE0-D3(BJ) to PBE-D3(BJ). The latter functional was applied in single-point calculations on top of PBE0-D3(BJ) geometries (Table S14. Although the change of functional drastically affects the $\Delta E_{\text{ad}}^{\text{vac}}$ and $\Delta E_{\text{ad}}^{\text{solv}}$ values, the δ_{env} corrections vary

by no more than 0.7 kcal/mol).

Table S14: Environmental Corrections for SCO Complexes in Solution Caculated Using PBE0-D3(BJ) (default) and PBE-D3(BJ).

cmplx	solvent	method	$\Delta E_{\mathrm{ad}}^{\mathrm{vac}}$	$\Delta E_{\mathrm{ad}}^{\mathrm{solv}}$	δ_{env}
**A1**	DCM	PBE-D3(BJ)	16.0	17.0	1.0
		PBE0-D3(BJ)	−6.7	−6.0	0.7
	MeCN	PBE-D3(BJ)	16.0	17.7	1.7
		PBE0-D3(BJ)	−6.7	−5.7	1.0
	MeOH	PBE-D3(BJ)	16.0	17.8	1.7
		PBE0-D3(BJ)	−6.7	−5.7	1.0
	THF	PBE-D3(BJ)	16.0	17.8	1.7
		PBE0-D3(BJ)	−6.7	−5.7	1.0
	acetone	PBE-D3(BJ)	16.0	17.5	1.5
		PBE0-D3(BJ)	−6.7	−5.8	0.9
**A2**	CHCl3	PBE-D3(BJ)	28.3	28.1	−0.2
		PBE0-D3(BJ)	−1.8	−2.0	−0.2
**A4**	DMF	PBE-D3(BJ)	16.3	18.0	1.7
		PBE0-D3(BJ)	−7.2	−4.7	2.4
	MeCN	PBE-D3(BJ)	16.3	17.4	1.1
		PBE0-D3(BJ)	−7.2	−5.4	1.8
	water	PBE-D3(BJ)	16.3	18.2	1.9
		PBE0-D3(BJ)	−7.2	−4.8	2.4
**A5**	acetone	PBE-D3(BJ)	24.4	26.5	2.1
		PBE0-D3(BJ)	−6.6	−4.6	2.0
**A7**	acetone	PBE-D3(BJ)	18.1	19.1	0.9
		PBE0-D3(BJ)	−4.6	−3.5	1.1
**A8**	toluene	PBE-D3(BJ)	4.5	4.3	−0.1
		PBE0-D3(BJ)	−5.9	−6.1	−0.2
**A9**	toluene	PBE-D3(BJ)	21.1	21.7	0.6
		PBE0-D3(BJ)	−9.2	−9.1	0.2

[a]Values in kcal/mol; PBE-D3(BJ) refer to single-point results for PBE0-D3(BJ) geometries.

Environmental corrections – SCO complexes in crystals. We considered the effect of choosing different U values in the single-point DFT+U calculations used to determine the correction (see above). We compared the following U values: 2.00 eV, 2.65 eV and the one optimized to reproduce the experimental estimate of the adiabatic energy difference in the crystal (Table S15). Although

the ΔE_{ad}^{vac} and ΔE_{ad}^{cryst} quantities are sensitive to the choice of U, somewhat analogously to the effect of exact exchange in the previous case, the resulting δ_{env} values are nearly insensitive to U, with observed variations smaller than 0.5 kcal/mol.

Table S15: Environmental Corrections for SCO Complexes in Crystal Calculated Using DFT+U with Different U Values

cmplx	U	ΔE_{ad}^{vac}	ΔE_{ad}^{cryst}	δ_{env}
**A3**	2.00	8.7	8.3	–0.3
	2.37	5.3	4.8	–0.5
	2.65	2.8	2.2	–0.6
**A5**	2.00	6.8	6.5	–0.3
	2.13	5.5	5.2	–0.4
	2.65	0.7	0.1	–0.6
**A6**	2.00	5.4	5.4	0.0
	2.09	4.6	4.6	0.0
	2.65	–0.4	–0.5	–0.1
**A7**	2.00	10.2	11.7	1.5
	2.65	7.1	8.5	1.4
	3.70	2.1	3.4	1.3

[a]Values in kcal/mol, except U (eV).

Environmental corrections – Vertical excitations in crystals. We considered the effect of changing the IPEA shift parameter in CASPT2 calculations used to determine the correction: from the default value of the 0.25 au, to alternative values of 0 and 0.5 au (Table S16). It can be seen that the IPEA parameter considerably influences the ΔE_{ve}^{vac} and Δ_{ve}^{cryst} energy differences, but the resulting δ_{env} value deviates from the one determined at the IPEA shift of 0.25 a.u. within 1 kcal/mol.

Table S16: Environmental Corrections for d–d Transitions Calculated Using CASPT2 with Different IPEA Shift Values

cmplx	IPEA	$\Delta E_{\text{ve}}^{\text{vac}}$	$\Delta E_{\text{ve}}^{\text{cryst}}$	δ_{env}
**B1**	0.00	47.3	46.8	−0.5
	0.25	52.4	52.0	−0.4
	0.50	56.5	56.2	−0.3
**B2**	0.00	63.9	59.4	−4.5
	0.25	69.5	66.0	−3.5
	0.50	74.1	71.5	−2.6
**B3**	0.00	29.5	29.0	−0.5
	0.25	35.9	35.3	−0.6
	0.50	40.6	40.4	−0.2
**B4**	0.00	13.8	16.2	2.4
	0.25	20.6	22.1	1.5
	0.50	26.1	26.8	0.7
**C1**	0.00	−36.3	−34.3	1.9
	0.25	−35.2	−33.4	1.9
	0.50	−34.7	−33.0	1.7
**C2**	0.00	−38.4	−36.3	2.1
	0.25	−37.6	−35.3	2.2
	0.50	−36.9	−35.0	1.9
**C3**	0.00	−50.9	−51.6	−0.8
	0.25	−50.5	−50.5	0.0
	0.50	−50.2	−49.5	0.7
**C4**	0.00	−40.9	−40.8	0.1
	0.25	−40.3	−39.3	1.0
	0.50	−39.7	−38.0	1.7

[a]Values in kcal/mol, except IPEA given in a.u.

S4 Numeric Data for WFT and DFT Methods

S4.1 Deviations from Reference Values

Below, Table S17 and S18 give numeric deviations of calculated spin–state energetics from the reference values. Based on individual deviations observed for complexes **A1–A9**, **B1–B4**, **C1–C4**

$$d_i = \Delta E_{\text{calcd},\,i} - \Delta E_{\text{ref},\,i} \tag{S.16}$$

($i = 1, \ldots, N$, where $N = 17$), the error statistics of each method were computed as follows:

$|$**Max**$|$ largest absolute error

$$|\text{Max}| = \max_i |d_i| \tag{S.17}$$

ME mean error

$$\text{ME} = \frac{1}{N}\sum_i d_i \tag{S.18}$$

MAE mean absolute error

$$\text{MAE} = \frac{1}{N}\sum_i |d_i| \tag{S.19}$$

RMSD root mean square deviation

$$\text{RMSD} = \sqrt{\frac{1}{N}\sum_i d_i^2} \tag{S.20}$$

and are also included in the tables.

Table S17: Deviations of WFT Spin–State Energetic from Reference Values. [a]

	CCSD(T)	PBE0-CCSD(T)	PBE-CCSD(T)	CASPT2	CASPT2/CC	CASPT2+δMRCI	MRCI+Q(DSS)	MRCI+Q(PC)	MRCI+Q(RDC)	MRCI+Q(DC)	MP2
**A1**	–3.5	–1.7	0.1	–7.2	–2.6	0.3	–7.4	–8.1	–14.5	–17.4	–9.9
**A2**	1.0	3.9	6.6	–1.9	3.5	6.9	–6.1	–6.9	–15.7	–19.0	–2.5
**A3**	0.1	2.8	5.4	–4.5	1.1	4.5	–6.0	–6.8	–14.9	–18.0	–4.7
**A4**	0.1	2.0	3.6	–7.1	–1.2	1.9	–4.5	–5.2	–11.1	–13.7	–6.5
**A5**	–1.1	2.1	5.4	–5.2	0.0	3.3	–8.9	–9.7	–18.2	–21.4	–6.9
**A6**	–1.3	2.3	6.3	–4.3	4.4	3.6	–5.4	–6.0	–11.5	–13.8	–13.8
**A7**	–2.0	0.2	2.8	–5.3	–6.2	–1.1	–8.0	–8.5	–12.8	–14.7	–7.7
**A8**	2.1	1.7	2.3	4.3	5.0	10.6	0.5	0.3	–0.9	–1.6	–0.7
**A9**	–1.5	0.8	2.3	3.0	4.3	7.8	–2.0	–2.8	–6.0	–8.1	–1.2
**B1**	–3.4	–2.0	–1.4	–3.8	–2.3	–1.8	–3.4	–3.5	–4.3	–4.8	–1.7
**B2**	0.5	2.4	3.2	–0.1	1.8	4.2	0.4	0.3	–0.2	–0.4	3.0
**B3**	1.6	2.9	4.3	–2.8	0.3	0.7	0.3	0.2	–0.7	–1.2	2.8
**B4**	3.1	4.0	4.1	–1.2	2.5	1.4	2.6	2.5	1.1	0.6	2.1
**C1**	–0.5	–0.4	0.5	–6.0	–3.7	–1.1	–5.2	–5.7	–9.8	–11.6	–14.0
**C2**	–0.5	–0.2	0.4	–5.1	–2.7	–0.3	–3.7	–4.2	–7.8	–9.5	–12.4
**C3**	–1.3	–0.9	–0.5	–7.3	–3.3	–1.5	–2.4	–2.7	–4.8	–5.8	–7.7
**C4**	1.5	1.9	2.1	–0.9	1.8	0.4	3.2	3.0	2.3	1.8	–4.2
|Max| [b]	–3.5	4.0	6.6	–7.3	–6.2	10.6	–8.9	–9.7	–18.2	–21.4	–14.0
ME [c]	–0.3	1.3	2.8	–3.3	0.2	2.3	–3.3	–3.8	–7.6	–9.3	–5.1
MAE [d]	1.5	1.9	3.0	4.1	2.7	3.0	4.1	4.5	8.0	9.6	6.0
RMSD [e]	1.8	2.2	3.6	4.7	3.2	4.2	4.9	5.3	9.9	11.9	7.4

[a] Values in kcal/mol. [b] Largest absolute error. [c] Mean error. [d] Mean absolute error. [e] Root mean square error.

Table S18: Deviations of DFT Spin–State Energetic from Reference Values. [a]

	PWPB95-D3(BJ)	B2PLYP-D3(BJ)	LH14t-calPBE-D3	M06L-D3	LC-ωPBE-D3(BJ)	ωB97X-V	SSB-D	DSD-PBEB95-D3(BJ)	ωB97M-V	B97-D3	ωB97X-D
**A1**	−1.4	−3.4	0.5	−12.9	2.0	0.7	−7.3	−7.7	0.8	−8.3	−2.6
**A2**	0.3	−1.0	1.0	3.8	0.8	3.1	2.9	−4.3	4.8	2.6	−2.0
**A3**	−0.3	−2.0	0.5	4.5	0.2	2.7	2.8	−5.1	4.5	1.9	−1.6
**A4**	−1.1	−4.7	−3.8	−3.5	−2.8	0.6	−3.9	−5.6	3.0	−6.1	−4.5
**A5**	−3.3	−3.2	−1.0	−0.2	−3.6	−1.3	0.1	−8.0	−0.1	0.8	−4.9
**A6**	−3.5	−2.7	−1.3	−3.3	−2.3	−4.3	1.6	−9.7	−2.8	4.3	−5.5
**A7**	−3.5	−2.5	0.9	5.3	−0.7	−2.7	5.7	−7.3	−4.1	9.4	−4.0
**A8**	0.0	1.4	−1.7	−1.6	−1.6	−3.6	−2.4	−4.5	−4.7	−0.4	−5.0
**A9**	1.6	2.1	−0.2	0.1	2.4	0.5	11.9	−2.3	−1.6	−3.7	1.2
**B1**	−4.3	−0.4	−2.8	−2.6	−3.3	−5.4	−1.8	−5.5	−7.0	−2.6	−6.2
**B2**	−1.3	3.5	−2.5	−0.9	−3.7	−5.5	−3.2	−1.5	−5.9	−1.4	−6.0
**B3**	1.0	3.0	1.2	2.6	−1.6	−1.4	−0.6	0.1	0.2	2.4	−1.6
**B4**	2.6	3.3	2.7	4.6	0.6	1.0	0.6	1.4	3.1	2.5	0.8
**C1**	4.9	4.7	10.2	2.5	7.5	6.0	9.3	−1.4	5.1	10.8	6.9
**C2**	6.0	5.7	11.0	3.1	8.4	7.2	9.5	−0.2	6.3	11.2	8.1
**C3**	4.4	2.0	6.6	−0.4	7.0	9.5	2.6	0.0	9.0	5.3	9.9
**C4**	1.7	−1.9	4.7	2.6	7.2	7.6	−0.9	−4.0	8.4	0.5	7.4
|Max|[b]	6.0	5.7	11.0	−12.9	8.4	9.5	11.9	−9.7	9.0	11.2	9.9
ME[c]	0.2	0.2	1.5	0.2	1.0	0.9	1.6	−3.9	1.1	1.7	−0.6
MAE[d]	2.4	2.8	3.1	3.2	3.3	3.7	3.9	4.0	4.2	4.4	4.6
RMSD[e]	3.0	3.1	4.4	4.3	4.2	4.6	5.2	5.0	4.9	5.6	5.3

[a]Values in kcal/mol. [b]Largest absolute error. [c]Mean error. [d]Mean absolute error. [e]Root mean square deviation.

Table S18 (continued). [a]

	r2SCAN-D3(BJ)	LH20t-D4	B3LYP*-D3(BJ)	B3LYP-D3(BJ)	PW6B95-D3(BJ)	CAM-B3LYP-D3(BJ)	DSD-PBEP86-D3(BJ)	OLYP-D3(BJ)	OPBE-D3(BJ)	S12g	MN15
**A1**	1.3	3.8	0.7	−4.2	−4.2	−3.4	−10.2	−4.4	−2.9	−1.0	4.9
**A2**	11.6	6.2	5.3	−1.8	−2.3	−3.8	−6.7	8.6	13.3	7.5	0.9
**A3**	10.8	6.0	5.2	−1.6	−2.2	−3.3	−7.9	7.2	10.5	7.5	0.3
**A4**	2.9	3.8	0.0	−5.6	−3.5	−6.5	−9.5	−1.1	0.7	−2.3	−0.9
**A5**	7.5	1.9	2.6	−4.5	−6.7	−6.8	−9.9	5.3	8.9	7.2	−4.3
**A6**	4.0	0.5	4.3	−2.5	−6.2	−6.4	−12.0	11.0	13.4	8.1	−6.5
**A7**	6.5	0.4	4.1	−0.9	−4.0	−4.3	−8.3	12.8	15.7	10.5	5.5
**A8**	−1.5	−2.3	−2.1	−4.1	−5.2	−4.5	−4.6	0.3	1.4	−0.4	4.0
**A9**	2.3	5.5	−0.5	−7.6	−5.1	−7.7	−4.7	0.6	4.3	13.2	−0.7
**B1**	−0.3	−4.2	−3.1	−6.0	−9.6	−6.7	−4.1	−0.2	0.7	2.3	−8.4
**B2**	−0.3	−3.3	−3.3	−5.7	−8.3	−7.0	0.1	−0.2	1.5	−0.1	−11.2
**B3**	1.8	2.4	1.8	−0.3	−2.6	−1.9	0.1	3.4	2.0	2.4	−8.4
**B4**	3.6	3.9	3.5	1.9	0.0	0.6	1.5	3.8	2.4	3.4	−6.9
**C1**	8.2	9.8	13.1	10.1	6.6	6.5	−3.2	12.6	10.5	13.8	12.8
**C2**	8.5	10.5	14.1	11.3	7.7	7.7	−2.0	12.9	10.4	13.7	14.0
**C3**	3.1	11.5	12.8	10.5	7.8	8.6	−2.2	6.8	1.7	7.5	12.6
**C4**	6.6	9.8	9.8	8.2	6.1	6.9	−6.2	2.7	−1.8	3.7	8.6
|Max|[b]	11.6	11.5	14.1	11.3	−9.6	8.6	−12.0	12.9	15.7	13.8	14.0
ME[c]	4.5	3.9	4.0	−0.2	−1.9	−1.9	−5.3	4.8	5.4	5.7	1.0
MAE[d]	4.8	5.1	5.1	5.1	5.2	5.4	5.5	5.5	6.0	6.1	6.5
RMSD[e]	5.9	6.1	6.7	6.1	5.7	5.9	6.6	7.1	7.9	7.6	7.8

[a]Values in kcal/mol. [b]Largest absolute error. [c]Mean error. [d]Mean absolute error. [e]Root mean square deviation.

Table S18 (continued). [a]

	MVS	PBE0-D3(BJ)	TPSSh-D3(BJ)	M06-D3	S12h	SCAN-D3(BJ)	MN15L	PBE-D3(BJ)	TPSS-D3(BJ)	MVSh
**A1**	−17.5	−8.9	8.0	−24.6	−17.7	10.0	−37.4	13.7	17.2	−34.7
**A2**	3.0	−7.0	11.5	−12.1	−16.9	22.9	−15.6	23.2	24.2	−20.3
**A3**	1.6	−7.2	10.9	−11.4	−16.4	22.2	−16.3	22.5	23.4	−20.8
**A4**	−6.9	−11.6	5.5	−16.2	−19.6	13.7	−19.0	12.8	15.9	−24.0
**A5**	−3.3	−10.3	8.5	−14.7	−18.7	18.0	−21.0	20.9	21.4	−26.7
**A6**	−11.2	−10.6	7.6	−17.4	−18.6	12.1	−27.4	20.9	20.6	−33.0
**A7**	3.7	−5.6	4.4	−2.3	−9.7	10.4	−3.7	16.8	13.9	−15.5
**A8**	−2.7	−6.0	−1.6	−0.5	−8.1	−0.1	0.1	1.9	1.2	−12.6
**A9**	−11.8	−10.3	10.7	−17.4	−14.2	9.0	−24.6	20.3	23.1	−36.7
**B1**	−8.7	−8.2	−0.6	−10.8	−10.2	3.4	−12.2	5.9	4.8	−20.3
**B2**	−3.3	−8.1	−1.3	−9.2	−9.6	1.1	−4.9	2.6	2.8	−14.7
**B3**	−5.3	−4.7	2.9	−5.3	−6.7	5.4	−3.8	6.5	7.5	−12.8
**B4**	−3.3	−2.4	4.2	−3.2	−4.3	8.4	−2.6	6.9	7.9	−9.5
**C1**	−3.6	3.6	14.1	−5.2	0.6	9.7	−11.9	20.4	20.9	−16.7
**C2**	−3.8	4.6	14.9	−3.8	1.7	10.3	−11.6	20.3	21.0	−15.9
**C3**	−11.3	2.9	12.0	−4.6	1.4	6.4	−15.7	16.6	17.6	−15.6
**C4**	−11.4	2.0	11.4	−1.5	−1.5	16.9	−9.5	12.0	15.4	−15.7
|Max|[b]	−17.5	−11.6	14.9	−24.6	−19.6	22.9	−37.4	23.2	24.2	−36.7
ME[c]	−5.6	−5.2	7.2	−9.4	−9.9	10.6	−13.9	14.4	15.2	−20.3
MAE[d]	6.6	6.7	7.7	9.4	10.3	10.6	14.0	14.4	15.2	20.3
RMSD[e]	7.9	7.3	8.9	11.6	12.3	12.4	16.9	16.0	16.9	21.8

[a]Values in kcal/mol. [b]Largest absolute error. [c]Mean error. [d]Mean absolute error. [e]Root mean square error.

Table S19: Deviations of DFT Spin–State Energetic from Reference Values for PWPB95 and TPSSh Functionals with D3(BJ) and D4 Dispersion Corrections. [a]

	PWPB95-D3(BJ)	PWPB95-D4	TPSSh-D3(BJ)	TPSSh-D4
**A1**	−1.4	0.6	8.0	11.4
**A2**	0.3	2.8	11.5	15.9
**A3**	−0.3	1.9	10.9	15.0
**A4**	−1.1	1.2	5.5	8.5
**A5**	−3.3	−1.4	8.5	12.6
**A6**	−3.5	−3.0	7.6	8.6
**A7**	−3.5	−3.2	4.4	4.9
**A8**	0.0	0.2	−1.6	−1.5
**A9**	1.6	2.2	10.7	11.1
|Max| [b]	−3.5	−3.2	11.5	15.9
ME [c]	−1.2	0.1	7.3	9.6
MAE [d]	1.7	1.8	7.6	9.9
RMSD [e]	2.2	2.1	8.2	10.9

[a]Values in kcal/mol. Only for adiabatic energies of SCO complexes **A1**–**A9** (vertical energies for other complexes are not affected by dispersion correction). [b]Largest absolute error. [c]Mean error. [d]Mean absolute error. [e]Root mean square error.

S4.2 Diagnostics of Nondynamic Correlation

Table S20 reports diagnostics of nondynamic correlation: T_1, D_1, D_2, M, %MC, %DCE(T), and B.

The historically oldest T_1 diagnostic[S102,S103] and related D_1 diagnostic[S104] measure the contribution of single excitations to the CCSD amplitudes. The D_2 diagnostic[S105] is a similar measure as D_1, but for the double excitations. The M diagnostic[S106] is based on CASSCF natural occupation numbers: it measures their deviations from the ideal values at the HF level: 2 for the highest doubly-occupied orbital, 0 for the lowest unoccupied one, and 1 for singly-occupied orbitals. The %MC diagnostic (percentage of multiconfigurational character) is defined as

$$\%\mathrm{MC} = (1 - C_0^2) \times 100\%, \tag{S.21}$$

where C_0 is the CI coefficient of the leading configuration in CASSCF.

The B diagnostic, similar to the B_1 diagnostic of Truhlar and co-workers,[S107] is defined as

$$B = \Delta E^{\mathrm{PBE}} - \Delta E^{\mathrm{PBE0}}, \tag{S.22}$$

where ΔE^{PBE} is the energy difference calculated using pure functional PBE, ΔE^{PBE0} is the same energy difference calculated using the corresponding hybrid functional with 25% admixture of the exact exchange (EE); both energies in eq. (S.22) are in kcal/mol. The original B_1 was similarly based on the pair of functionals B1LYP and BLYP. Also note the study of Martin with co-workers[S108] exploring similar diagnostic A_λ. All these diagnostics measure the sensitivity of DFT energy difference to the admixed exact exchange, which is regarded as a good predictor of how the energetics are sensitive to the theory level in general.[S108]

Finally, the %DCE(T) diagnostic is percentage contribution of the triples energy to the differential correlation energy (DCE) at the CCSD(T) level, i.e.,

$$\%\mathrm{DCE(T)} = \frac{\Delta E^{\mathrm{(T)}}}{\Delta E^{\mathrm{CCSD(T)}} - \Delta E^{\mathrm{HF}}} \times 100\%. \tag{S.23}$$

In eq. (S.23), the numerator is the triples contribution to the energy difference between the spin states; the denominator is the DCE between the spin states at the CCSD(T) level. The %DCE(T) is defined analogously as the %TAE(T) diagnostic of Martin with co-workers,[S109,S110] which expresses the triples contribution to total atomization energy (TAE).

We sought for possible correlations between each of these diagnostics and either absolute or signed errors of the CCSD(T) spin–state energetics. The %DCE(T) and B diagnostics are defined per energy difference and thus can be directly plotted vis-a-vis the CCSD(T)'s errors. For each of the other diagnostics ($f = T_1$, D_1, D_2, M, %MC), which are defined per state, we considered either the average value

$$f^{\mathrm{avg}} = \tfrac{1}{2}\left(f^{\mathrm{state1}} + f^{\mathrm{state2}}\right), \tag{S.24}$$

the maximum value

$$f^{\max} = \max\left(f^{\text{state1}}, f^{\text{state2}}\right) \tag{S.25}$$

or the differential value

$$\Delta f = f^{\text{state2}} - f^{\text{state1}} \tag{S.26}$$

to be plotted vis-a-vis the CCSD(T)'s errors. The appropriate correlation plots are shown in Figures S29–S34, including also numeric values of the Pearson's and Spearman's correlation coefficients for each case.

Based on the results, we find no appreciable correlation between any of these diagnostics and the CCSD(T)'s errors. This is reflected in the values of correlation coefficients, which are low or very low, with the maximum values of 0.42 (Pearson) and 0.54 (Spearman).

Table S20: Diagnostics of Nondynamic Correlation

complex	mult	T_1	D_1	D_2	M	%MC	%DCE(T)	B
**A1**	2	0.027	0.155	0.178	0.045	6.6	16.2	22.5
	6	0.023	0.115	0.175	0.009	1.1		
**A2**	1	0.020	0.106	0.181	0.033	5.8	17.1	29.9
	5	0.014	0.046	0.180	0.022	1.8		
**A3**	1	0.020	0.104	0.181	0.033	5.7	16.9	29.5
	5	0.016	0.050	0.180	0.022	1.7		
**A4**	1	0.019	0.111	0.149	0.033	5.7	15.6	24.1
	5	0.012	0.057	0.147	0.022	1.8		
**A5**	1	0.022	0.111	0.187	0.034	5.9	17.8	31.1
	5	0.016	0.073	0.188	0.023	1.8		
**A6**	1	0.029	0.169	0.206	0.039	7.1	20.5	31.3
	5	0.017	0.109	0.204	0.025	2.0		
**A7**	2	0.021	0.144	0.198	0.038	5.0	18.7	22.3
	4	0.016	0.071	0.197	0.018	2.3		
**A8**	1	0.033	0.280	0.204	0.120	13.4	18.9	10.4
	3	0.027	0.184	0.186	0.075	9.6		
**A9**	2	0.035	0.149	0.185	0.074	10.9	16.5	30.7
	6	0.015	0.058	0.180	0.014	1.4		
**B1**	2	0.035	0.147	0.174	0.062	9.7	19.2	14.3
	4	0.030	0.149	0.174	0.064	6.6		
**B2**	1	0.040	0.156	0.176	0.047	9.9	20.8	12.9
	3	0.034	0.152	0.176	0.056	8.7		
**B3**	1	0.028	0.168	0.164	0.050	7.8	18.8	11.2
	3	0.026	0.187	0.171	0.087	8.7		
**B4**	1	0.028	0.178	0.164	0.042	6.9	19.7	9.3
	3	0.027	0.199	0.167	0.073	7.5		
**C1**	4	0.028	0.208	0.168	0.056	5.8	19.2	16.7
	6	0.022	0.093	0.165	0.008	0.9		
**C2**	4	0.029	0.200	0.174	0.057	5.9	18.0	15.5
	6	0.023	0.093	0.173	0.008	0.9		
**C3**	4	0.014	0.081	0.145	0.063	5.2	12.7	13.7
	6	0.011	0.036	0.143	0.011	1.0		
**C4**	3	0.015	0.063	0.126	0.374	22.8	13.6	7.2
	5	0.011	0.033	0.125	0.016	1.5		

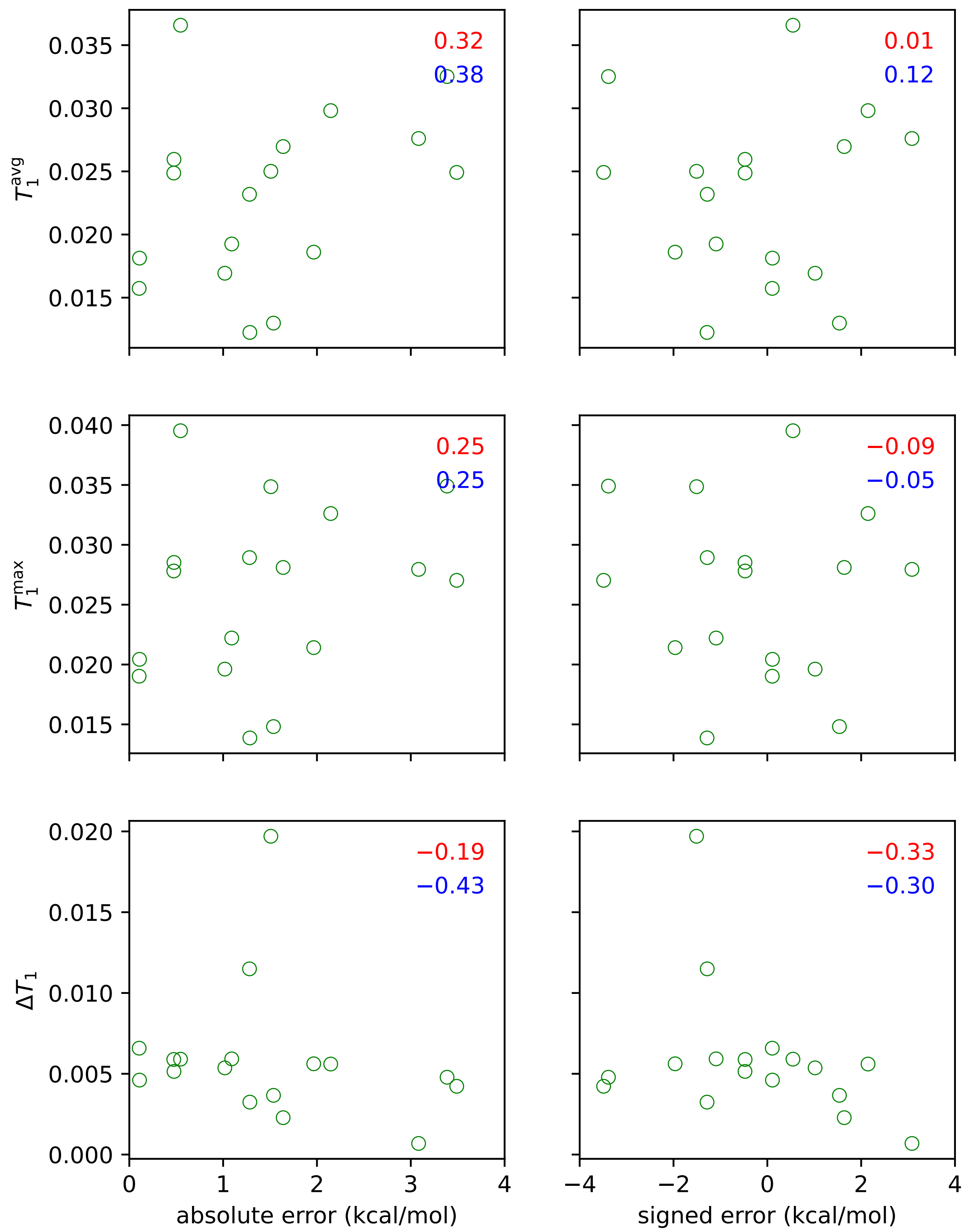

Figure S29: Relation between absolute and signed errors of the CCSD(T) spin–state splittings and the T_1 diagnostic. Annotated values are correlation coefficients of Pearson (red) and Spearman (blue).

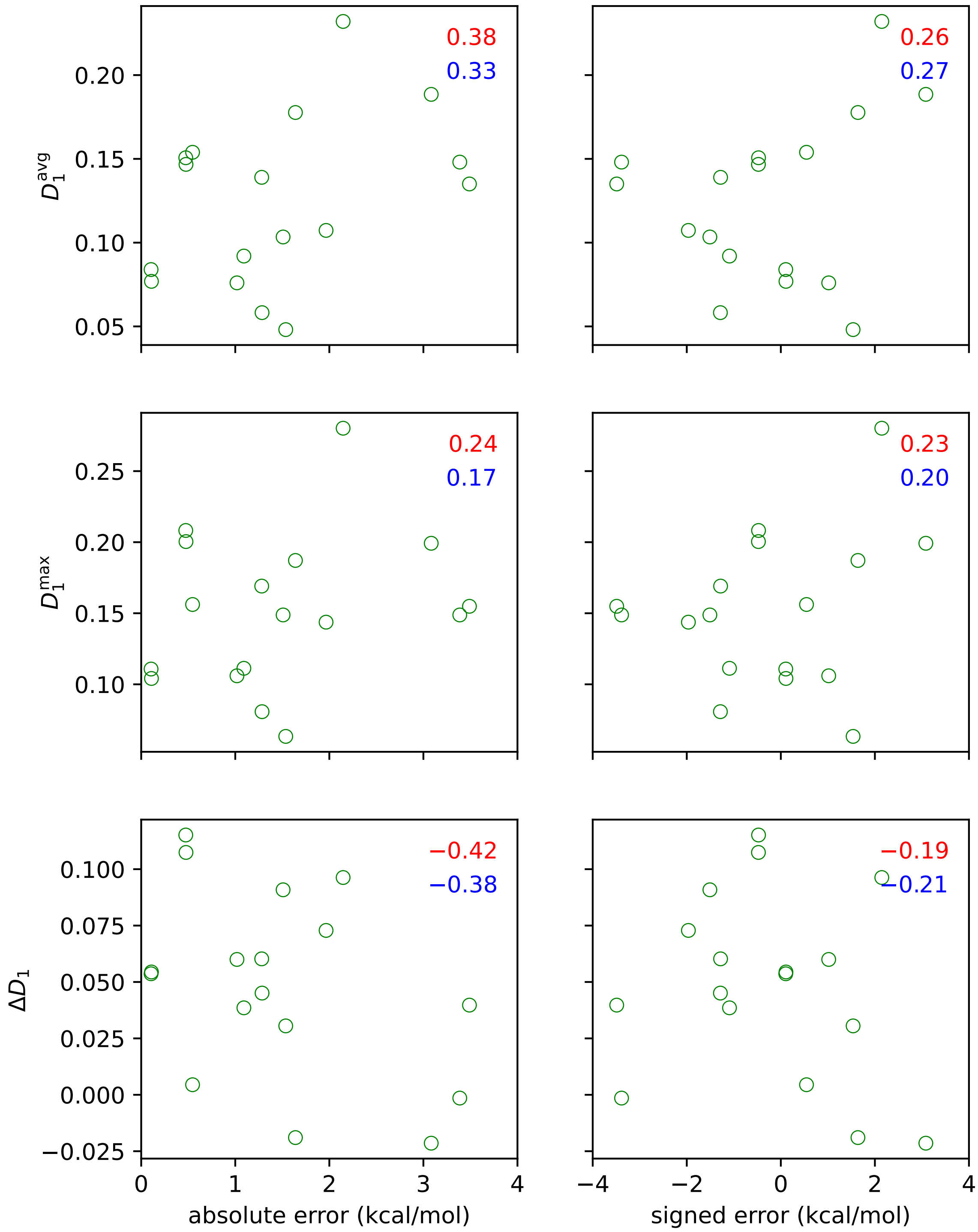

Figure S30: Relation between absolute and signed errors of the CCSD(T) spin–state splittings and the D_1 diagnostic. Annotated values are correlation coefficients of Pearson (red) and Spearman (blue).

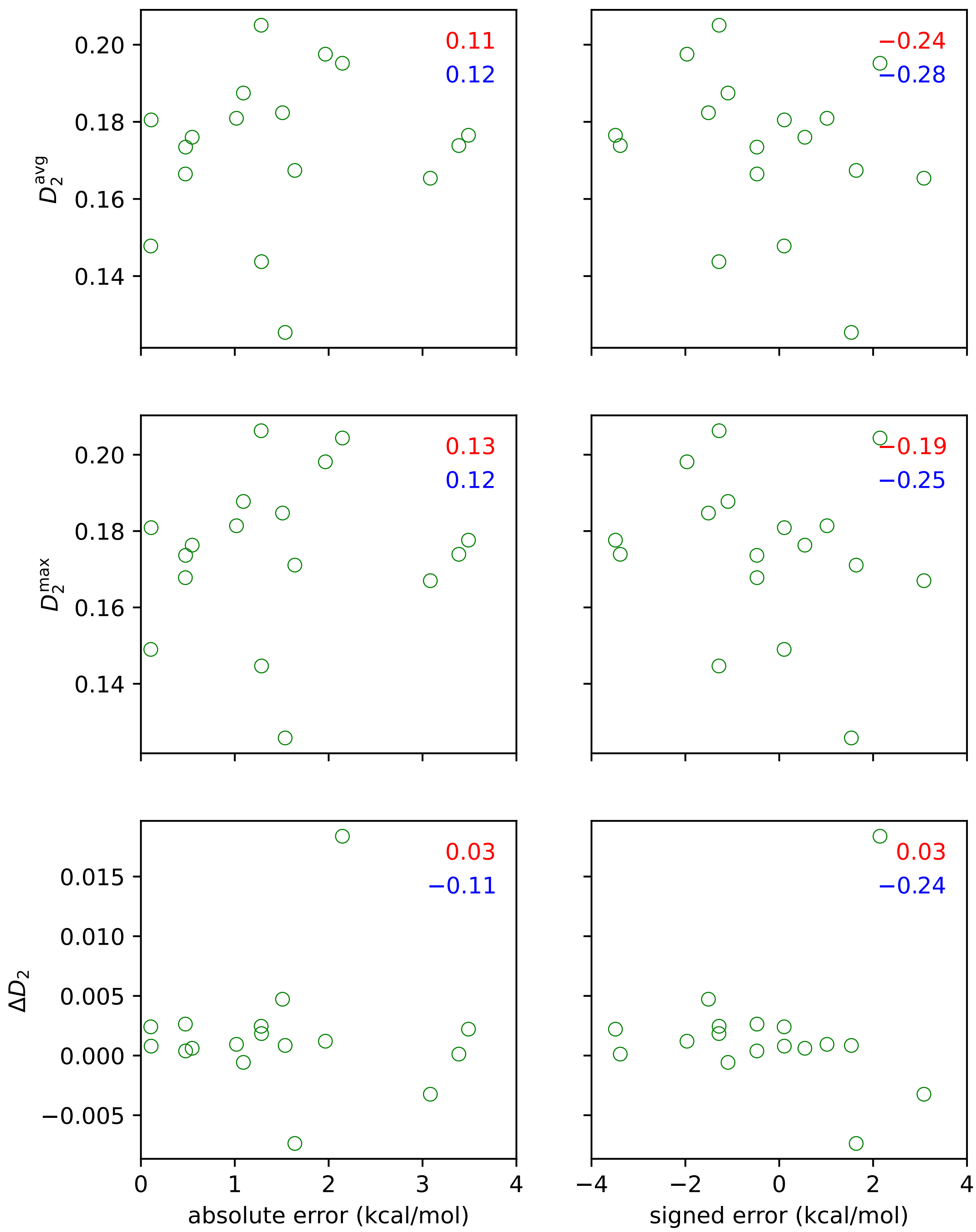

Figure S31: Relation between absolute and signed errors of the CCSD(T) spin–state splittings and the D_2 diagnostic. Annotated values are correlation coefficients of Pearson (red) and Spearman (blue).

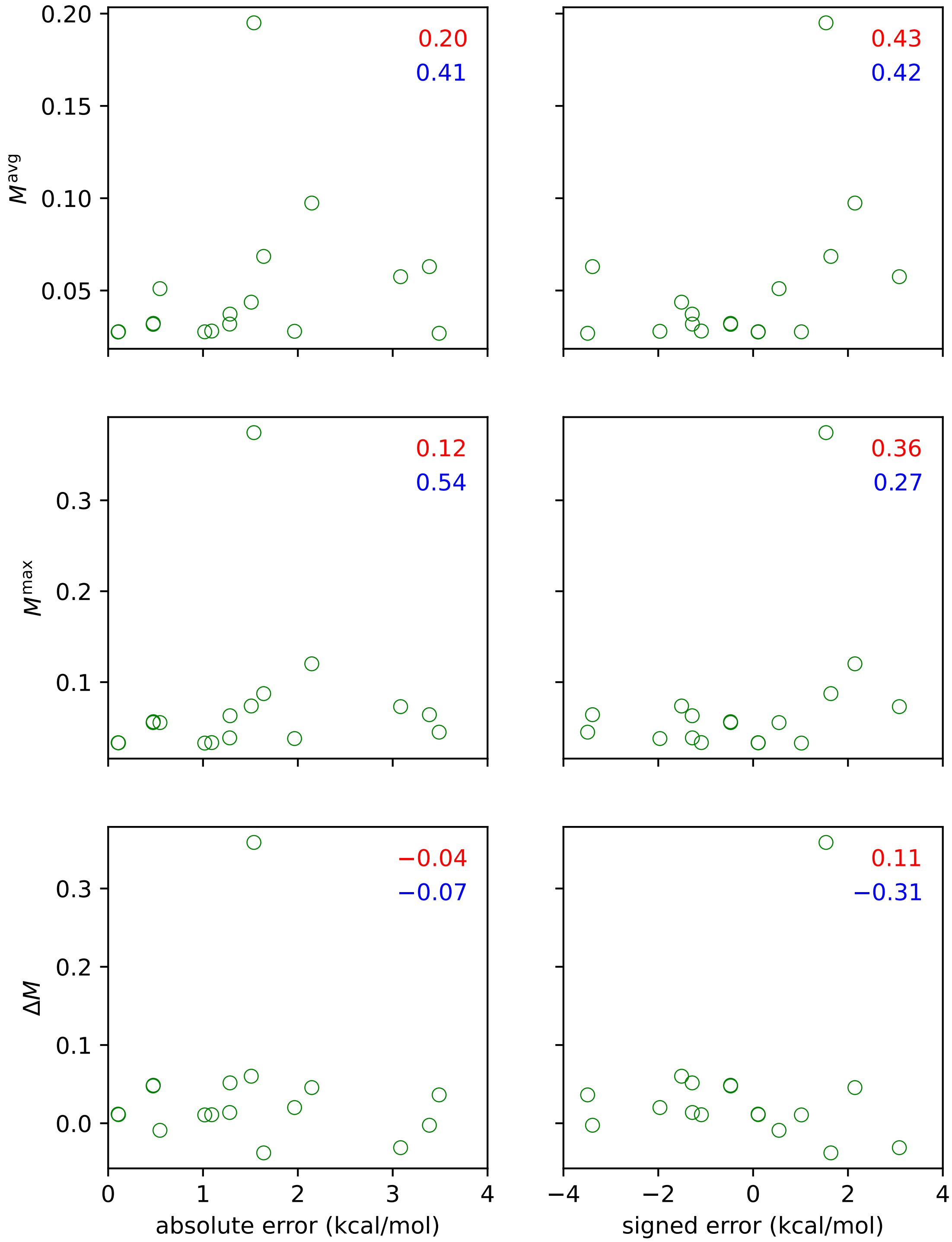

Figure S32: Relation between absolute and signed errors of the CCSD(T) spin–state splittings and the *M* diagnostic. Annotated values are correlation coefficients of Pearson (red) and Spearman (blue).

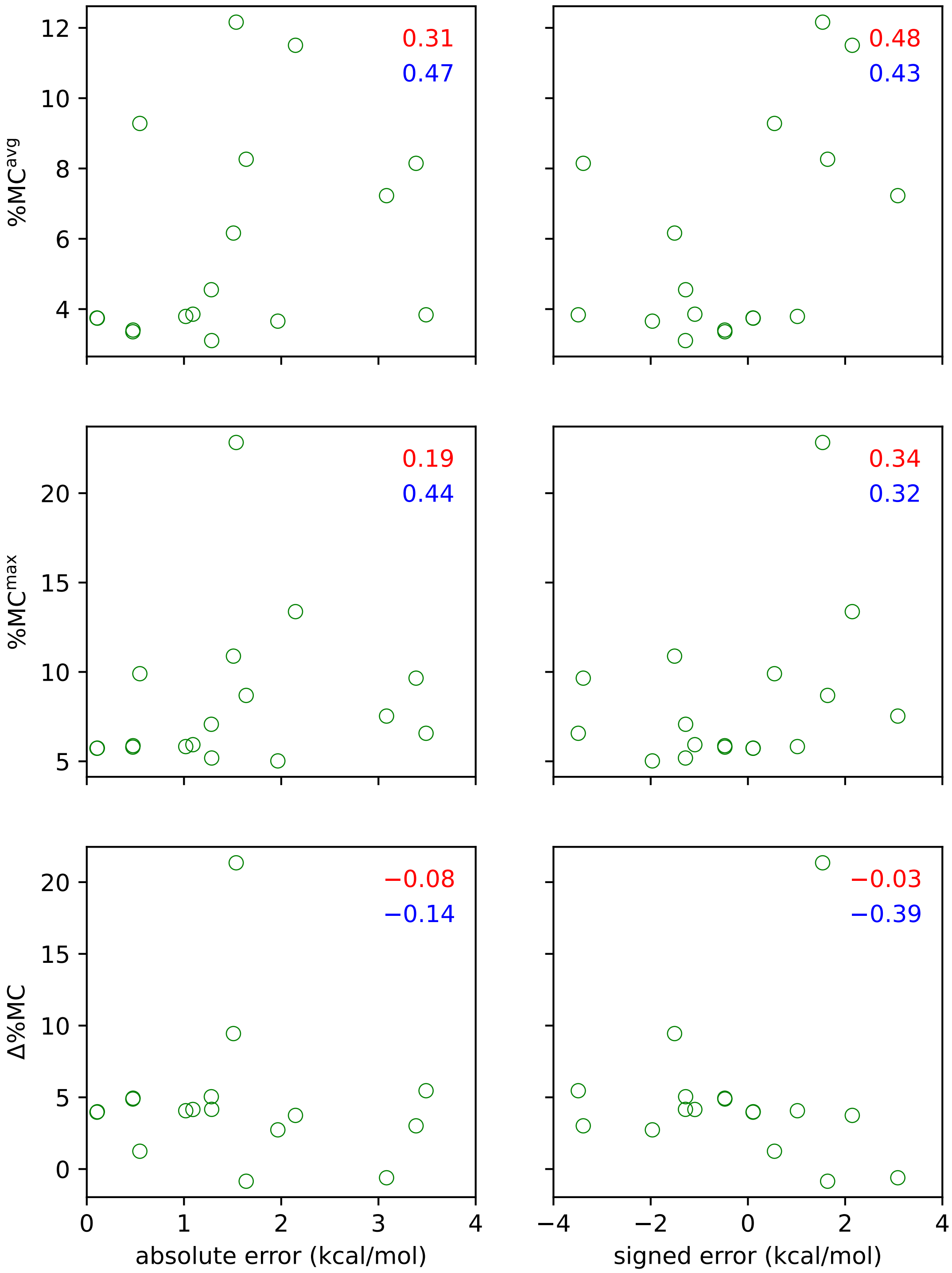

Figure S33: Relation between absolute and signed errors of the CCSD(T) spin–state splittings and the %MC diagnostic. Annotated values are correlation coefficients of Pearson (red) and Spearman (blue).

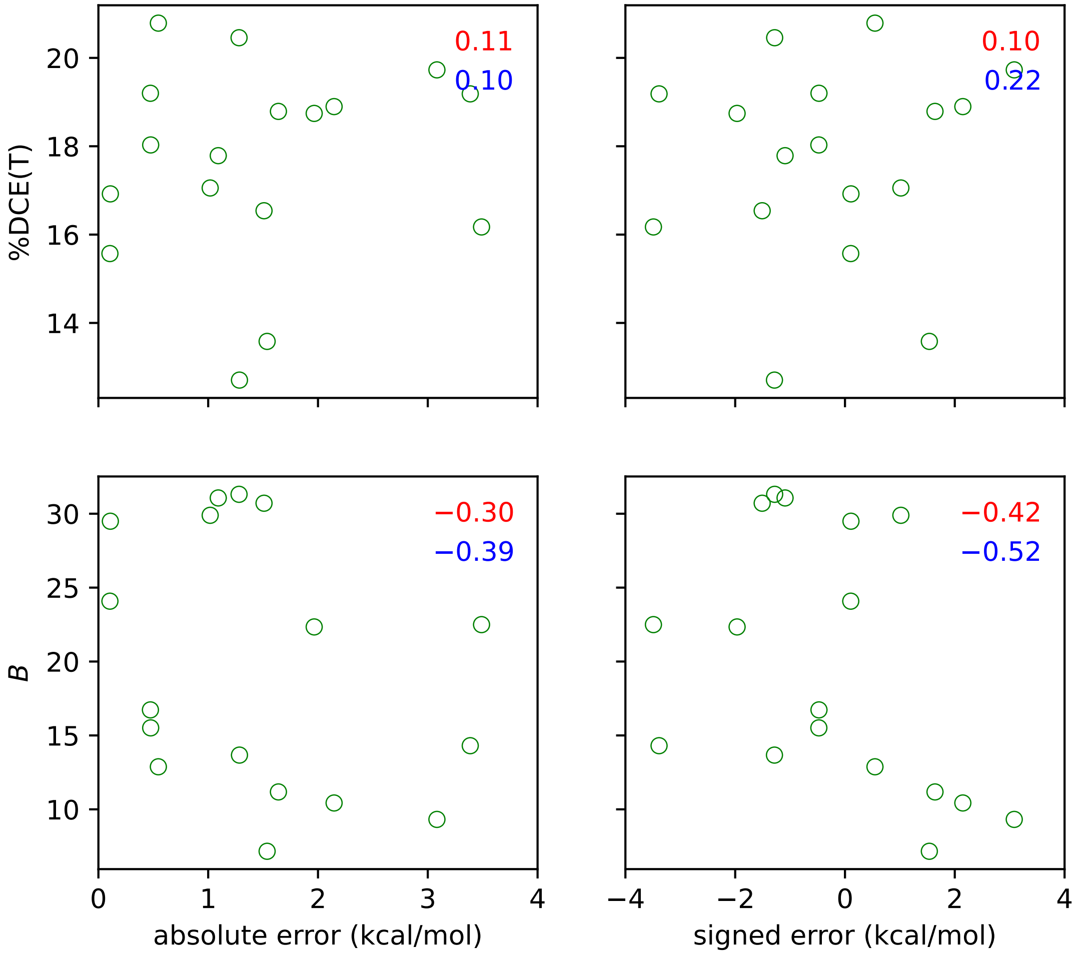

Figure S34: Relation between absolute and signed errors of the CCSD(T) spin–state splittings and the %DCE(T) and B diagnostics. Annotated values are correlation coefficients of Pearson (red) and Spearman (blue).

References

(S1) Kortüm, G. *Reflectance Spectroscopy Principles, Methods, Applications*; Springer-Verlag: New York, 1969.

(S2) Wojdyr, M. *Fityk*: a general-purpose peak fitting program. *J. Appl. Crystallogr.* **2010**, *43*, 1126–1128.

(S3) Schmidtke, H.-H.; Eyring, G. The Cation Effect on the Charge Transfer and Ligand Field Spectrum of the Complex Ferricyanide. *Zeitschrift für Physikalische Chemie* **1974**, *92*, 211–222.

(S4) Cotton, F. A.; Meyers, M. D. Magnetic and Spectral Properties of the Spin-Free $3d^6$ Systems Iron(II) and Cobalt(III) in Cobalt(III) Hexafluoride Ion: Probable Observation of Dynamic Jahn-Teller Effects. *J. Am. Chem. Soc.* **1960**, *82*, 5023–5026.

(S5) CrysAlis PRO, Rigaku OxfordDiffraction, Yarnton, England. 2015.

(S6) Sheldrick, G. M. Crystal Structure Refinement with SHELXL. *Acta Crystallogr., Sect. C: Struct. Chem.* **2015**, *71*, 3.

(S7) Sheldrick, G. M. SHELX2017, Programs for crystal structure determination, Universität Göttingen, Germany. 2017.

(S8) Putz, H.; Brandenburg, K. Diamond – Crystal and Molecular Structure Visualization, Crystal Impact, GbR, Kreuzherrenstr. 102, D-53227 Bonn.

(S9) Kepert, D. L. *Inorganic Stereochemistry*; Springer-Verlag Berlin, Heidelberg, 1982; pp 92–111.

(S10) Beattie, J. K. Conformational analysis of tris(ethylenediamine) complexes. *Acc. Chem. Res.* **1971**, *4*, 253–259.

(S11) Perdew, J. P.; Burke, K.; Ernzerhof, M. Generalized Gradient Approximation Made Simple. *Phys. Rev. Lett.* **1996**, *77*, 3865–3868.

(S12) Adamo, C.; Barone, V. Toward reliable density functional methods without adjustable parameters: The PBE0 model. *J. Chem. Phys.* **1999**, *110*, 6158–6170.

(S13) Zhao, Y.; Truhlar, D. G. The M06 Suite of Density Functionals for Main Group Thermochemistry, Thermochemical Kinetics, Noncovalent Interactions, Excited States, and Transition Elements: Two New Functionals and Systematic Testing of Four M06-class Functionals and 12 Other Functionals. *Theor. Chem. Acc.* **2008**, *120*, 215–241.

(S14) Tao, J.; Perdew, J. P.; Staroverov, V. N.; Scuseria, G. E. Climbing the Density Functional Ladder: Nonempirical Meta–Generalized Gradient Approximation Designed for Molecules and Solids. *Phys. Rev. Lett.* **2003**, *91*, 146401–146404.

(S15) Perdew, J. P.; Tao, J.; Staroverov, V. N.; Scuseria, G. E. Meta-generalized gradient approximation: Explanation of a realistic nonempirical density functional. *J. Chem. Phys.* **2004**, *120*, 6898–6911.

(S16) Yanai, T.; Tew, D. P.; Handy, N. C. A new hybrid exchange–correlation functional using the Coulomb-attenuating method (CAM-B3LYP). *Chem. Phys. Lett.* **2004**, *393*, 51 – 57.

(S17) Chai, J.-D.; Head-Gordon, M. Long-range corrected hybrid density functionals with damped atom-atom dispersion corrections. *Phys. Chem. Chem. Phys.* **2008**, *10*, 6615–6620.

(S18) Mardirossian, N.; Head-Gordon, M. ωB97X-V: A 10-parameter, range-separated hybrid, generalized gradient approximation density functional with nonlocal correlation, designed by a survival-of-the-fittest strategy. *Phys. Chem. Chem. Phys.* **2014**, *16*, 9904–9924.

(S19) Mardirossian, N.; Head-Gordon, M. ωB97M-V: A combinatorially optimized, range-

separated hybrid, meta-GGA density functional with VV10 nonlocal correlation. *J. Chem. Phys.* **2016**, *144*, 214110.

(S20) Arbuznikov, A. V.; Kaupp, M. Towards improved local hybrid functionals by calibration of exchange-energy densities. *J. Chem. Phys.* **2014**, *141*, 204101.

(S21) Haasler, M.; Maier, T. M.; Grotjahn, R.; Gückel, S.; Arbuznikov, A. V.; Kaupp, M. A Local Hybrid Functional with Wide Applicability and Good Balance between (De)Localization and Left-Right Correlation. *J. Chem. Theory Comput.* **2020**, *16*, 5645–5657.

(S22) Sun, J.; Ruzsinszky, A.; Perdew, J. P. Strongly Constrained and Appropriately Normed Semilocal Density Functional. *Phys. Rev. Lett.* **2015**, *115*, 036402.

(S23) Furness, J. W.; Kaplan, A. D.; Ning, J.; Perdew, J. P.; Sun, J. Accurate and Numerically Efficient r2SCAN Meta-Generalized Gradient Approximation. *J. Phys. Chem. Lett.* **2020**, *11*, 8208–8215.

(S24) Becke, A. D. Density-functional thermochemistry. III. The role of exact exchange. *J. Chem. Phys.* **1993**, *98*, 5648–5652.

(S25) Stephens, P. J.; Devlin, F. J.; Chabalowski, C. F.; Frisch, M. J. Ab Initio Calculation of Vibrational Absorption and Circular Dichroism Spectra Using Density Functional Force Fields. *J. Phys. Chem.* **1994**, *98*, 11623–11627.

(S26) Reiher, M.; Salomon, O.; Hess, B. A. Reparameterization of hybrid functionals based on energy differences of states of different multiplicity. *Theor. Chem. Acc.* **2001**, *107*, 48–55.

(S27) Handy, N. C.; Cohen, A. J. Left–Right Correlation Energy. *Mol. Phys.* **2001**, *99*, 403–412.

(S28) Grimme, S. Semiempirical GGA-type density functional constructed with a long-range dispersion correction. *J. Comp. Chem.* **2006**, *27*, 1787–1799.

(S29) Yu, H. S.; He, X.; Li, S. L.; Truhlar, D. G. MN15: A Kohn-Sham global-hybrid exchange–correlation density functional with broad accuracy for multi-reference and single-reference systems and noncovalent interactions. *Chem. Sci.* **2016**, *7*, 5032–5051.

(S30) Yu, H. S.; He, X.; Truhlar, D. G. MN15-L: A New Local Exchange–Correlation Functional for Kohn–Sham Density Functional Theory with Broad Accuracy for Atoms, Molecules, and Solids. *J. Chem. Theory Comput.* **2016**, *12*, 1280–1293.

(S31) Zhao, Y.; Truhlar, D. G. Design of Density Functionals That Are Broadly Accurate for Thermochemistry, Thermochemical Kinetics, and Nonbonded Interactions. *J. Phys. Chem. A* **2005**, *109*, 5656–5667.

(S32) Vydrov, O. A.; Scuseria, G. E. Assessment of a long-range corrected hybrid functional. *J. Chem. Phys.* **2006**, *125*, 234109.

(S33) Vydrov, O. A.; Heyd, J.; Krukau, A. V.; Scuseria, G. E. Importance of short-range versus long-range Hartree-Fock exchange for the performance of hybrid density functionals. *J. Chem. Phys.* **2006**, *125*, 074106.

(S34) Vydrov, O. A.; Scuseria, G. E.; Perdew, J. P. Tests of functionals for systems with fractional electron number. *J. Chem. Phys.* **2007**, *126*, 154109.

(S35) Swart, M.; Solá, M.; Bickelhaupt, F. M. A new all-round density functional based on spin states and SN2 barriers. *J. Chem. Phys.* **2009**, *131*, 094103.

(S36) Swart, M.; Solà, M.; Bickelhaupt, F. M. Switching between OPTX and PBE exchange functionals. *J. Comput. Methods Sci. Eng.* **2009**, *9*, 69–77.

(S37) Swart, M. A new family of hybrid density functionals. *Chem. Phys. Lett.* **2013**, *580*, 166–171.

(S38) Sun, J.; Perdew, J. P.; Ruzsinszky, A. Semilocal density functional obeying a strongly tightened bound for exchange. *Proc. Natl Acad. Sci.* **2015**, *112*, 685–689.

(S39) Grimme, S. Semiempirical hybrid density functional with perturbative second-order correlation. *J. Chem. Phys.* **2006**, *124*, 034108.

(S40) Goerigk, L.; Grimme, S. Efficient and Accurate Double-Hybrid-Meta-GGA Density Functionals—Evaluation with the Extended GMTKN30 Database for General Main Group Thermochemistry, Kinetics, and Noncovalent Interactions. *J. Chem. Theory Comput.* **2011**, *7*, 291–309.

(S41) Kozuch, S.; Martin, J. M. L. DSD-PBEP86: in search of the best double-hybrid DFT with spin-component scaled MP2 and dispersion corrections. *Phys. Chem. Chem. Phys.* **2011**, *13*, 20104–20107.

(S42) Kozuch, S.; Martin, J. M. L. Spin-component-scaled double hybrids: An extensive search for the best fifth-rung functionals blending DFT and perturbation theory. *J. Comput. Chem.* **2013**, *34*, 2327–2344.

(S43) Weigend, F.; Ahlrichs, R. Balanced basis sets of split valence, triple zeta valence and quadruple zeta valence quality for H to Rn: Design and assessment of accuracy. *Phys. Chem. Chem. Phys.* **2005**, *7*, 3297–3305.

(S44) de Jong, W. A.; Harrison, R. J.; Dixon, D. A. Parallel Douglas–Kroll energy and gradients in NWChem: Estimating scalar relativistic effects using Douglas–Kroll contracted basis sets. *J. Chem. Phys.* **2001**, *114*, 48–53.

(S45) Dunning, T. H. Gaussian basis sets for use in correlated molecular calculations. I. The atoms boron through neon and hydrogen. *J. Chem. Phys.* **1989**, *90*, 1007–1023.

(S46) Radoń, M. Benchmarking quantum chemistry methods for spin-state energetics of iron complexes against quantitative experimental data. *Phys. Chem. Chem. Phys.* **2019**, *21*, 4854–4870.

(S47) Radoń, M. Role of Spin States in Nitric Oxide Binding to Cobalt(II) and Manganese(II) Porphyrins. Is Tighter Binding Always Stronger? *Inorg. Chem.* **2015**, *54*, 5634–5645.

(S48) Lawson Daku, L. M.; Aquilante, F.; Robinson, T. W.; Hauser, A. Accurate Spin-State Energetics of Transition Metal Complexes. 1. CCSD(T), CASPT2, and DFT Study of $[M(NCH)_6]^{2+}$ (M = Fe, Co). *J. Chem. Theory Comput.* **2012**, *8*, 4216–4231.

(S49) Flöser, B. M.; Guo, Y.; Riplinger, C.; Tuczek, F.; Neese, F. Detailed Pair Natural Orbital-based Coupled Cluster Studies of Spin Crossover Energetics. *J. Chem. Theory Comput.* **2020**, *16*, 2224–2235.

(S50) Reimann, M.; Kaupp, M. Spin-State Splittings in 3d Transition-Metal Complexes Revisited: Benchmarking Approximate Methods for Adiabatic Spin-State Energy Differences in Fe(II) Complexes. *J. Chem. Theory Comput.* **2022**, *28*, 7442–7456.

(S51) Grimme, S.; Antony, J.; Ehrlich, S.; Krieg, H. A Consistent and Accurate ab Initio Parametrization of Density Functional Dispersion Correction (DFT-D) for the 94 Elements H–Pu. *J. Chem. Phys.* **2010**, *132*, 154104.

(S52) Grimme, S.; Ehrlich, S.; Goerigk, L. Effect of the damping function in dispersion corrected density functional theory. *J. Comp. Chem.* **2011**, *32*, 1456–1465.

(S53) Lehtola, S.; Steigemann, C.; Oliveira, M. J. T.; Marques, M. A. L. Recent developments in libxc – A comprehensive library of functionals for density functional theory. *SoftwareX* **2018**, *7*, 1–5.

(S54) DFT-D3 Ver. 3.2 Rev 0, Grimme's group, Universit"at Bonn, available from https://www.chemie.uni-bonn.de/grimme/de/software/dft-d3 (accessed September 2023). 2016.

(S55) Kepp, K. P. Theoretical Study of Spin Crossover in 30 Iron Complexes. *Inorg. Chem.* **2016**, *55*, 2717–2727.

(S56) Mejía-Rodríguez, D.; Trickey, S. B. Spin-Crossover from a Well-Behaved, Low-Cost meta-GGA Density Functional. *J. Phys. Chem. A* **2020**, *124*, 9889–9894.

(S57) Vydrov, O. A.; Van Voorhis, T. Nonlocal van der Waals density functional: The simpler the better. *J. Chem. Phys.* **2010**, *133*, 244103.

(S58) Eichkorn, K.; Treutler, O.; Öhm, H.; Häser, M.; Ahlrichs, R. Auxiliary basis sets to approximate Coulomb potentials. *Chemical Physics Letters* **1995**, *240*, 283–290.

(S59) Weigend, F. Accurate Coulomb-fitting basis sets for H to Rn. *Phys. Chem. Chem. Phys.* **2006**, *8*, 1057–1065.

(S60) Neese, F.; Wennmohs, F.; Hansen, A.; Becker, U. Efficient, approximate and parallel Hartree-Fock and hybrid DFT calculations. A 'chain-of-spheres' algorithm for the Hartree-Fock exchange. *Chem. Phys.* **2009**, *356*, 98–109.

(S61) Weigend, F.; Häser, M.; Patzelt, H.; Ahlrichs, R. RI-MP2: Optimized Auxiliary Basis Sets and Demonstration of Efficiency. *Chem. Phys. Lett.* **1998**, *294*, 143–152.

(S62) Aðalsteinsson, H. M.; Bjornsson, R. Ionization energies of metallocenes: a coupled cluster study of cobaltocene. *Phys. Chem. Chem. Phys.* **2023**, *25*, 4570–4587.

(S63) Yamaguchi, K.; Jensen, F.; Dorigo, A.; Houk, K. A spin correction procedure for unrestricted Hartree-Fock and Møller-Plesset wavefunctions for singlet diradicals and polyradicals. *Chem. Phys. Lett.* **1988**, *149*, 537–542.

(S64) Balabanov, N. B.; Peterson, K. A. Systematically convergent basis sets for transition metals. I. All-electron correlation consistent basis sets for the 3d elements Sc–Zn. *J. Chem. Phys.* **2005**, *123*, 064107.

(S65) Hill, J. G.; Platts, J. A. Auxiliary basis sets for density fitting–MP2 calculations: Nonrelativistic triple-ζ all-electron correlation consistent basis sets for the 3d elements Sc–Zn. *J. Chem. Phys.* **2008**, *128*, 044104.

(S66) Weigend, F.; Köhn, A.; Hättig, C. Efficient use of the correlation consistent basis sets in resolution of the identity MP2 calculations. *J. Chem. Phys.* **2002**, *116*, 3175–3183.

(S67) Weigend, F. Hartree–Fock exchange fitting basis sets for H to Rn. *J. Comp. Chem.* **2008**, *29*, 167–175.

(S68) Weigend, F. A fully direct RI-HF algorithm: Implementation, optimised auxiliary basis sets, demonstration of accuracy and efficiency. *Phys. Chem. Chem. Phys.* **2002**, *4*, 4285–4291.

(S69) Yousaf, K. E.; Peterson, K. A. Optimized complementary auxiliary basis sets for explicitly correlated methods: aug-cc-pVnZ orbital basis sets. *Chem. Phys. Lett.* **2009**, *476*, 303–307.

(S70) Hill, J. G.; Peterson, K. A. Gaussian basis sets for use in correlated molecular calculations. XI. Pseudopotential-based and all-electron relativistic basis sets for alkali metal (K-Fr) and alkaline earth (Ca-Ra) elements. *J. Chem. Phys.* **2017**, *147*, 244106.

(S71) Phung, Q. M.; Feldt, M.; Harvey, J. N.; Pierloot, K. Towards Highly Accurate Spin State Energetics in First-Row Transition Metal Complexes: A Combined CASPT2/CC Approach. *J. Chem. Theory Comput.* **2018**, *14*, 2446–2455.

(S72) Reimann, M.; Kaupp, M. Spin-State Splittings in 3d Transition-Metal Complexes Revisited: Toward a Reliable Theory Benchmark. *J. Chem. Theory Comput.* **2023**, *19*, 97–108.

(S73) Pierloot, K.; Phung, Q. M.; Domingo, A. Spin State Energetics in First-Row Transition Metal Complexes: Contribution of (3s3p) Correlation and its Description by Second-Order Perturbation Theory. *J. Chem. Theory Comput.* **2017**, *13*, 537–553.

(S74) Drabik, G.; Radoń, M. Approaching the Complete Basis Set Limit for Spin–State Energetics of Mononuclear First-Row Transition Metal Complexes. *J. Chem. Theory Comput.* **2024**, *20*, 3199–3217.

(S75) Jensen, F. *Introduction to Computational Chemistry*, 2nd ed.; Wiley, 2007.

(S76) Radoń, M. Benchmarks for transiton metal spin–state energetics: Why and how to employ experimental reference data? *Phys. Chem. Chem. Phys.* **2023**, *25*, 30800–30820.

(S77) Ribeiro, R. F.; Marenich, A. V.; Cramer, C. J.; Truhlar, D. G. Use of Solution-Phase Vibrational Frequencies in Continuum Models for the Free Energy of Solvation. *J. Phys. Chem. B* **2011**, *115*, 14556–14562.

(S78) Drabik, G.; Szklarzewicz, J.; Radoń, M. Spin–state energetics of metallocenes: How do best wave function and density functional theory results compare with the experimental data? *Phys. Chem. Chem. Phys.* **2021**, *23*, 151–172.

(S79) Lami, A.; Santoro, F. *Computational Strategies for Spectroscopy*; John Wiley & Sons, Ltd, 2011; Chapter 10, pp 475–516.

(S80) Avila Ferrer, F. J.; Cerezo, J.; Soto, J.; Improta, R.; Santoro, F. First-principle computation of absorption and fluorescence spectra in solution accounting for vibronic structure, temperature effects and solvent inhomogenous broadening. *Comput. Theor. Chem.* **2014**, *1040-1041*, 328 – 337, Excited states: From isolated molecules to complex environments.

(S81) Santoro, F.; Cerezo, J. FCclasses3, a code to simulate electronic spectra, v3, 2022. Visit `http://www.iccom.cnr.it/en/fcclasses/`.

(S82) Cerezo, J.; Zúñiga, J.; Requena, A.; Ávila Ferrer, F. J.; Santoro, F. Harmonic Models in Cartesian and Internal Coordinates to Simulate the Absorption Spectra of Carotenoids at Finite Temperatures. *J. Chem. Theory Comput.* **2013**, *9*, 4947–4958.

(S83) Cerezo, J.; Santoro, F. Revisiting Vertical Models To Simulate the Line Shape of Electronic Spectra Adopting Cartesian and Internal Coordinates. *J. Chem. Theory Comput.* **2016**, *12*, 4970–4985.

(S84) Bakken, V.; Helgaker, T. The efficient optimization of molecular geometries using redundant internal coordinates. *J. Chem. Phys.* **2002**, *117*, 9160.

(S85) Norjmaa, G.; Ujaque, G.; Lledós, A. Beyond Continuum Solvent Models in Computational Homogeneous Catalysis. *Top. Catal.* **2022**, *65*, 118–140.

(S86) Vela, S.; Fumanal, M.; Cirera, J.; Ribas-Arino, J. Thermal spin crossover in Fe(II) and Fe(III). Accurate spin state energetics at the solid state. *Phys. Chem. Chem. Phys.* **2020**, *22*, 4938–4945.

(S87) Cococcioni, M.; de Gironcoli, S. Linear response approach to the calculation of the effective interaction parameters in the LDA+U method. *Phys. Rev. B* **2005**, *71*, 035105.

(S88) Garrity, K. F.; Bennett, J. W.; Rabe, K. M.; Vanderbilt, D. Pseudopotentials for high-throughput DFT calculations. *Comp. Mat. Sci.* **2014**, *81*, 446 – 452.

(S89) Rat, S.; Ridier, K.; Vendier, L.; Molnár, G.; Salmon, L.; Bousseksou, A. Solvatomorphism and structural-spin crossover property relationship in bis[hydrotris(1,2,4-triazol-1-yl)borate]iron(ii). *CrystEngComm* **2017**, *19*, 3271–3280.

(S90) Holland, J. M.; McAllister, J. A.; Lu, Z.; Kilner, C. A.; Thornton-Pett, M.; Halcrow, M. A. An unusual abrupt thermal spin-state transition in [FeL][BF] [L = 2,6-di(pyrazol-1-yl)pyridine]. *Chem. Commun.* **2001**, 577–578.

(S91) Li, J.; Lord, R.; Noll, B.; Baik, M.-H.; Schulz, C.; Scheidt, W. Cyanide: A Strong-Field Ligand for Ferrohemes and Hemoproteins? *Angew. Chem. Int. Ed.* **2008**, *47*, 10144–10146.

(S92) Juhász, G.; Hayami, S.; Inoue, K.; Maeda, Y. [CoII(phimpy)$_2$](ClO$_4$)$_2$ and [CoII(ipimpy)$_2$](ClO$_4$)$_2$: New Cobalt(II) Spin Crossover Compounds, and the Role of the Ligand Flexibility in Spin Transition Behavior. *Chem. Lett.* **2003**, *32*, 882–883.

(S93) Makov, G.; Payne, M. C. Periodic boundary conditions in ab initio calculations. *Phys. Rev. B* **1995**, *51*, 4014–4022.

(S94) (a) Derenzo, S. E.; Klintenberg, M. K.; Weber, M. J. Determining point charge arrays that produce accurate ionic crystal fields for atomic cluster calculations. *J. Chem. Phys.*

2000, *112*, 2074; (b) Klintenberg, M.; Derenzo, S.; Weber, M. Accurate crystal fields for embedded cluster calculations. *Comp. Phys. Comm.* **2000**, *131*, 120–128.

(S95) Morioka, Y.; Toriumi, K.; Ito, T.; Saito, A.; Nakagawa, I. Crystal Structures of the Room- and Low-Temperature Phases of Monoclinic Potassium Ferricyanide. *J. Phys. Soc. Japan* **1985**, *54*, 2184–2189.

(S96) Willans, M. J.; Wasylishen, R. E.; McDonald, R. Polymorphism of Potassium Ferrocyanide Trihydrate as Studied by Solid-State Multinuclear NMR Spectroscopy and X-ray Diffraction. *Inorg. Chem.* **2009**, *48*, 4342–4353.

(S97) Ueda, T.; Bernard, G. M.; McDonald, R.; Wasylishen, R. E. Cobalt-59 NMR and X-ray diffraction studies of hydrated and dehydrated (±)-tris(ethylenediamine) cobalt(III) chloride. *Solid State Nucl. Magn. Res.* **2003**, *24*, 163–183.

(S98) Krüger, G. J.; Reynhardt, E. C. New investigation of the structure of trisacetylacetonatocobalt(III). *Acta Crystallographica Section B* **1974**, *30*, 822–824.

(S99) Diaz-Acosta, I.; Baker, J.; Cordes, W.; Pulay, P. Calculated and Experimental Geometries and Infrared Spectra of Metal Tris-Acetylacetonates: Vibrational Spectroscopy as a Probe of Molecular Structure for Ionic Complexes. Part I. *J. Phys. Chem. A* **2001**, *105*, 238–244.

(S100) Junk, P. C. Supramolecular interactions in the X-ray crystal structure of potassium tris(oxalato)ferrate(III) trihydrate. *J. Coord. Chem.* **2005**, *58*, 355–361.

(S101) Montgomery, H.; Morosin, B.; Natt, J. J.; Witkowska, A. M.; Lingafelter, E. C. The crystal structure of Tutton's salts. VI. Vanadium(II), iron(II) and cobalt(II) ammonium sulfate hexahydrates. *Acta Crystallographica* **1967**, *22*, 775–780.

(S102) Lee, T. J.; Taylor, P. R. A diagnostic for determining the quality of single-reference electron correlation methods. *Int. J. Quantum Chem.* **1989**, *36*, 199–207.

(S103) Lee, T. J.; Rice, J. E.; Scuseria, G. E.; Schaefer, H. F. Theoretical investigations of molecules composed only of fluorine, oxygen and nitrogen: determination of the equilibrium structures of FOOF, (NO)2 and FNNF and the transition state structure for FNNF cis-trans isomerization. *Theor. Chim. Acta* **1989**, *75*, 81–98.

(S104) Janssen, C. L.; Nielsen, I. M. B. New diagnostics for coupled-cluster and Møller-Plesset perturbation theory. *Chem. Phys. Lett.* **1998**, *290*, 423–430.

(S105) Nielsen, I.; Janssen, C. Double-substitution-based diagnostics for coupled-cluster and Moller-Plesset perturbation theory. *Chem. Phys. Lett.* **1999**, *310*, 568–576.

(S106) Tishchenko, O.; Zheng, J.; Truhlar, D. G. Multireference Model Chemistries for Thermochemical Kinetics. *J. Chem. Theory Comput.* **2008**, *4*, 1208–1219.

(S107) Schultz, N. E.; Zhao, Y.; Truhlar, D. G. Density Functionals for Inorganometallic and Organometallic Chemistry. *J. Phys. Chem. A* **2005**, *109*, 11127–11143.

(S108) Fogueri, U. R.; Kozuch, S.; Karton, A.; Martin, J. M. L. A simple DFT-based diagnostic for nondynamical correlation. *Theor. Chem. Acc.* **2012**, *132*, 1291.

(S109) Karton, A.; Rabinovich, E.; Martin, J. M. L.; Ruscic, B. W4 theory for computational thermochemistry: In pursuit of confident sub-kJ/mol predictions. *J. Chem. Phys.* **2006**, *125*, 144108.

(S110) Karton, A.; Daon, S.; Martin, J. M. W4-11: A high-confidence benchmark dataset for computational thermochemistry derived from first-principles {W4} data. *Chem. Phys. Lett.* **2011**, *510*, 165–178.
